# Qing`e Pill Inhibits Osteoblast Ferroptosis *via* ATM Serine/Threonine Kinase (ATM) and the PI3K/AKT Pathway in Primary Osteoporosis

**DOI:** 10.3389/fphar.2022.902102

**Published:** 2022-07-05

**Authors:** Jian Hao, Jiaxin Bei, Zhenhan Li, Mingyuan Han, Boyuan Ma, Pengyi Ma, Xianhu Zhou

**Affiliations:** ^1^ Orthopedics Department, The Affiliated Hospital of Medical School, Ningbo University, Ningbo, China; ^2^ Laboratory of Interventional Radiology, Department of Minimally Invasive Interventional Radiology and Department of Radiology, The Second Affiliated Hospital of Guangzhou Medical University, Guangzhou, China; ^3^ School of Clinical, Wannan Medical College, Wuhu, China; ^4^ Department of Orthopaedic, Graduate School, Tianjin Medical University, Tianjin, China

**Keywords:** osteoporosis, PI3K/AKT pathway, ATM, ferroptosis, network analysis, Qing'e Pill

## Abstract

Osteoporosis (OP) is an aging-related disease that is the main etiology of fragility fracture. Qing’e Pill (QEP) is a mixture of traditional Chinese medicine (TCM) consisting of *Eucommia ulmoides* Oliv., *Psoralea corylifolia* L., *Juglans regia* L., and *Allium sativum* L. QEP has an anti-osteoporosis function, but the underlying mechanism remains unclear. In this study, online databases were employed to determine the chemical compounds of QEP and potential target genes in osteoporosis. Potential pathways associated with genes were defined by Gene Ontology (GO) and Kyoto Encyclopaedia of Genes and Genomes (KEGG) databases. A compound–target–disease network was constructed. Hub genes screened through Cytoscape were intersected with the FerrDB database. The potential key genes were validated in HFOB 1.19 cells, and rat models were ovariectomized through Western blot, RT-qPCR, ELISA, HE staining, immunohistochemistry, and immunofluorescence analyses. The intersection targets of QEP and osteoporosis contained 121 proteins, whereas the target–pathway network included 156 pathways. We filtered five genes that stood out in the network analysis for experimental verification. The experiments validated that QEP exerted therapeutic effects on osteoporosis by inhibiting ferroptosis and promoting cell survival *via* the PI3K/AKT pathway and ATM. In conclusion, combining the application of network analysis and experimental verification may provide an efficient method to validate the molecular mechanism of QEP on osteoporosis.

## Introduction

Osteoporosis (OP) is the most common bone disease, and approximately, more than 200 million people worldwide suffer from it. The coordination and tight interplay of osteoblasts and osteoclasts play an indispensable role in the occurrence and development of bone tissues. The imbalance between them directly affects the density and strength of the bones, which will further lead to osteoporosis and increase the risk of fractures. Thus, promoting osteogenic differentiation and bone-forming osteoblast cells is a reliable approach for osteoporosis treatment (Zhang et al., 2021).

Oxidative stress is highly related to postmenopausal OP (Li et al., 2020). Iron is one of the most abundant elements in the human body and is mainly responsible for producing reactive oxygen species (ROS) and causing oxidative damage (Xie et al., 2016; Sui et al., 2018; Park and Chung, 2019; Ma et al., 2020).

Ferroptosis is a recently discovered type of programmed cell death caused by iron overload. Some studies have indicated that iron overload contributes to the process of OP by inducing oxidative damages (Presa et al., 2018; Tian et al., 2020). The potential relationship between ferroptosis and OP is highly concerned (Lu et al., 2019; Ma et al., 2020; Yang et al., 2021; Wang et al., 2022). According to previous studies, the AKT/PI3K pathway is responsible for improving the condition of osteoporosis by promoting the proliferation and migration ability of bone marrow stem cells (Wang et al., 2020; Zhao F. et al., 2021).

QEP is a Chinese traditional medicine formula composed of four botanical drugs, namely, *E. ulmoides* Oliv., *P. corylifolia* L., *J. regia* L., and *A. sativum* L. It has a strong antioxidant activity to reverse ovariectomy-derived atrophy in the uterus and menopausal-derived lipid metabolism dysfunction (Yang et al., 2015; Xiong et al., 2022). Moreover, it could increase sclerostin expression, osteogenic proteins (like β-catenin and Runx2), bone calcium absorption, calcium and phosphorus balance, and bone metabolism (Li et al., 2017; Lu et al., 2018; Sun et al., 2020). These observations suggest that QEP improves some aspects of pathological manifestations in postmenopausal osteoporosis, but the underlying mechanism remains unclear. The present study aimed to determine the mechanism through which QEP ameliorates OP.

A dual strategy of network analysis and experiment verification was performed to study the detailed mechanism of QEP in the treatment of osteoporosis. First, the monomers of the four TCM components in the pill were queried separately, and the potential drug monomers were selected after screening. The internet pharmacology website was used to find drug targets and disease targets and to screen key potential targets of QEP on OP. Gene Ontology (GO)/Kyoto Encyclopaedia of Genes and Genomes (KEGG) enrichment analysis was performed, and we found the important terms were oxidative stress, iron metabolism, and lipid metabolism which are features of ferroptosis. We hypothesized that QEP may inhibit ferroptosis in OP. Cell-level and animal-level experiments were performed to verify the mechanism of QEP on OP. Our results confirmed that QEP has a curative effect on osteoporosis *in vivo* and verified the involvement of ATM and the AKT/PI3K pathway in improving osteogenesis and inhibiting the ferroptosis of osteoblasts.

## Materials and Methods

### Research Design Instruction

With its complex components and functional diversity, traditional Chinese medicine has been a difficult research subject. According to “Best Practices in Research—Overcoming Common Challenges in Phytopharmacological Research,” we basically followed the guideline on how to perform multiherbal (complex) research. In our study, the screened chemical compounds of QEP were identified through online public databases; animal administration was adjusted according to the clinical dose (Heinrich et al., 2020).

### Traditional Chinese Medicine Systems Pharmacology Database and Analysis Platform and Bioinformatics Analysis Tool for Molecular mechANism Database

Traditional Chinese Medicine Systems Pharmacology database and Analysis Platform (TCMSP) and Bioinformatics Analysis Tool for Molecular mechANism (BATMAN) are powerful and user-friendly analysis websites designed to study the molecular mechanisms of Chinese medicine. In BATMAN, we collected the drug targets of QEP for follow-up research, and TCMSP was used to supplement drug targets and obtain small drug molecules. In screening small drug molecules, we followed the threshold values of oral bioavailability (OB) ≥30% and drug likeness (DL) ≥0.18. The screened small molecules were searched in the PubChem compound database (https://www.ncbi.nlm.nih.gov/pccompound) to acquire the molecular formula and molecular structure.

### GeneCards and DisGeNET Database

GeneCards and DisGeNET are convenient websites that integrate multiple omics, genetics, clinical data, etc., with genes as the center. We used osteoporosis as the keyword to search on the websites and in the GeneCards database. Genes with scores greater than 1 were screened. When we obtained the results with osteoporosis as the keyword from the two websites, we merged the two gene sets, deleted the duplicate genes, and obtained the disease gene set.

### FerrDB

FerrDB is the world’s first efficient database for sorting ferroptosis regulators. We conveniently obtained the authoritative ferroptosis gene set from the website.

### Gene Ontology and Kyoto Encyclopedia of Genes and Genomes

GO/KEGG enrichment analysis is an efficient method used to fully explore potential molecular functions and signal pathways in many genes. We set the intersection of drug targets and disease targets as the potential therapeutic target and used R (version 3.6.3) software and clusterProfiler package for enrichment analysis. *Homo sapiens* was selected as the species.

### Preparation of QEP Extracts

Prof. Yang Lei of Tianjin Nankai Hospital Affiliated Nankai University provided four botanical drugs of the QEP which are used and stored under the guidance of Chinese Pharmacopeia 2020 edition. The extracts of different preparations of QEPs were obtained by the following process. First, each ingredient, namely, *E. ulmoides* Oliv., *P. corylifolia* L., *J. regia* L., and *A. sativum* L., of QEP was weighed separately (50.0 g). The samples were decocted twice for 1 h at each time with water in a ratio of 1:8. Second, the decoction was collected and filtered using a filter paper. Third, the filtrate was extracted again twice for 1 h with 95% ethanol under reflux conditions. Finally, the two parts of filtrates were mixed, concentrated to a density of 1.0 g/ml in a rotating evaporator (Buchi, B-480) at 55°C and then stored at −20°C until use. The extract rate is 21%. We totally obtained 42 g extract. A proper amount of the extract was diluted with a medium and dissolved fully. The supernatant was filtered with a 0.22-μm filter membrane as the extract solution (Lu et al., 2018). The final concentration of QEP in applied solutions for cell culture was 20 mg/ml.

### Phytochemical Analysis of QEP

Phytochemical analysis of QEP was performed using an Agilent 1260 Infinity LC HPLC system (Agilent, United States) and separated on an ODS-2 Hypersil C-18 column (250 mm × 4.6 mm id, 5 μm; Thermo Scientific, Thermo Fisher Scientific Inc., United States) at 30°C. The catalysts used were 0.1% formic acid aqueous solution (A) and acetonitrile (B). The gradient elution program was as follows: 0–8 min, 5%–5% B; 8–10 min, 5%–8% B; 10–25 min, 8%–8% B; 25–30 min, 8%–20% B; 30–40 min, 20%–30% B; 40–60 min, 30%–70% B; and 60–80 min, 70%–70% B. The flow rate was 1.0 ml/min, and the absorption wavelength was below 246 nm.

### Alizarin Red Staining (ARS)

hFOB 1.19 cells were induced to undergo osteogenic differentiation for 9 days. The cells were washed, fixed with 70% ethanol for 10 min, and stained with 0.5% Alizarin red S (pH 4.2, Sigma-Aldrich) for 10 min. The stained cells were washed three times with deionized water. Red staining indicated the position and intensity of calcium deposits.

### Cell Culture and Reagents

Human hFOB 1.19 osteoblast cells were purchased from the American Type Culture Collection (ATCC) and cultured in MEM-α (Invitrogen, Inc., United States, 10370070) at 34.5°C in 5% CO_2_, according to ATCC protocols.

### Western Blot

Western blot analysis was performed routinely. Cells were seeded in culture plates under different conditions. All cells were washed with PBS buffer and lysed by lysis buffer (Beyotime Institute of Biotechnology). Protein concentrations were measured using the BCA method (Invitrogen Inc., United States). Equal quantities (30 μg) of proteins were loaded and separated on 10% SDS–polyacrylamide gels and then transferred onto PVDF membranes. The cells were blocked by TBST containing 5% skim milk at RT for 1 h and washed three times. The cells were incubated with primary antibodies for blots at 4°C overnight. All the primary antibodies used are listed as follows:

p-PI3K (Abcam, United States, Ab278545)
p-AKT (Abcam, United States, Ab38449)
p-mTOR (Abcam, United States, Ab109268)
GPX4 (EterLife, Birmingham, UK, EL604340)
xCT (EterLife, Birmingham, UK, EL611069)
4HNE (EterLife, Birmingham, UK, EL908789)
ATM (Abcam, United States, Ab201022)
Runx2 (Abcam, United States, Ab236639)
BMP2 (EterLife, Birmingham, UK, EL601094)

The blots were then washed in TBST and incubated with secondary antibodies at RT for 2 h. The blots were washed in TBST. The immunoreactive bands were visualized by chemiluminescence using enhanced chemiluminescence plus (GE Healthcare). GAPDH was used as an internal marker. The protein level was expressed as the ratio of the target band gray value to that of GADPH.

### ELISA

The ALP activity of hFOB 1.19 cells was detected through the hydrolysis of p-nitrophenyl phosphate (substrate) into p-nitrophenol at 37°C with pH 10.3 by using an ELISA kit (Nanjing Jiancheng Bioengineering Institute, Nanjing, China). ELISA kits for malondialdehyde (MDA, YT003523-48T), bone alkaline phosphatase (BALP, YT003336-48T), type I collagen C-terminal peptide (CTX-I, YT003456-48T), type I collagen N-terminal peptide (NTX, YT003013-48T), testosterone (T, YT003298-48T), glutathione (GSH, YT003323-48T), osteoprotegerin (OPG, YT002852-48T), osteocalcitin (BGP, YT003469-48T), calcitonin (CT, YT003292-48T), and growth hormone (GH, YT003346-48T) were purchased from Tianjin Itt Life Science R&D Co., Ltd.

### Reverse Transcription-Quantitative Polymerase Chain Reaction

Cells were seeded in culture plates under different conditions and cultured for different periods, as indicated in the table as follows. Total RNA was extracted from the cells by using the TRIzol reagent (TaKaRa Bio, Inc., Otsu, Japan). Reverse transcription was performed using the Transgen kit (AT-401). For the reverse transcription-quantitative polymerase chain reaction (RT-qPCR), the pre-designed primers are listed as follows:

BMP2 Forward: GTG​GGA​AAA​CAA​CCC​GGA​GA
Reverse: TTC​CCA​CCT​GCT​TGC​ATT​CT
GPX4 Forward: ACA​CCG​TCT​CTC​CAC​AGT​TC
Reverse: ACG​CTG​GAT​TTT​CGG​GTC​TG
xCT Forward: ACA​GGG​ATT​GGC​TTC​GTC​AT
Reverse: ATC​CCT​ATT​TTG​TGT​CTC​CCC​T
ATM Forward: GCG​TGG​CTA​ACG​GAG​AAA​AG
Reverse: ATC​ACT​GTC​ACT​GCA​CTC​GG
AKT Forward: CGAAGACGGGAGCAGGC
Reverse: CAG​CCA​ACC​CTC​CTT​CAC​AA
mTOR Forward: GCC​GCG​CGA​ATA​TTA​AAG​GA
Reverse: CTG​GTT​TCC​TCA​TTC​CGG​CT
PI3K Forward: TAG​CAG​CAT​GGT​CAG​CTT​TCT
Reverse: TGG​AAG​ACG​GGA​GAT​TCA​CAT
β-actin Forward: GGGCCGGACTCGTCATAC
Reverse: CCTGGCACCCAGCACAAT

SYBR Green Master Mix [Transgen (AT-601)] was combined according to the kit’s instructions. qPCR was performed on Thermo A7500 qPCR. Protein bands were obtained, and ImageJ software was used to calculate the gray value.

### Establishment of the Ferroptosis Cell Model

A total of 0.5 × 10^6^ hFOB 1.19 cells were plated in a 24-well plate. The cells were added with 20 μM erastin (S7242, Selleckchem) to induce ferroptosis cell death. EdU assay was performed using the EdU kit (BeyoClickTM, EDU-488, China) and Cell Counting Kit 8 (CCK8, Dojindo, Japan), according to the manufacturer’s instructions.

### Metascape

Metascape is a very powerful gene function analysis tool. For the given gene list, pathway and process enrichment analyses were carried out with the following ontology sources: KEGG functional sets, KEGG pathway, GO biological processes, GO cellular components, GO molecular functions, Reactome gene sets, CORUM, TRRUST, DisGeNET, PaGenBase, transcription factor targets, and COVID. The Molecular Complex Detection (MCODE) algorithm was applied to identify densely connected network components.

### STRING

STRING has collected hundreds of millions of interactions between proteins in many species. We have built a protein interaction network between potential therapeutic targets to explore connections by using STRING in *H. sapiens*.

### Cytoscape

Cytoscape is a piece of visualization software designed specifically for biological research, with powerful functions that can visualize complex networks and analyze molecular interaction networks. The molecular interaction network obtained from the STRING website was imported into Cytoscape. Each gene in the network was assigned a value through a topological network algorithm using the cytoHubba plug-in, and the key genes were found by sorting.

### Animals

Sixty rats were obtained from the Experimental Animal Centre of the Academy of Military Medical Sciences, Beijing, China [License No. SCXK (Jin) 2020-0008]. All animals were kept under standard conditions and given access to water and food ad libitum (Ethics Approval No. 202001). All rats were randomly divided into three groups. Forty rats received oophorectomy and were used to make an OVX-induced postmenopausal osteoporosis model, while 20 other rats received only sham operation. After 1 month, 40 OVX rats were administered with saline or QEP by gavage administration (4.5 g/kg/day) separately for 2 weeks in different groups. The other 20 rats were treated with saline. Finally, the rats were anesthetized and sacrificed, and tibias were obtained for different stains.

### Statistical Analysis

Experimental data were presented as mean ± standard deviation (mean ± SD). Statistical analyses were performed using GraphPad Prism 8.0 program (La Jolla, CA, United States). A one-way analysis of variance (ANOVA) was used to compare differences between groups. *p* < 0.05 was considered to indicate statistical significance.

## Results

### Molecular Components of QEP and Drug–Osteoporosis Co-targets

Thirty-six components were obtained using the screen method for further study, and detailed information was obtained from the PubChem compound database (https://www.ncbi.nlm.nih.gov/pccompound) (Table 1). We searched BATMAN to obtain four drug targets of *E. ulmoides* Oliv., *P. corylifolia* L., *J. regia* L., and *A. sativum* L. Given that the number of targets in *J. regia* L. was very negligible, we further used TCMSP to add some targets and avoid potential bias created by few samples (Ru et al., 2014). After summarizing the targets of the four traditional Chinese medicines and removing the duplications, the total number of drug targets reached 593 (Supplementary Table S1). We used osteoporosis as a keyword to search for related targets, and all genes with scores greater than 1 were obtained (Piñero et al., 2020; Barshir et al., 2021). After merging the hit lists and removing duplicates, 1770 unique hits remained, which were disease targets; 121 shared interactors commonly existed in 593 drug targets and 1770 disease targets (Shannon et al., 2003) (Supplementary Table S2). In theory, these targets could act as potential therapeutic targets of QEP for osteoporosis (Supplementary Table S3).

**TABLE 1:** Chemical compound information of QEP.

Molecule name	Molecular formula	Molecular structure	Botanical drug
Allicin	C_6_H_10_OS_2_	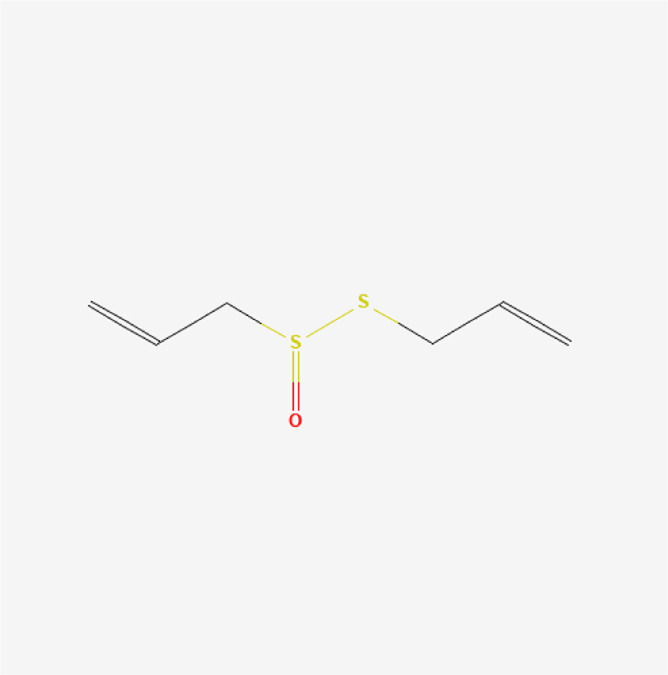	*A. sativum* L.
			
			
			
N-methylmescaline	C_12_H_19_NO_3_	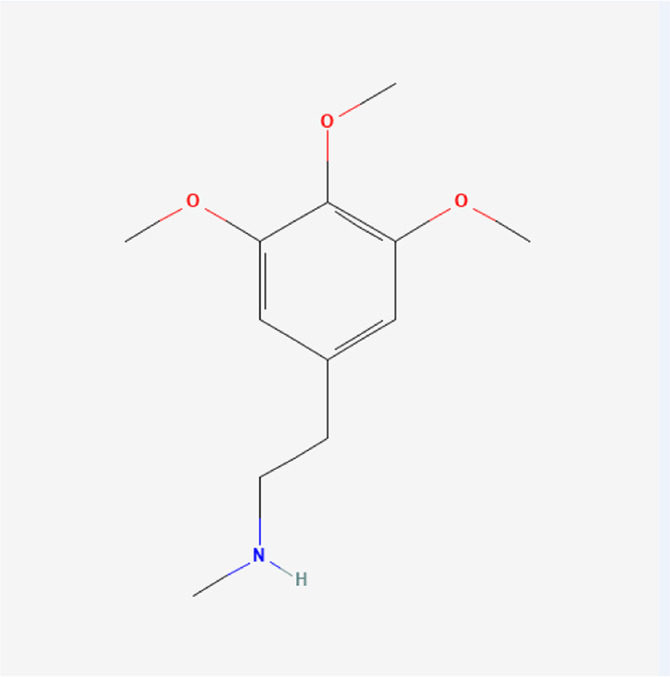	*A. sativum* L.
			
			
			
Methyl protodioscin	C_52_H_86_O_22_	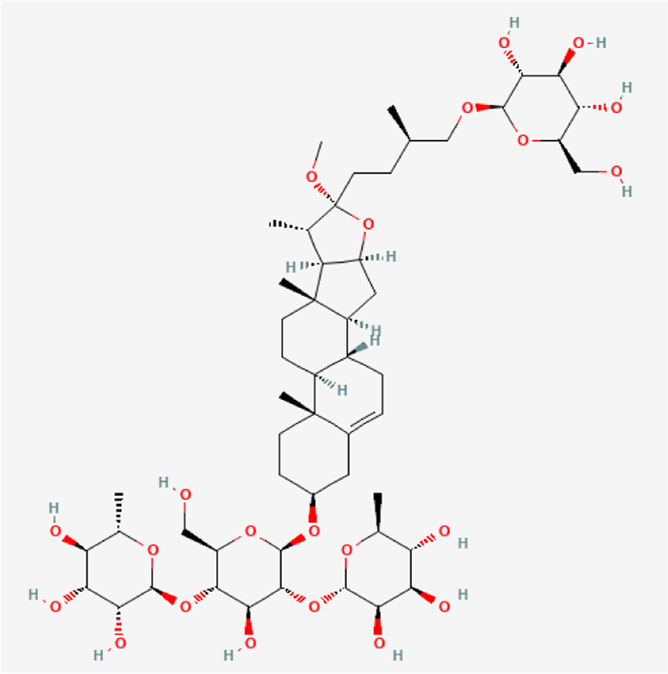	*A. sativum* L.
Pentanal	C_5_H_10_O	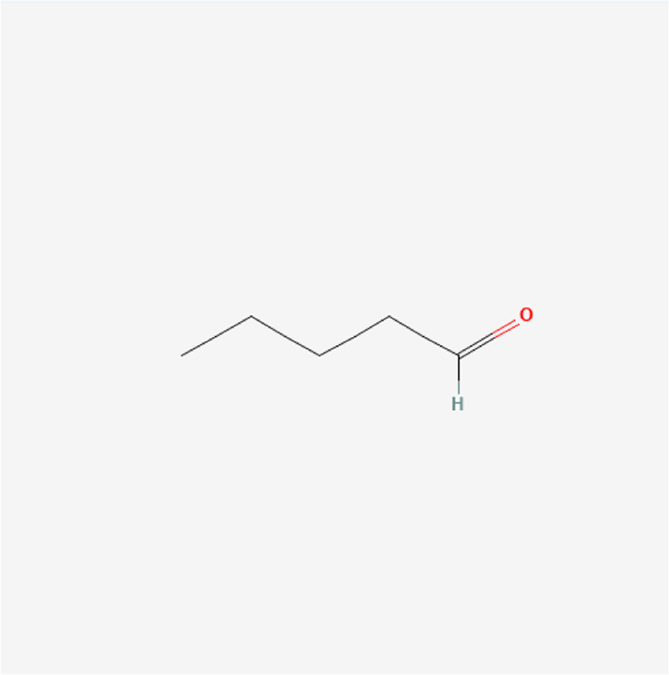	*A. sativum* L.
			
			
			
N-methylcytisine	C_12_H_16_N_2_O	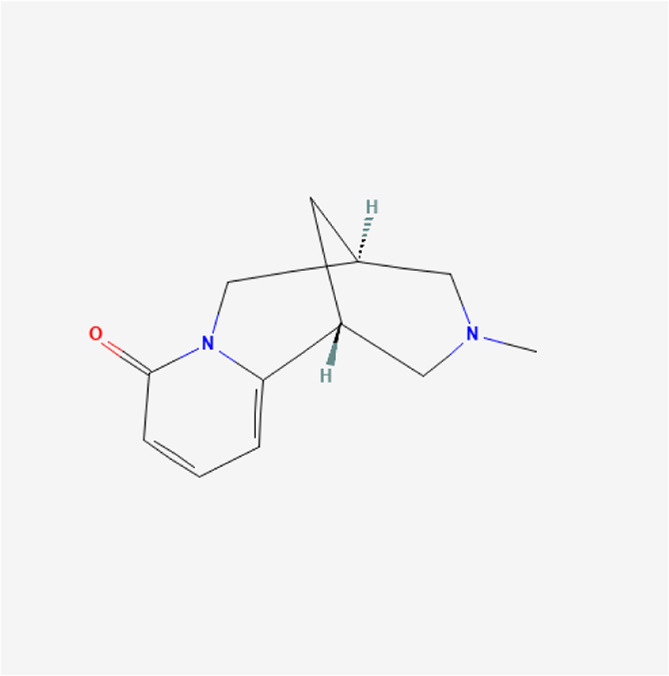	*A. sativum* L.
			
			
			
2-Methyl-dodecane-5-one	C_13_H_26_O	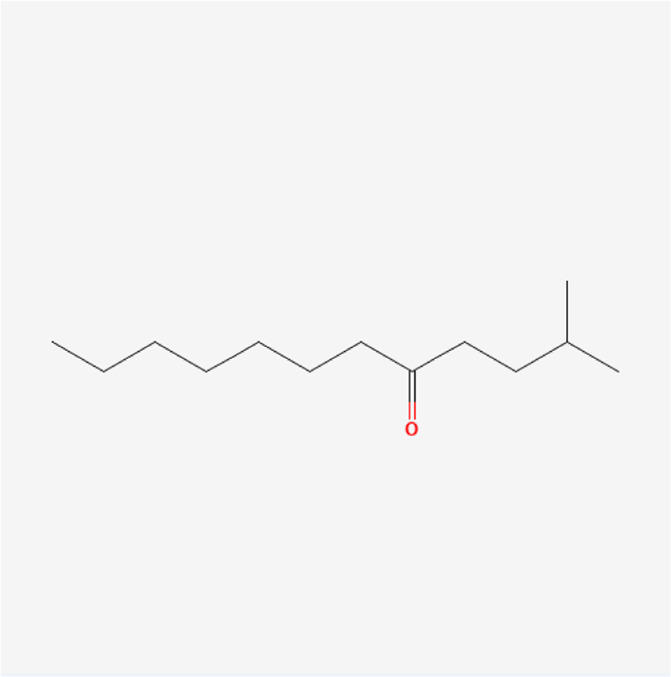	*A. sativum* L.
Dipterocarpol	C_30_H_50_O_2_	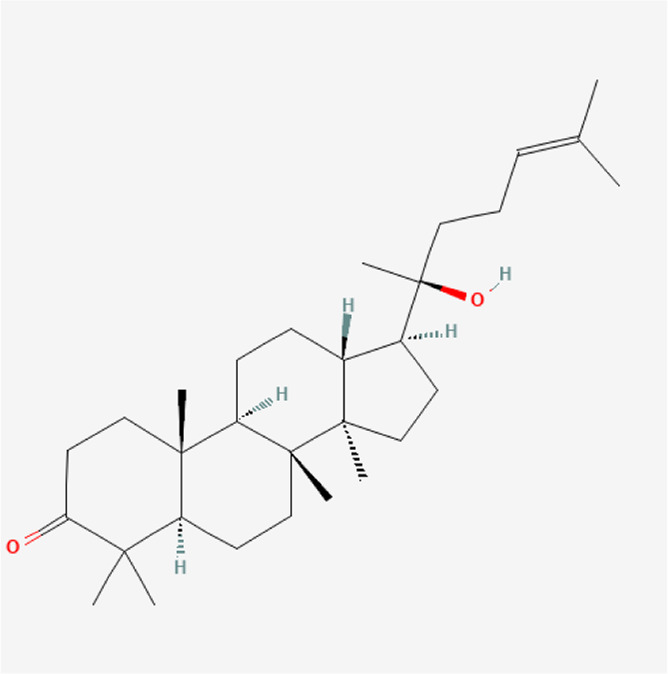	*A. sativum* L.
			
			
			
Divostroside	C_30_H_46_O_8_	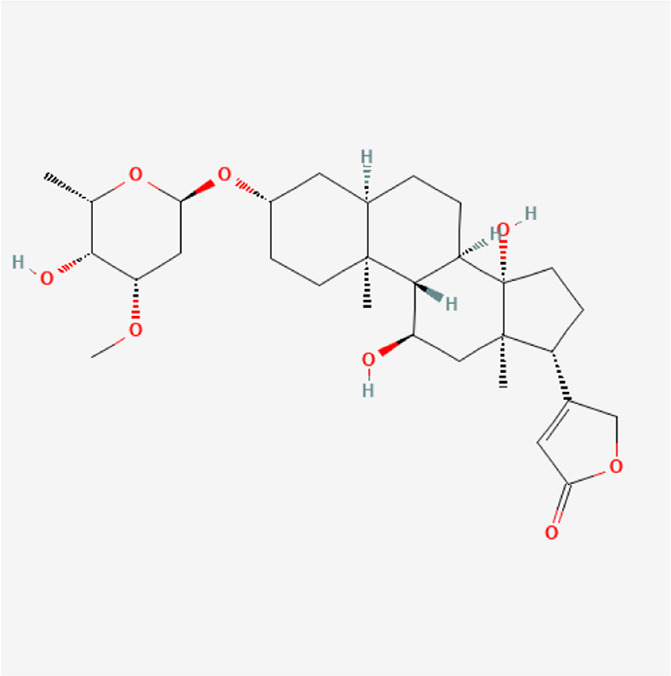	*A. sativum* L.
			
			
			
Sulfurenic acid	C_31_H_50_O_4_	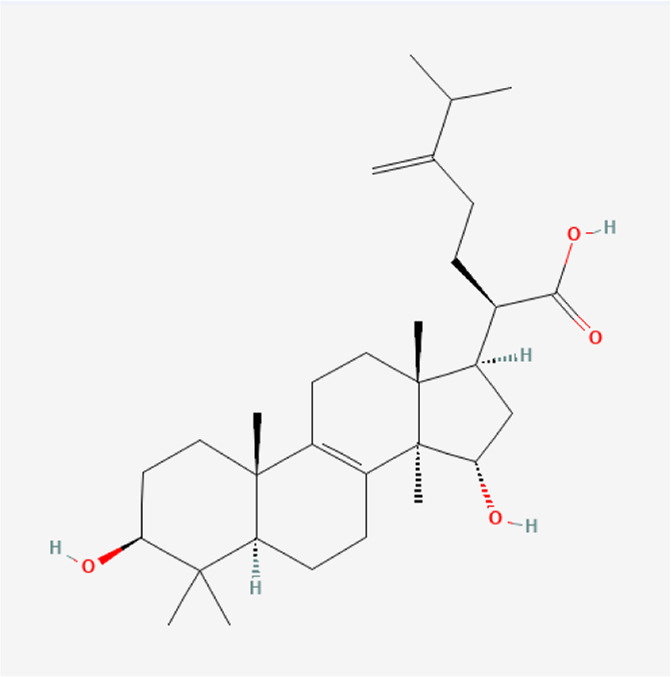	*A. sativum* L.
Geraniol	C_10_H_18_O	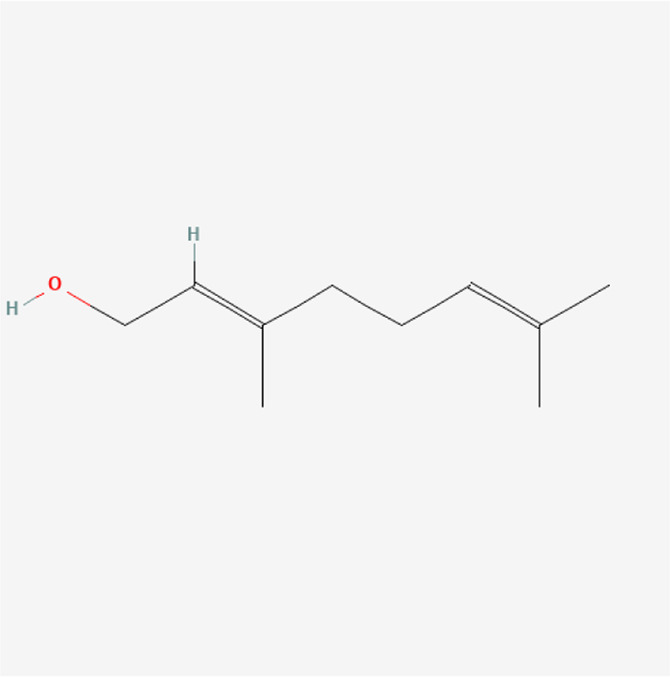	*A. sativum* L.
			
			
			
Protopanaxadiol	C_30_H_52_O_3_	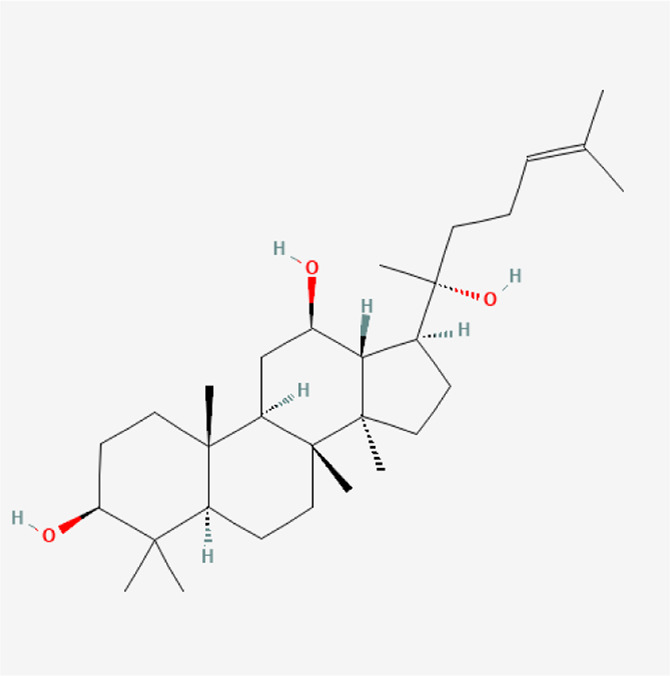	*A. sativum* L.
			
			
			
Citraurin beta	C_30_H_40_O_2_	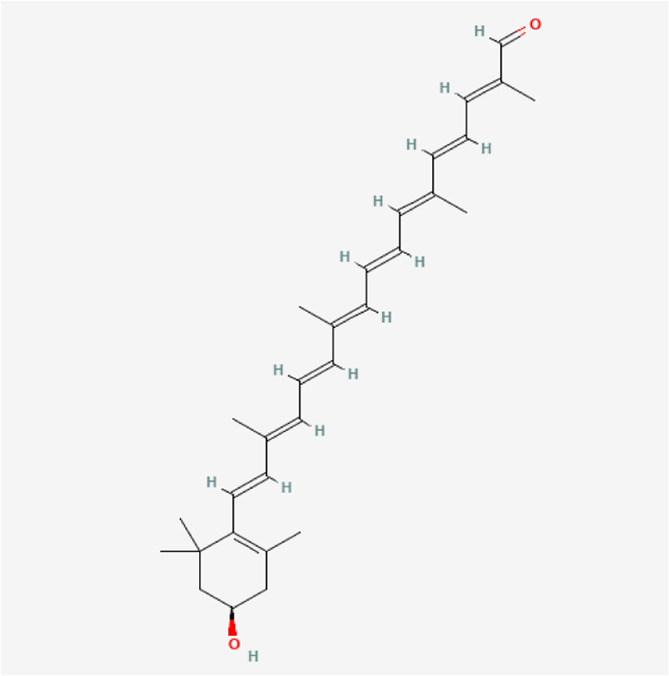	*A. sativum* L.
S-methyl-L-cysteine sulfoxide	C_4_H_9_NO_3_S	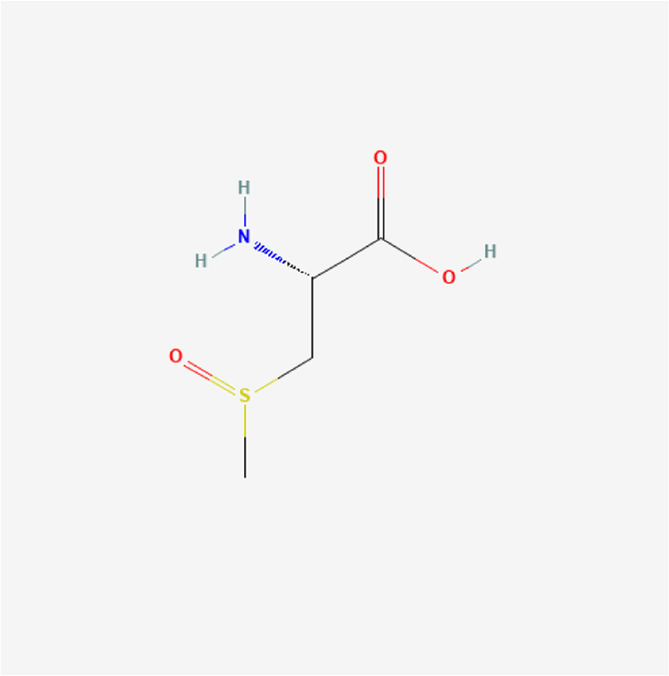	*A. sativum* L.
			
			
			
L-alpha, gamma-diaminobutyric acid	C_4_H_10_N_2_O_2_	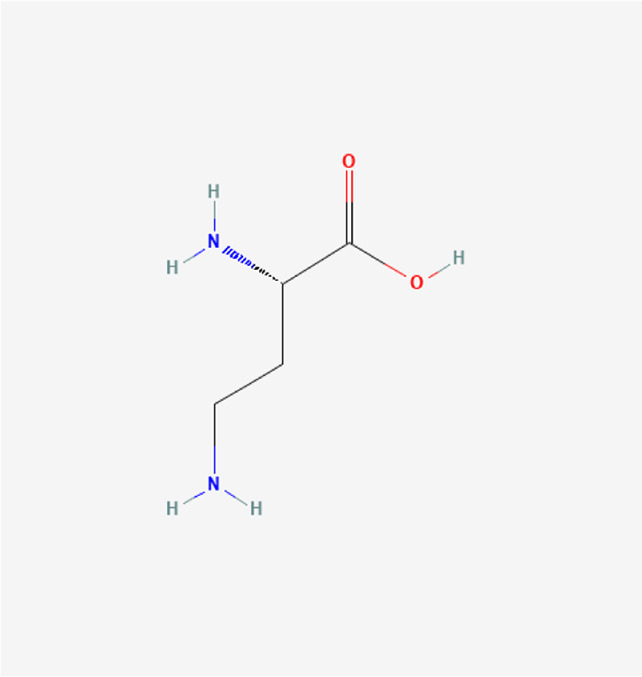	*A. sativum* L.
			
			
			
-Medioresinol di-O-beta-D-glucopyranoside	C_33_H_44_O_17_	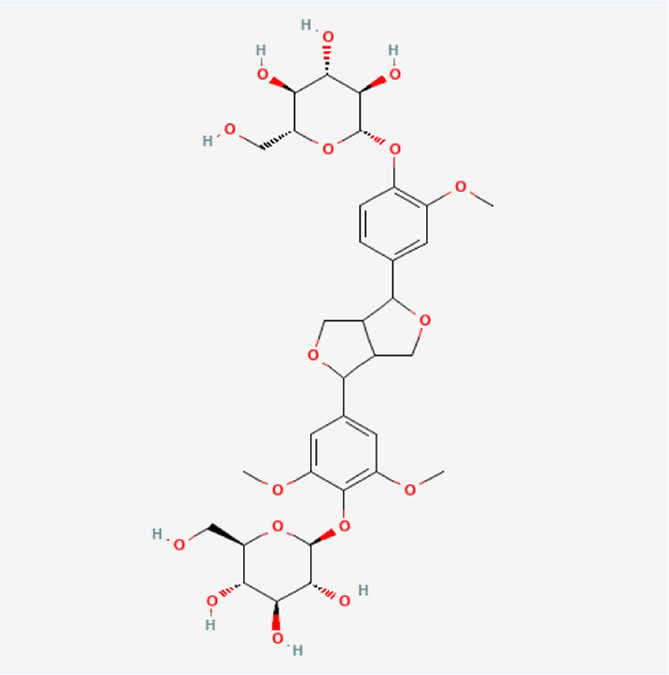	*E. ulmoides Oliv*
Dunnisinin	C_11_H_14_O_5_	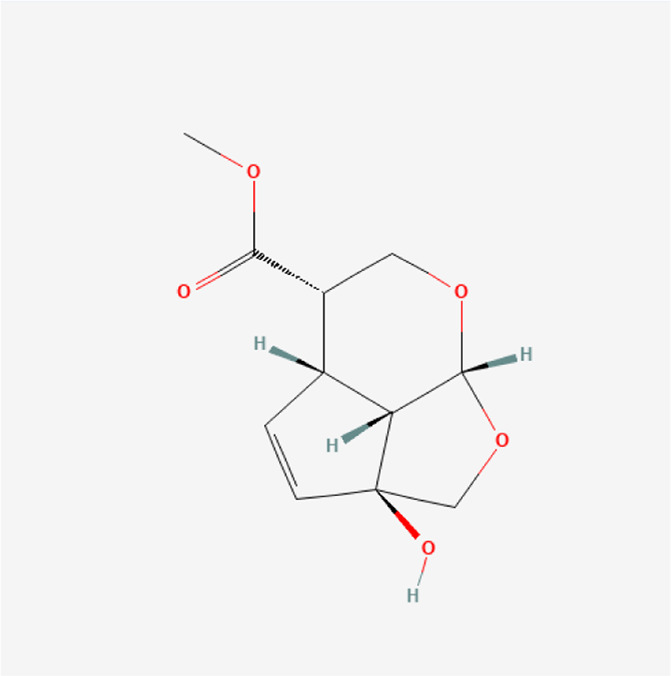	*E. ulmoides Oliv*
			
			
			
Eucommioside	C_15_H_26_O_9_	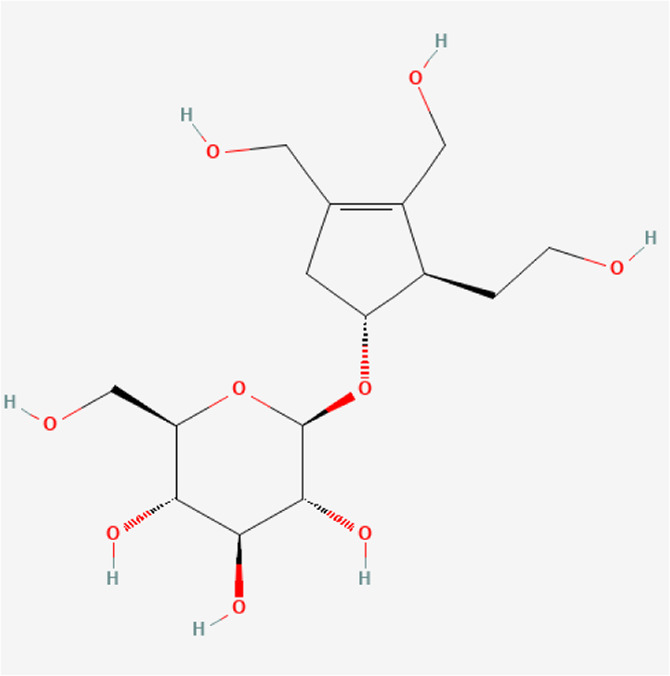	*E. ulmoides Oliv*
			
			
			
Dulcitol	C_6_H_14_O_6_	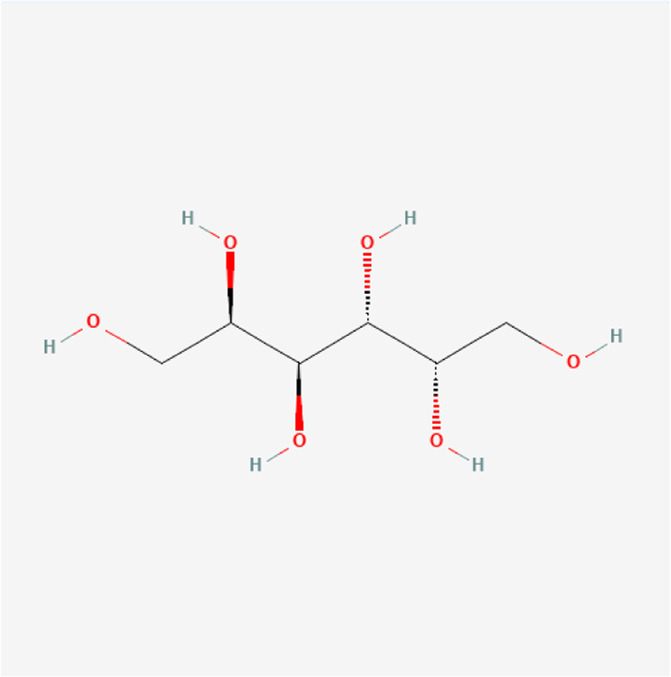	*E. ulmoides Oliv*
Erythro-beta-hydroxy-L-aspartic acid	C_4_H_7_NO_5_	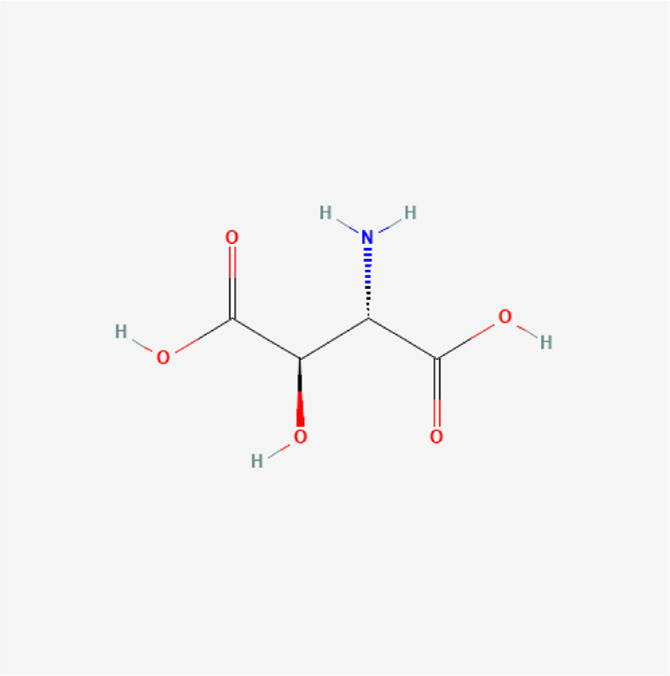	*E. ulmoides Oliv*
			
			
			
Isomaltose	C_12_H_22_O_11_	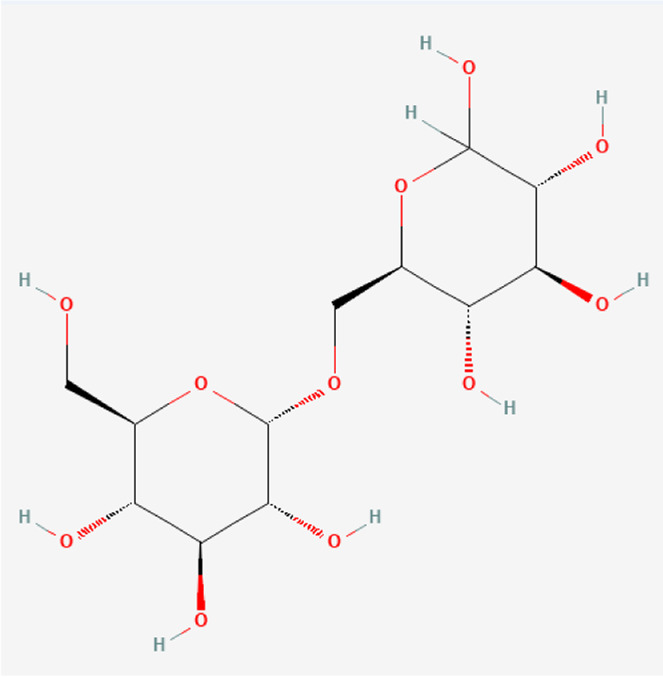	*E. ulmoides Oliv*
			
			
			
Caffeic acid dimethyl ether	C_11_H_12_O_4_	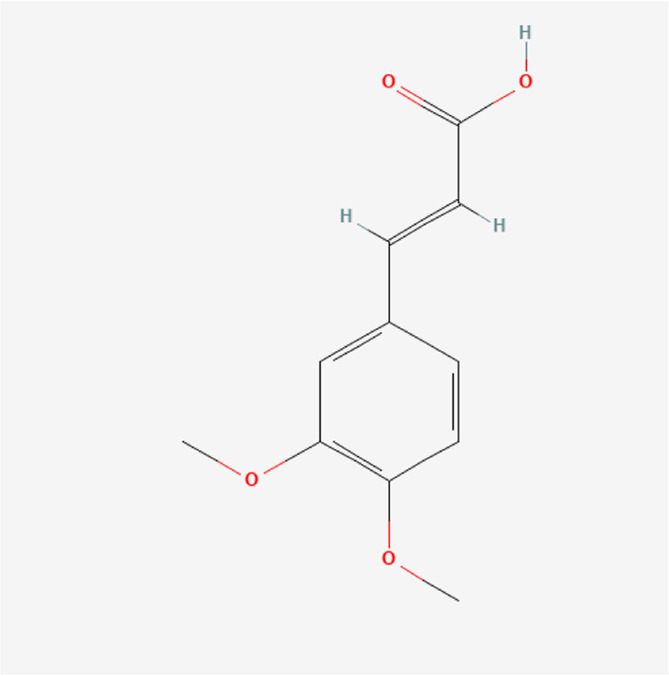	*E. ulmoides Oliv*
Threo-5-N-pentyl-4-hydroxy tetrahydrofuran-2-one	C_9_H_16_O_3_	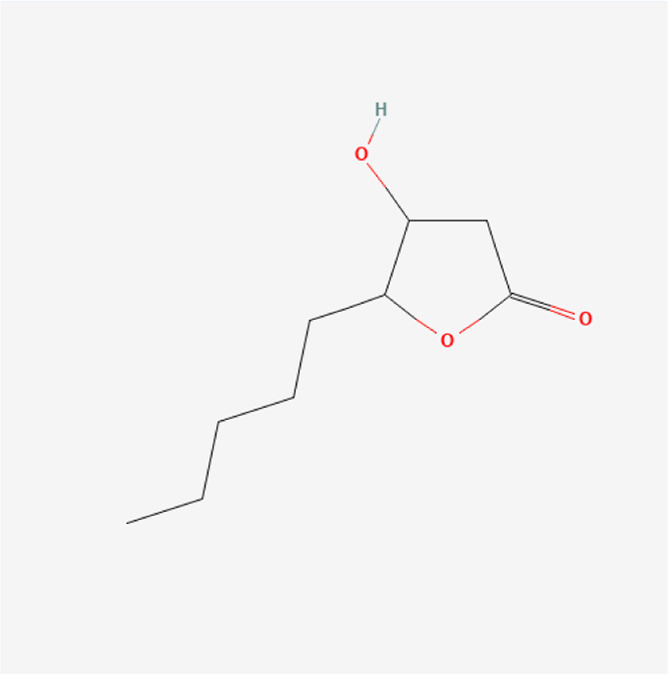	*E. ulmoides Oliv*
			
			
			
Kobusone	C_14_H_22_O_2_	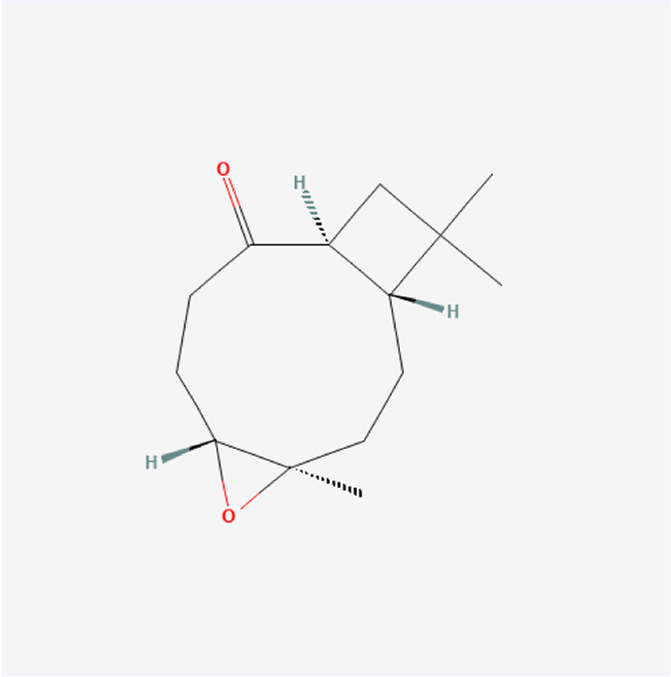	*E. ulmoides Oliv*
			
			
			
Eucommiol	C_9_H_16_O_4_	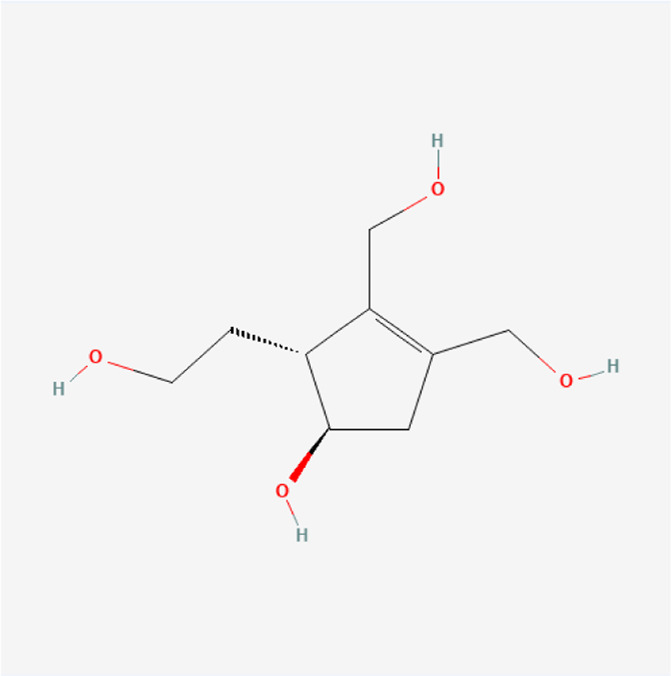	*E. ulmoides Oliv*
Civetone	C_17_H_30_O	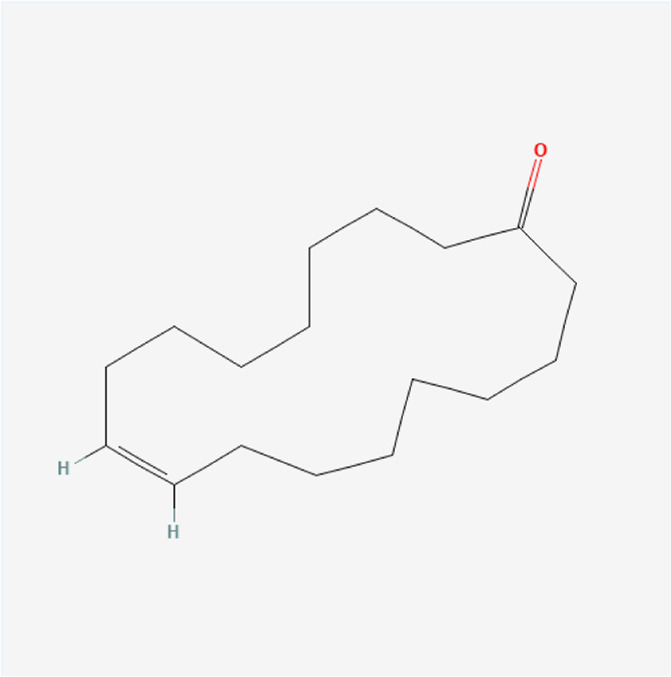	*E. ulmoides Oliv*
			
			
			
Juglone	C_10_H_6_O_3_	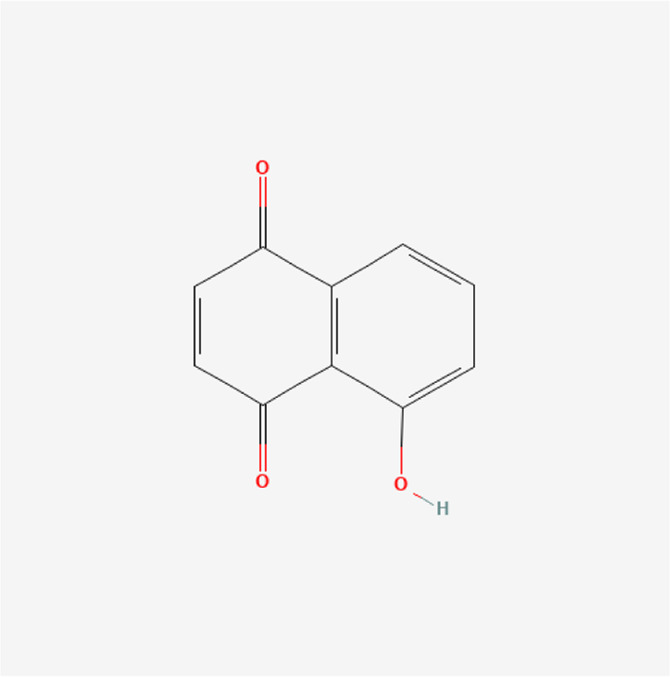	*J. regia L*
			
			
			
Corylifolinin	C_20_H_20_O_4_	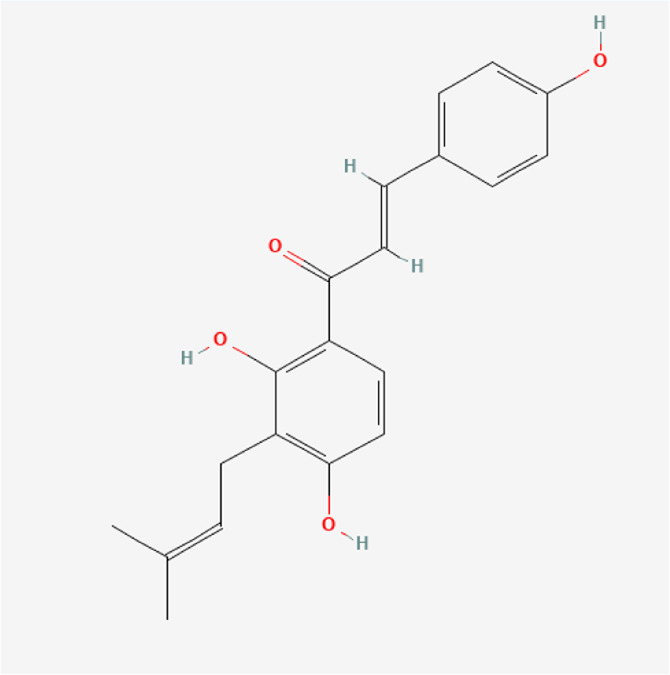	*P. corylifolia L.*
Bavachin	C_20_H_20_O_4_	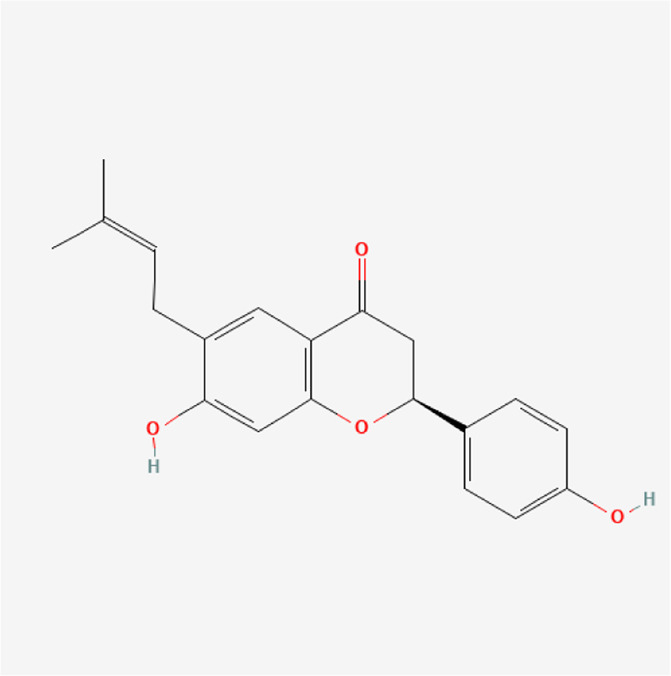	*P. corylifolia L.*
			
			
			
Bakuchiol	C_18_H_24_O	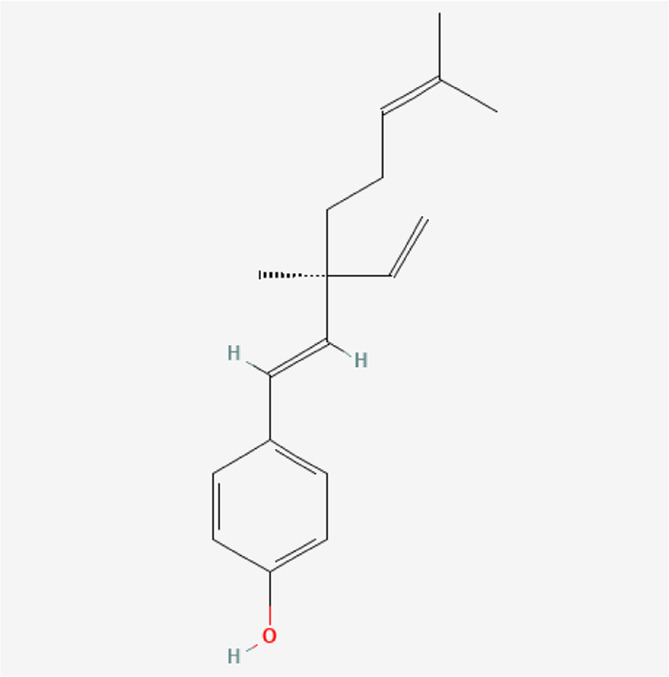	*P. corylifolia L.*
			
			
			
Isobavachin	C_20_H_20_O_4_	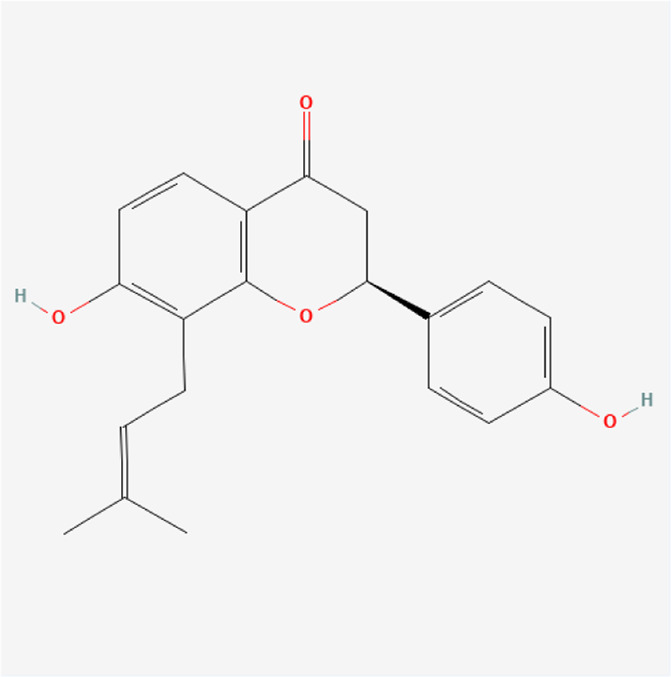	*P. corylifolia L.*
Bavachalcone	C_20_H_20_O_4_	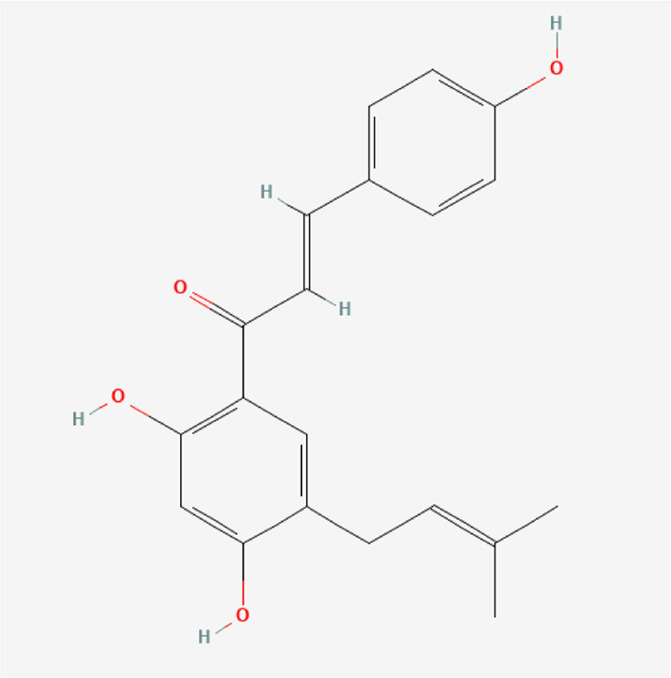	*P. corylifolia L.*
			
			
			
Stigmasterol	C_29_H_48_O	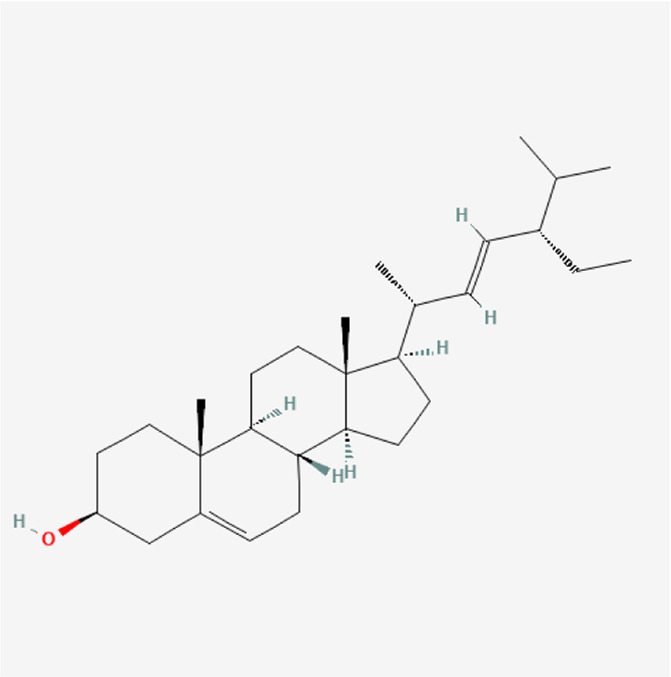	*P. corylifolia L.*
			
			
			
Xanthotoxin	C_12_H_8_O_4_	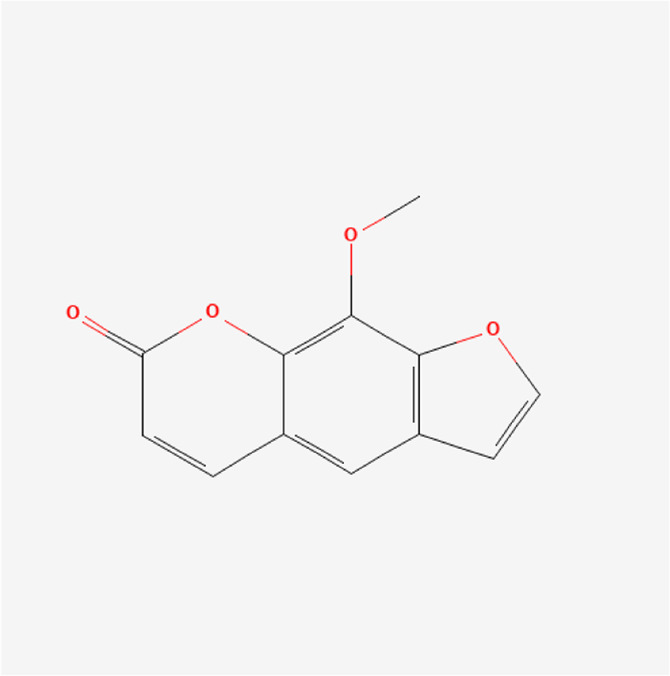	*P. corylifolia L.*
Backuchiol	C_18_H_24_O	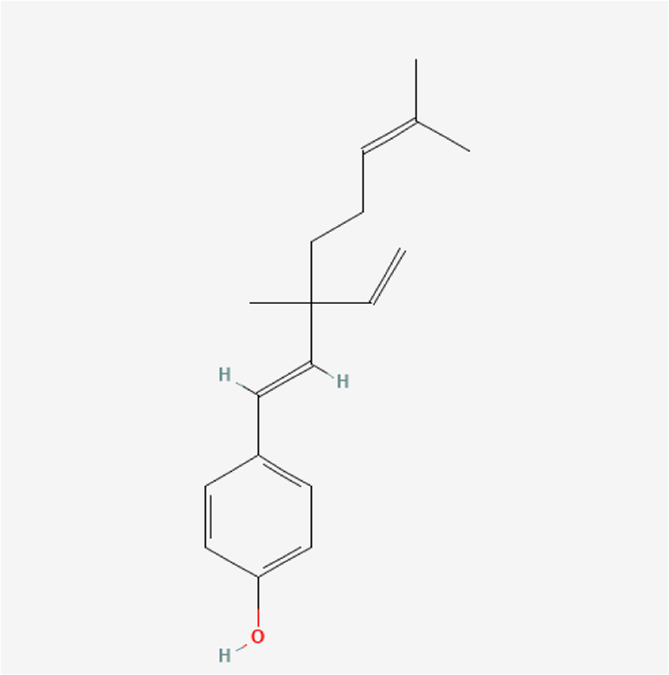	*P. corylifolia L.*
			
			
			
Angelicin	C_11_H_6_O_3_	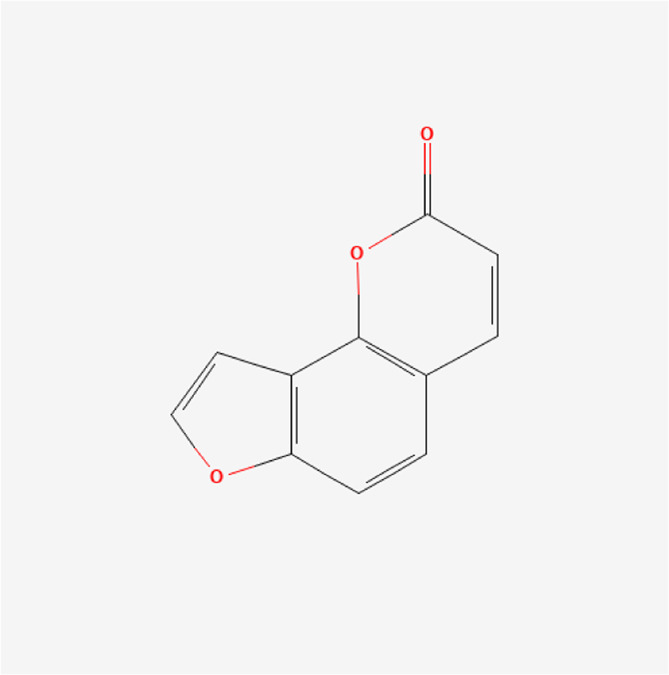	*P. corylifolia L.*
			
			
			
Isobavachalcone	C_20_H_20_O_4_	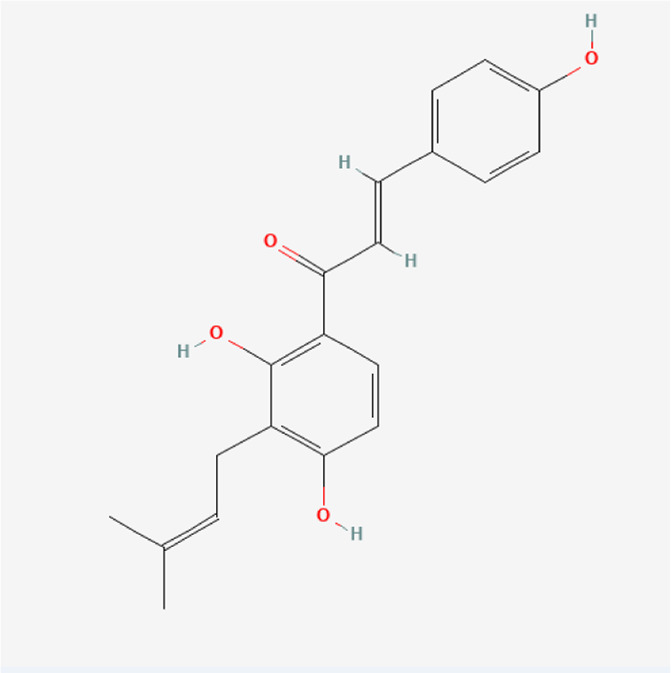	*P. corylifolia L.*
			
			
			

### GO/KEGG Enrichment Analysis

**FIGURE 1:**
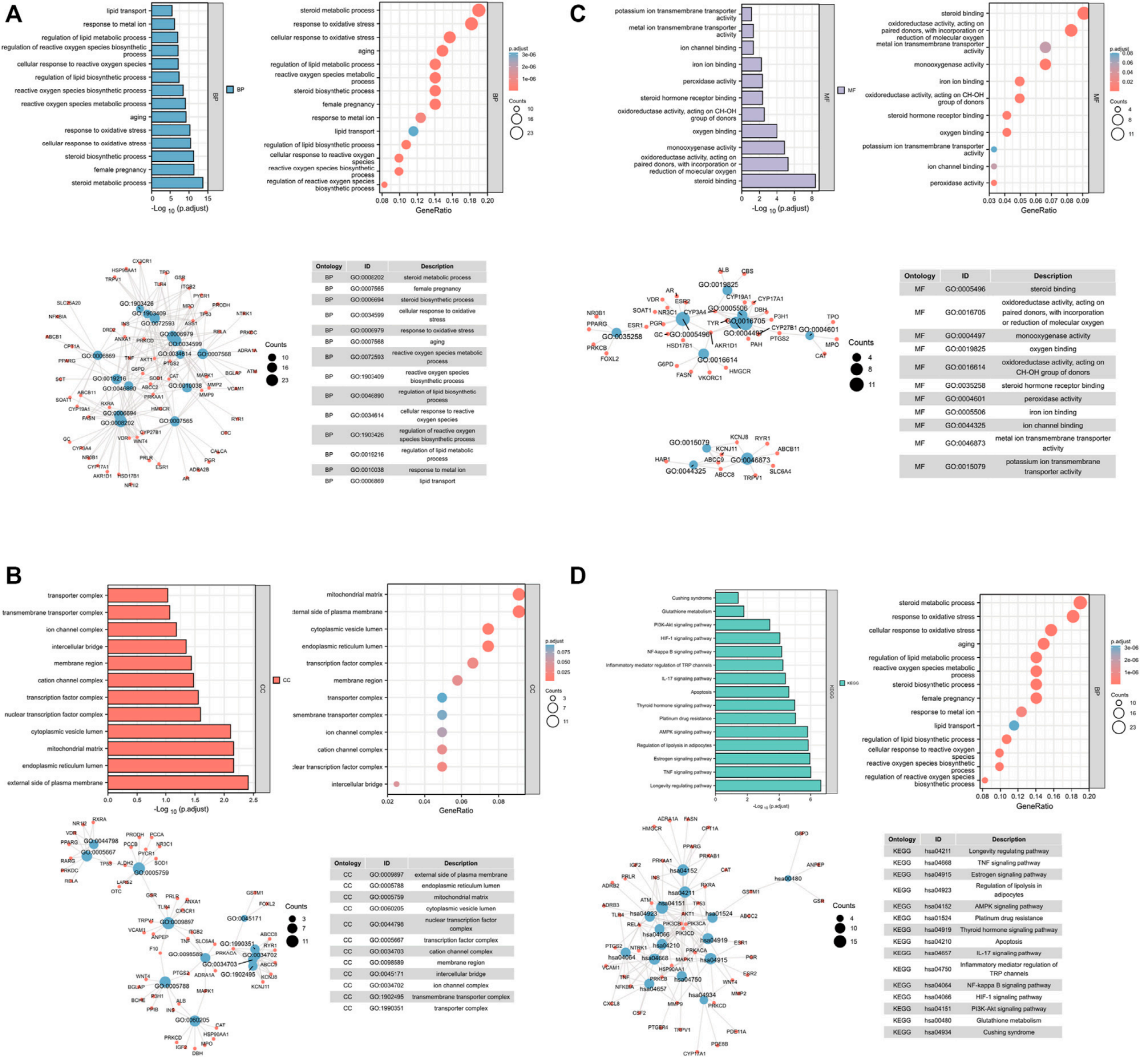
Analysis of common target genes by GO and KEGG. **(A)** BP. **(B)** CC. **(C)** MF. **(D)** KEGG. Left-up panel shows the *p*-value of current items, the right-up shows the gene ratio of current items, the left-bottom shows the interaction between current items, and the right-bottom shows the detailed list of current items.

To gain further insights into the multifunction of potential therapeutic targets for OP, we performed functional enrichment analysis based on biological process GO terms and KEGG pathways (Gu et al., 2021) by using the 121 common targets. A total of 1,536 items (Supplementary Table S4) of biological processes (BPs) (Figure 1A), 50 items of cellular components (CCs) (Figure 1B), and 137 items of molecular function (MF) (Figure 1C) were obtained based on *p*-value (*p* < 0.05) (Figure 1; Supplementary Table S4).

The targets after sorting were mainly concentrated in oxidative stress, iron metabolism, and lipid synthesis. About 157 signaling pathways were screened by KEGG pathway enrichment analysis (Figure 1D), and the most prominent ones were oxidative stress, iron metabolism, and lipid metabolism. Previous reports indicated that oxidative stress, ion metabolism, and lipid metabolism played an important role in ferroptosis (Luo et al., 2018; Fujiki et al., 2019). In addition, OP is accompanied with ferroptosis (Lu et al., 2019; Ma et al., 2020). Thus, we considered that QEP may simultaneously affect ferroptosis and bone formation in OP.

### Phytochemical Analysis of the QEP Extract by HPLC

**FIGURE 2:**
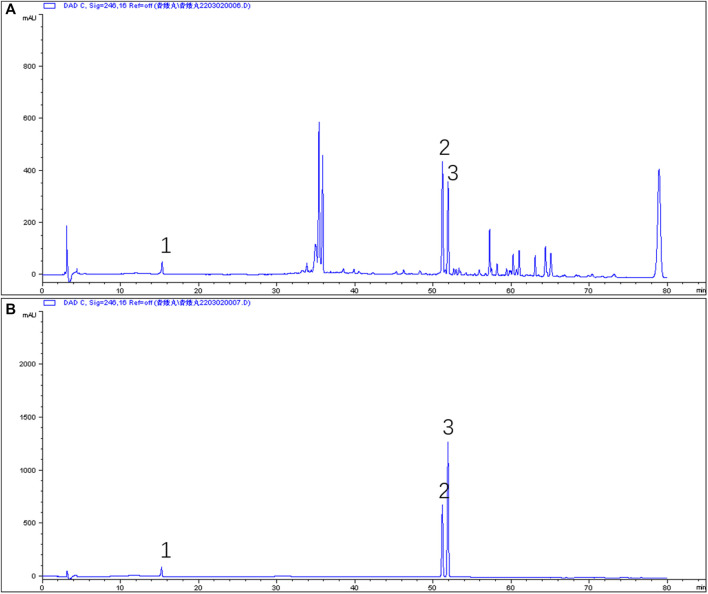
**(A)** HPLC of QEP **(B)** HPLC of geniposide, psoralen, and isopsoralen (1, geniposide; 2, psoralen; 3, isopsoralen).

The effective components of the QEP extract were analyzed through HPLC. According to the retention time and UV spectrum of HPLC, approximately 14 chromatographic peaks were identified as the phytochemical profile of QEP (Figure 2A). The chromatographic peaks 1, 2, and 3 represent geniposide, psoralen, and isopsoralen, respectively (Figure 2B). This result is in accordance with those from another study (Lu et al., 2018).

### QEP Promotes Osteogenesis and Inhibits Ferroptosis

**FIGURE 3:**
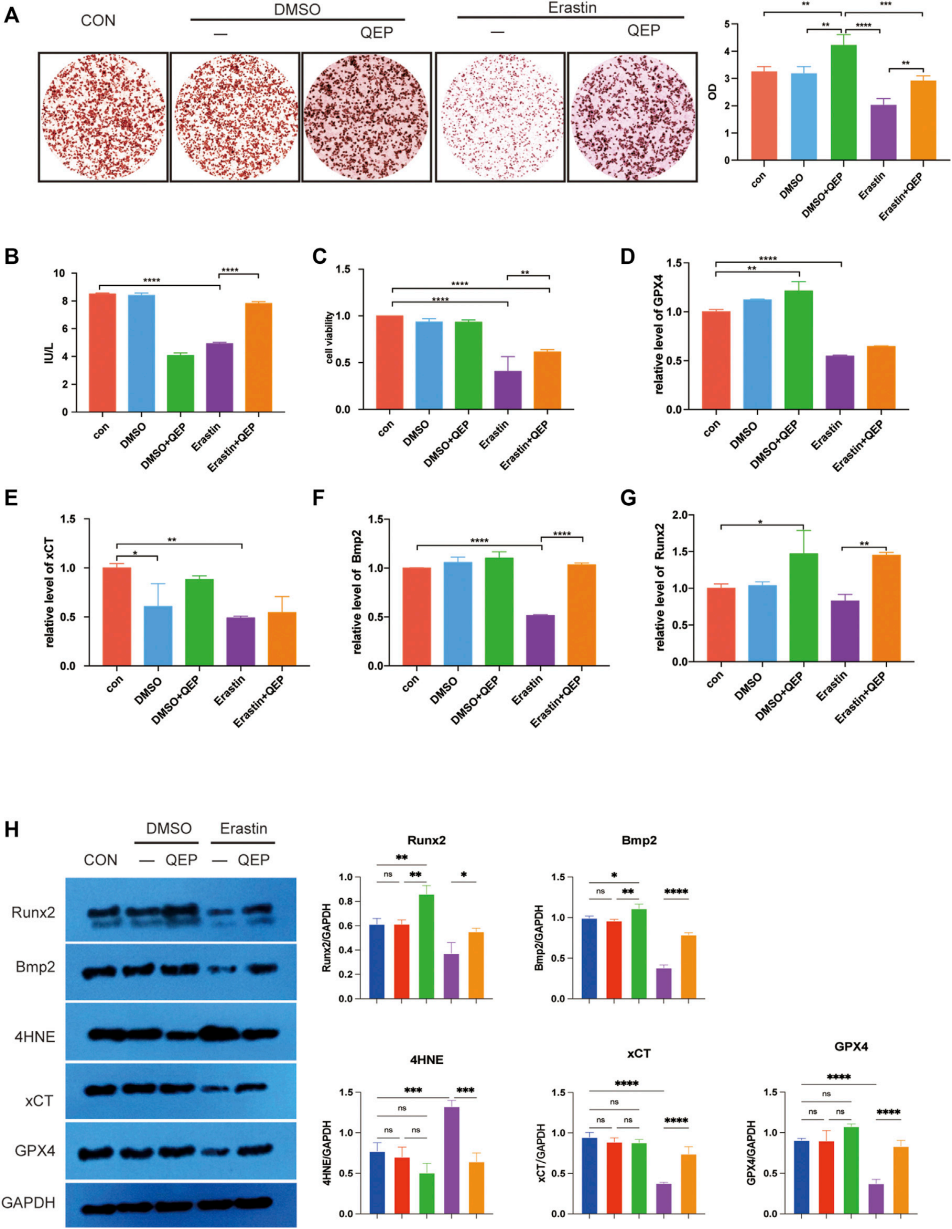
Effect of QEP on osteogenic induction and ferroptosis in hFOB 1.19 cells. **(A)** Alizarin red S staining. The formation of mineralised nodules was observed through staining. **(B)** Comparison of ALP levels. ALP was detected by ELISA. **(C)** Cell viability was detected by CCK8 assay. **(D–G)** Relative mRNA expression of genes related to osteogenesis and ferroptosis in different groups. **(H)** Western blot analyses were performed to detect the expression of osteogenesis- and ferroptosis-related proteins (Qing: QEP; *p < 0.05, **p < 0.01, ***p < 0.001, and ****p < 0.0001).

The aforementioned results were based on network analysis. We further investigated the effect of QEP on osteogenesis and ferroptosis. ARS staining was performed to observe the mineralized nodules of hFOB 1.19 cells in different groups. As shown in Figure 3A, mineralized nodules were highly produced in QEP-treated cells. As predicted, erastin pretreatment decreased the osteogenesis of hFOB 1.19 cells, but this trend was rescued by QEP. The alizarin red quantification also showed similar results. We also performed ELISA to detect ALP, which is a marker of early-stage osteogenesis differentiation. The results supported the remission of QEP on broken osteogenesis differentiation induced by erastin (Figure 3B). In addition, QEP improved cell viability compared with that in the erastin-pretreatment group (Figure 3C). BMP2 can induce osteoblast differentiation, while Runx2 is the osteoblast-specific transcription factor. 4-Hydroxynonenal (4HNE), a well-known marker of ferroptosis, induces ferroptosis, while xCT and GPX4 activities protect cells from ferroptosis. Our data showed that QEP could improve osteogenesis genes at mRNA level in erastin-induced hFOB 1.19 cells (Figures 3D–G). Meanwhile, erastin treatment downregulated the protein level of xCT and GPX4, whereas QEP reversed such an effect (Figure 3H). Evidence demonstrated that QEP promotes osteogenesis and inhibits ferroptosis.

### QEP Inhibits Ferroptosis by Downregulating ATM

**FIGURE 4:**
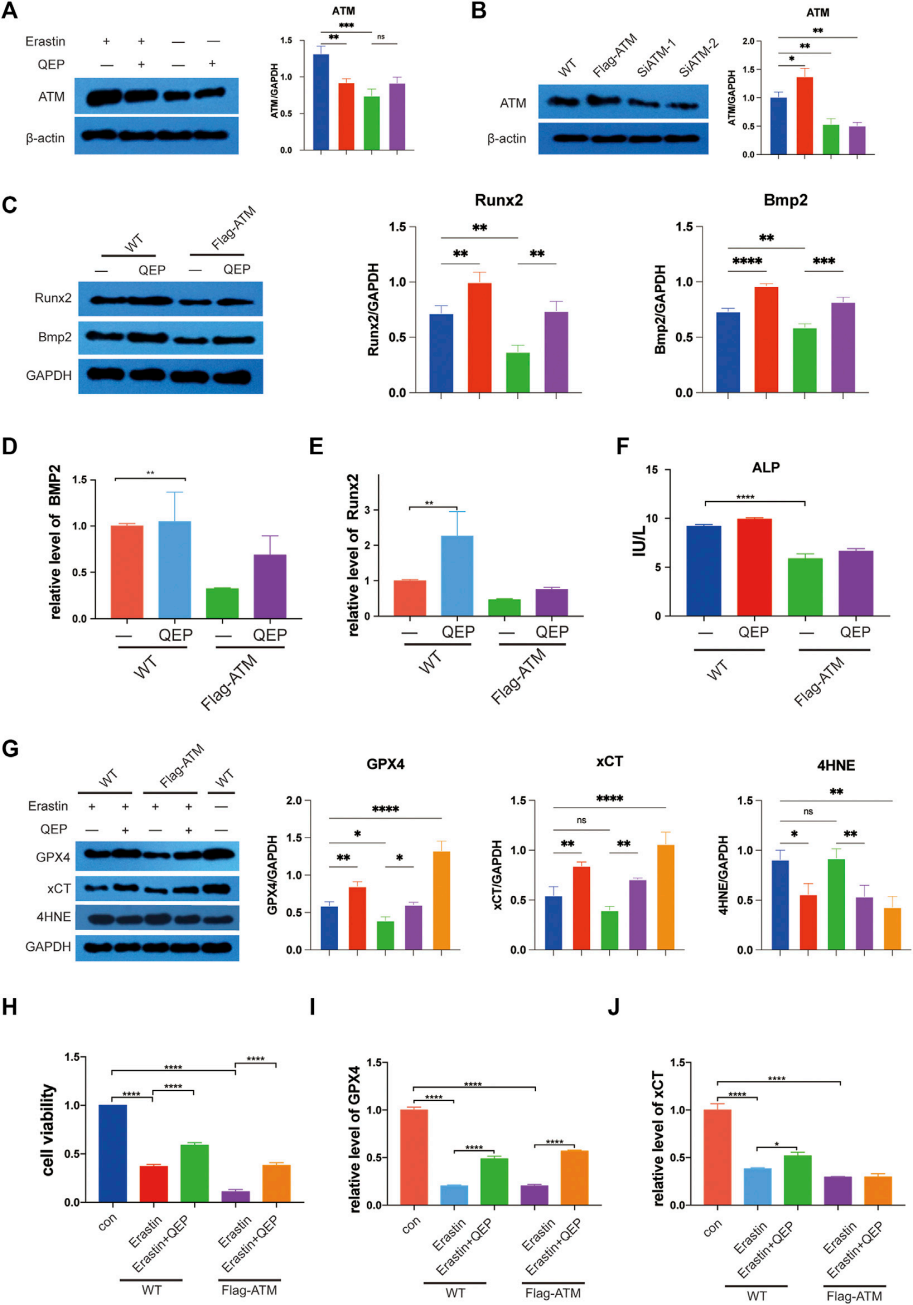
QEP inhibits the ATM expression and ferroptosis. **(A)** Western blot showed that QEP inhibits ATM. **(B)** Establishment of ATM overexpressing and ATM knockdown hFOB 1.19 cells. **(C)** QEP enhanced osteogenesis by downregulating ATM. **(D–F)** Relative mRNA expression of osteogenesis-related genes and ferroptosis-related genes in different groups. **(F)** Overexpressing ATM decreased ALP in hFOB 1.19 cells. **(G)** Overexpressing ATM deteriorated ferroptosis induced by erastin, but QEP inhibited it. **(H)** QEP improved cell viability. **(I,J)** QEP enhanced the mRNA level of ferroptosis-related genes (Qing: QEP; *p < 0.05, **p < 0.01, ***p < 0.001, and ****p < 0.0001).

First, we intersected previous drug targets and disease targets and obtained 121 drug–disease targets. A protein interaction network was constructed in the STRING database to import the protein interaction data into Cytoscape. Maximal clique centrality (MCC) was calculated by the cytoHubba plug-in pair interaction network, and the top 30 genes with the highest scores were extracted (Supplementary Table S5). Genes related to ferroptosis obtained from FerrDB were crossed, and five crucial genes were obtained: *ATM*, *PIK3CA*, *MAPK1*, *TLR4*, and *TP53*. According to previous studies, ATM was located in the peroxisome from Reactome, and one of the GO entries for ATM, GO: 0005782, also indicated that ATM was positioned in the peroxisomal matrix. Based on the hints of bioinformatics analysis, we focused on ATM, which is an essential factor for ferroptosis, and inhibits the expression of GPX4 (Chen et al., 2020b). We investigated whether QEP inhibits ferroptosis *via* ATM. Erastin treatment promoted the protein expression of ATM, but QEP relieved the effect (Figure 4A, lanes 3 and 4). We then constructed the ATM overexpressing and ATM knockdown hFOB 1.19 cell model (Figure 4B). In the overexpressing ATM cell model, QEP still had a positive effect on osteogenesis, as verified by the Western blot results (Figure 4C). Meanwhile, QEP upregulated the transcription of osteogenesis-related genes. We also collected the supernatant of cultured cells and measured alkaline phosphatase (ALP), which is a primary marker in the initial phase of osteogenesis, as determined by ELISA (Figures 4D–F).

GPX4 and xCT, which are suppressive upstream molecules, were downregulated in the cells under the erastin condition. To some extent, QEP rescued the decreasing protein levels of xCT and GPX4 caused by erastin. Consistent with this finding, 4HNE was upregulated in erastin-stimulated hFOB 1.19 cells, and its level was reduced by QEP (Figure 4G). The results indicated that erastin induced a highly abundant ATM, so we investigated whether the overexpression ATM has a positive effect on ferroptosis in Flag-ATM hFOB 1.19 cells and whether QEP rescues ferroptosis caused by the overexpression of ATM. A previous study reported that QEP upregulated the transcription of ferroptosis-related genes (Zhou and Bao, 2020). QEP significantly enhanced cell viability induced by erastin in WT or Flag-ATM hFOB 1.19 cells (Figure 4H). Figures 4I,J demonstrated that QEP affected the transcription of ferroptosis-related genes but did not rescue the expression of xCT in Flag-ATM hFOB 1.19 cells. The present results showed that QEP had a negative effect on ferroptosis induced by erastin by downregulating ATM translation and had a protective effect on osteogenesis.

### QEP Inhibits Ferroptosis by Activating the AKT/PI3K Pathway

**FIGURE 5:**
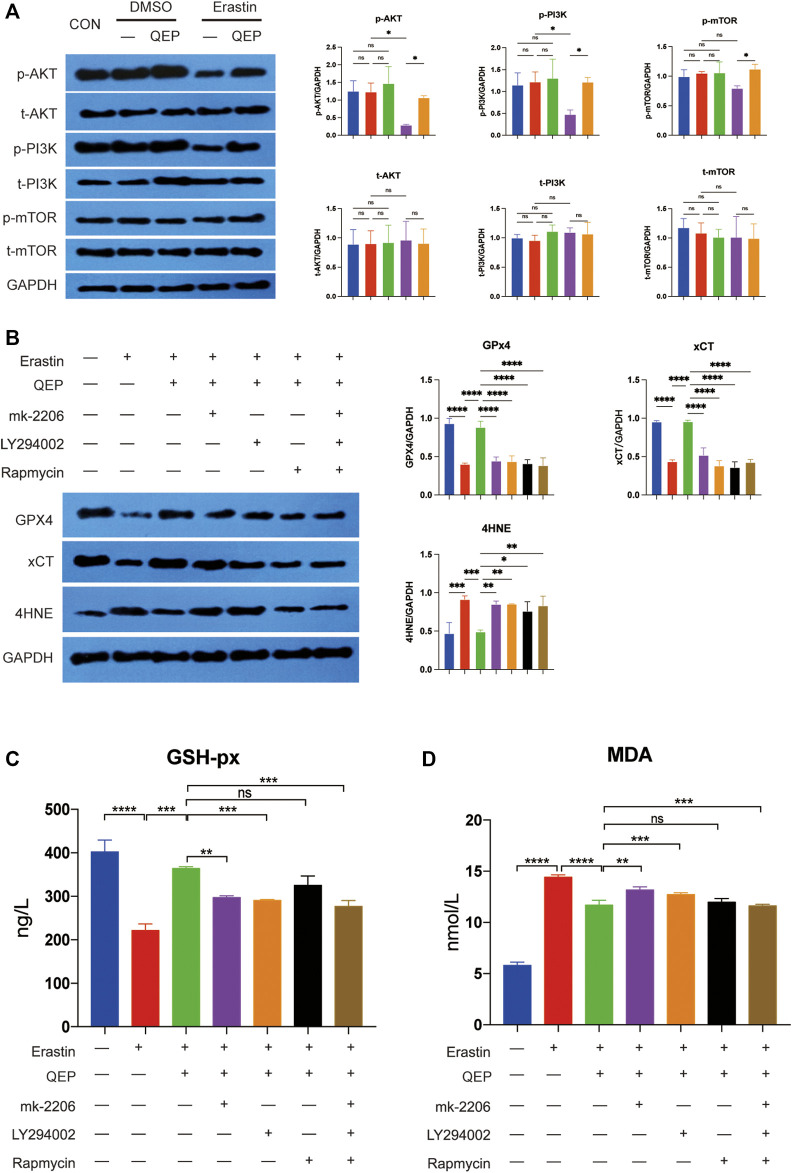
QEP inhibits ferroptosis by activating the AKT/PI3K pathway. **(A)** AKT/PI3K pathway activity is promoted by QEP, even under erastin conditions. t-AKT, t-PI3K, and t-mTOR mean total protein of AKT, PI3K, mTOR. p-AKT, p-PI3K, and p-mTOR mean the phosphorylation of AKT, PI3K, and mTOR. **(B)** Blockade of AKT/PI3K pathways aggravated ferroptosis. **(C,D)** QEP reduced glutathione peroxidase (GSH-Px) and malondialdehyde (MDA). (Qing: Qing`e pill; t: total; p: phosphorylation, *p < 0.05, **p < 0.01, ***p < 0.001, and ****p < 0.0001).

According to the results of bioinformatic analysis, we investigated whether QEP inhibits ferroptosis through the AKT/PI3K pathway (Zhou et al., 2019). Erastin reduced the proliferation of hFOB 1.19 cells (Figure 4H), and the key genes of the AKT/PI3K pathway were inhibited by erastin. However, the situation was reversed by treatment with QEP (Figure 5A). The result confirmed again that QEP ameliorated ferroptosis, but MK-2206 (AKT inhibitor), LY294002 (PI3K inhibitor), or rapamycin (mTOR inhibitor) blocked the amelioration of QEP (Figure 5B). In addition, QEP seemed to reduce oxidative stress caused by erastin, but blockers of the AKT/PI3K pathway weakened this effect (Figures 5C,D). These data indicated that QEP could inhibit ferroptosis through the AKT/PI3K pathway.

### QEP Improves Osteoporosis and Inhibits Ferroptosis in OVX Rats

**FIGURE 6:**
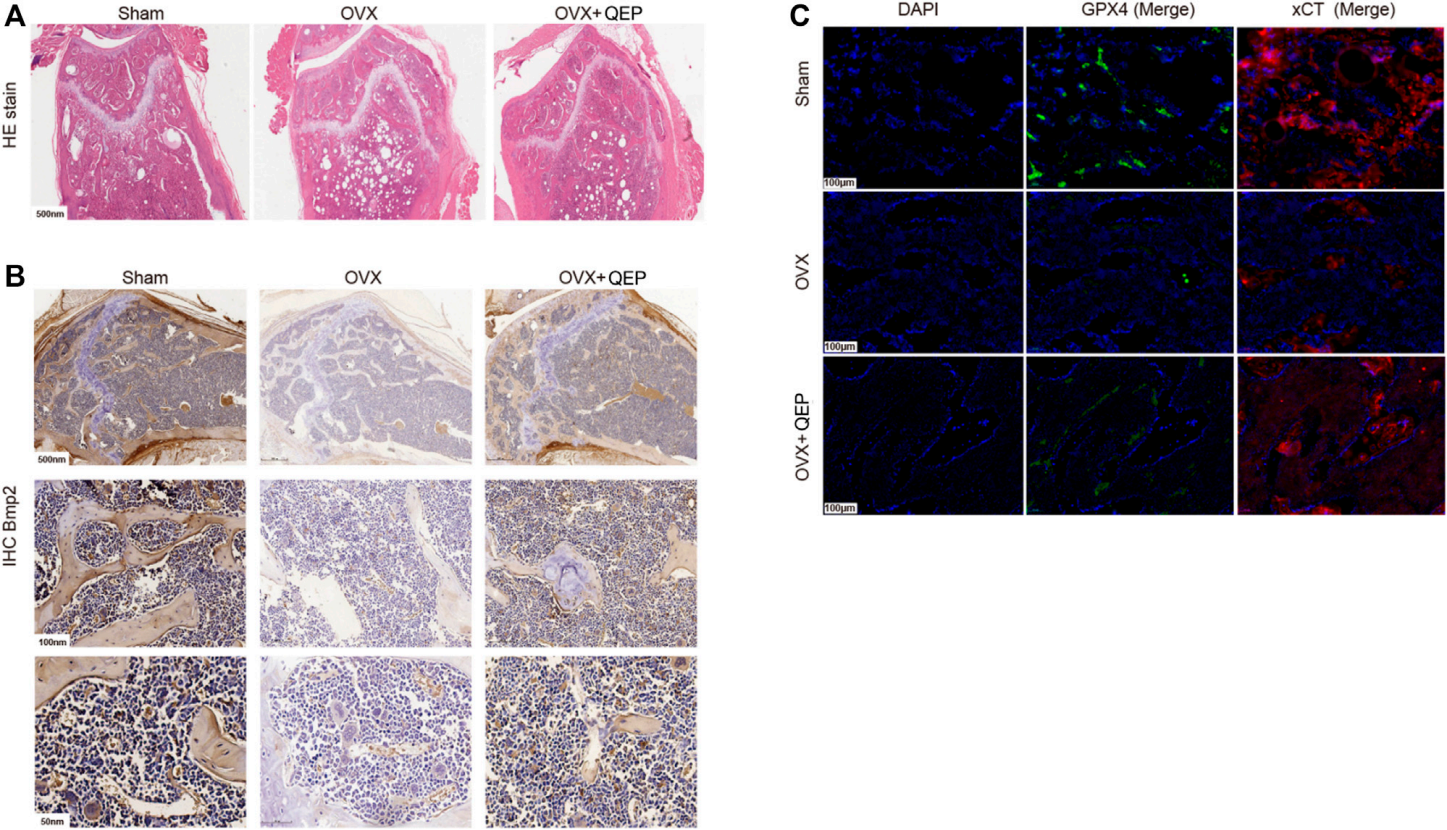
QEP improved osteoporosis in OVX rats. **(A)** HE-stained sections of the tibia from different groups. **(B)** Tibia sections stained with Bmp2 for IHC analysis. **(C)** Tibia sections were stained with GPX4 and xCT for IF analysis (Sham, sham-operated group; OVX, ophorectomy group; OVX + Qing, treated with QEP in OVX rats).

**FIGURE 7:**
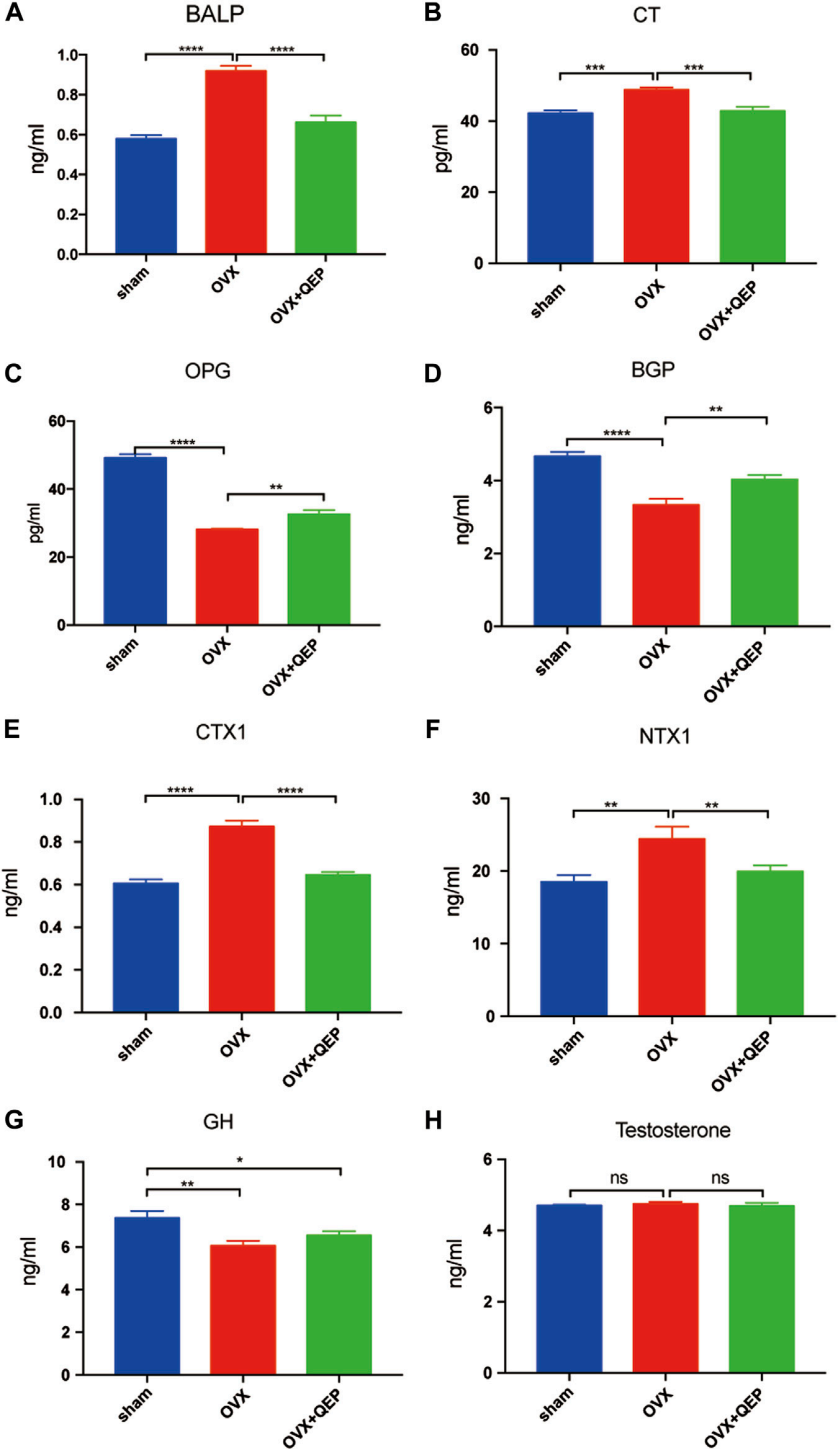
The effect of QEP on serum osteoporosis indicators from rats. **(A)** BALP, bone alkaline phosphatase; **(B)** CT, calcitonin; **(C)** OPG, osteoprotegerin; **(D)** BGP, osteocalcin; **(E)** CTX-1, cross-linked C-telopeptide of type I collagen; **(F)** NTX-1, cross-linked N-telopeptide of type I collagen; **(G)** GH, growth hormone; **(H)** Testosterone. *p < 0.05, **p < 0.01, ***p < 0.005, and ****p < 0.001. ns *p* > 0.05, *n* = 3).

As shown in Figure 6A, 4 weeks after the OVX surgery, the trabecular bones appeared thinner, several bone lacunas became vacant, trabecular spaces widened, and the vessel density decreased in OVX rats. The bone trabecula arrangement was improved in rats treated with OVX + QEP by increasing the number of trabecula and trabecular connections. Additionally, Bmp2 was reduced in OVX rats compared with that in sham rats, but those treated with QEP showed a slight recovery (Figure 6B). On the other hand, OVX rats showed a decline in negative regulators of ferroptosis, such as GPX4 and xCT, but QEP administration enhanced their expression (Figure 6C). Serum bone formation markers and bone resorption markers were tested to determine the osteoporotic prevention effect of QEP in an OVX osteoporosis model. Blood was collected for ELISA. The levels of osteoprotegerin (OPG), BGP, and CT were reduced significantly in OVX rats compared with those in the Sham group, and QEP efficiently rescued the effect, that is, even bone BALP showed no change among the groups. These findings demonstrated that osteogenesis in OVX rats was insufficient but was improved by QEP administration (Figures 7A–D). Meanwhile, active osteoclastic bone resorption occurred in OVX rats, given that cross-linked NTX-1 and cross-linked CTX-1 were significantly increased in OVX rats. QEP administration improved the situation (Figures 7E,F) by increasing the level of growth hormone (GH), which was decreased in OVX rats, although no obvious change in testosterone was detected (Figures 7G,H). Overall, QEP had a positive effect on calcium-phosphorus metabolism and osteogenesis in OVX rats and also prevented osteoclastogenesis.

## Discussion

QEP is a traditional Chinese medicine formula that consists of four ingredients. Recent *in vivo* and *in vitro* findings suggest that TCM may provide potential therapeutic benefits for the treatment of osteoporosis (He et al., 2019). The chemical compounds in QEP have certain pharmacological activities (Hou et al., 2020). To some extent, researchers have verified the therapeutic efficacy of QEP on OP (Zhang et al., 2016). To better understand the potential pharmacological mechanism, we used network analysis and bioinformatic analysis to determine the mechanism through which QEP prevents and controls OP. Network analysis was used to predict drug targets and possible molecular mechanisms involved and identify pathogenic mechanisms of OP and elucidate the mechanism of action. Here, we presented a series of common targets, of which the most prominent ones are related to the AKT/PI3K pathway.

Network analysis is a rapidly emerging approach based on network models and systems biology (Guo et al., 2019; Luo et al., 2020). In the present study, network analysis was utilized to investigate the mechanism of action of QEP against osteoporosis. Two most cited TCM online databases (TCMSP and BATMAN) were employed for further analysis. We searched the four botanical drugs of QEP in the databases to obtain the chemical compounds and the potential target genes. Targets of OP were obtained through GeneCards and DisGeNET. The intersection showed the common targets between QEP and OP. This specific intrinsic link between common targets may be crucial to their role in the effect of QEP on OP. GO, KEGG, and protein–protein interaction (PPI) network analyses showed that the common target genes focused on oxidative stress, iron metabolism, and lipid synthesis, which were closely correlated with ferroptosis. Thus, our study focused on determining the underlying targets related to osteoporosis, ferroptosis, and QEP. Among the five genes with the highest scores, ATM, as a biomarker of ferroptosis (Chen et al., 2020a), is a crucial pathway among common targets, and four out of five were closely related to the PI3K pathway. Thus, we focused on the PI3K pathway to determine the underlying mechanism of QEP in improving osteoporosis.

Many studies have pointed out that ferroptosis often occurs with osteoporosis (Lu et al., 2019; Ma et al., 2020). We used hFOB 1.19 cells to determine the effect of QEP on osteogenic induction and ferroptosis. All the data demonstrated that QEP has a positive influence on osteogenic induction of hFOB 1.19 cells and a negative influence on ferroptosis. According to previous research, ATM is a critical factor of ferroptosis, while inhibition of ATM expression significantly suppresses it (Maciejczyk et al., 2019; Chen et al., 2020b). Our data showed that QEP inhibited ATM under erastin conditions; hence, QEP might inhibit ferroptosis *via* ATM. The anti-ferroptosis effect was shown as the upregulation of xCT and GPX4 and the downregulation of 4HNE. It might mainly affect protein translation and stability as the mRNAs of xCT and GPX4 were not significantly affected.

Considering the common targets, the PI3K pathway is related to ferroptosis, cell proliferation, and QEP (Wang et al., 2020; Yi et al., 2020; Zhao Y. et al., 2021). The PI3K pathway is of great importance in cell proliferation, and activating it could protect cells from ferroptosis (Chen et al., 2020a; Yi et al., 2020; Sun et al., 2022). Thus, we detected the effect of QEP on the AKT/PI3K pathway. The results showed that QEP promoted the AKT/PI3K pathway, and the blockade of AKT prevented this effect. QEP improved the transcription of the key proteins to induce osteogenic differentiation, such as Bmp2 and Runx2 (Almalki and Agrawal, 2016). Thus, QEP promoted osteogenic induction and inhibited ferroptosis by promoting the AKT/PI3K pathway and inhibiting ATM. This finding is consistent with the experiments *in vivo*. Our data *in vivo* demonstrated that QEP favors osteogenesis by promoting the osteogenic protein expression and calcium-phosphate metabolism and inhibiting osteoclast function. QEP improved the erastin-induced ferroptosis in the tibia in OVX rats as the related proteins were accordingly influenced.

Chinese traditional medicine has shown its advantages in the treatment of OP. However, considering the multi-target effect of TCM (Wong et al., 2020), multiple mechanisms for anti-osteoporosis are involved. The most general mechanisms contributing to anti-osteoporosis are estrogen-like effects. In previous studies, many TCMs have this effect, including *Rhizoma drynaria* (Zhou et al., 2022), QingYan formula (Zhao F. et al., 2021), and Er-xian decoction (Wong et al., 2021). QEP is already widely used in the clinic which also has estrogen-like activity (Xiong et al., 2022). GU SHU KANG, a TCM formula, has beneficial roles on bone tissue *via* regulating calcium balance and Vitamin D metabolism (Li et al., 2019). Icariin, the active ingredient of *Epimedium*, increased osteoblastic differentiation by activating the cAMP/PKA/CREB pathway (Chen et al., 2019). According to the previous study, QEP has the effect of increasing β-catenin and BMP2 expression to improve bone health (Li et al., 2017; Shuai et al., 2019). In the present study, we found QEP was shown to prevent ferroptosis of osteoblasts and increase osteogenesis *in vivo* and *in vitro*. This work identified a novel potential therapeutic mechanism of QEP.

In conclusion, this study determined the effect of QEP on osteoporosis and ferroptosis. We here provided reasonable and comprehensive insights into the pharmacological effects of QEP on osteoporosis and ferroptosis *via* the network analysis technique. We further performed a series of experiments and demonstrated that QEP improved OP and inhibited ferroptosis *via* ATM and the AKT/PI3K pathway. Finally, this work provided a basis for using QEP for the treatment of OP from the multi-perspective of network analysis and experimental verification.

## Data Availability

The datasets presented in this study can be found in online repositories. The names of the repository/repositories and accession number(s) can be found in the article/Supplementary Material.

## Supplementary Material

Supplementary Figure S1 Potential molecular mechanisms and functional enrichment analysis of QEP-OP common targets. **(B)** Functional enrichment Analysis based on 121 common target- proteins. **(C)** The network of different GO items. **(D)** The PPI network of 121 targets-related genes.

## Abbreviations

ATM, ATM serine/threonine kinase; AKT, AKT serine/threonine kinase; BMP2, bone morphogenetic protein 2; GPX4, glutathione peroxidase 4; OP, osteoporosis; OVX, ovariectomy; PI3K, phosphoinositide-3-kinase; QEP, Qing’e pill; ROS, reactive oxygen species; Runx2, RUNX family transcription factor 2; xCT, solute carrier family 7 member 11; 4HNE, 4-hydoxynonenal.
