# Supplementary material for: Qing`e Pill Inhibits Osteoblast Ferroptosis via ATM Serine/Threonine Kinase (ATM) and the PI3K/AKT Pathway in Primary Osteoporosis
Source: Front Pharmacol. 2022 Jul 5;13:902102. doi: 10.3389/fphar.2022.902102 (PMC9294279; doi:10.3389/fphar.2022.902102)
Supplement: Supplementary file 1 [file Table1.docx]

**Supplement Table 1** Target genes of QEP

| Element | Target gene | Element | Target gene | Element | Target gene |
| --- | --- | --- | --- | --- | --- |
| *PSORALEA CORYLIFOLIA L.* | ABCB1 | *ALLIUM SATIVUM L.* | ADRB3 | *EUCOMMIA ULMOIDES OLIV.* | PRKCD |
| *PSORALEA CORYLIFOLIA L.* | ABCB11 | *ALLIUM SATIVUM L.* | AKR1D1 | *EUCOMMIA ULMOIDES OLIV.* | PRKDC |
| *PSORALEA CORYLIFOLIA L.* | ABCC2 | *ALLIUM SATIVUM L.* | ALDH1A1 | *EUCOMMIA ULMOIDES OLIV.* | PRLR |
| *PSORALEA CORYLIFOLIA L.* | ABCC8 | *ALLIUM SATIVUM L.* | ALDH2 | *EUCOMMIA ULMOIDES OLIV.* | PRODH |
| *PSORALEA CORYLIFOLIA L.* | ABCC9 | *ALLIUM SATIVUM L.* | ANXA1 | *EUCOMMIA ULMOIDES OLIV.* | PYCR1 |
| *PSORALEA CORYLIFOLIA L.* | AKT1 | *ALLIUM SATIVUM L.* | ASL | *EUCOMMIA ULMOIDES OLIV.* | RYR1 |
| *PSORALEA CORYLIFOLIA L.* | ANPEP | *ALLIUM SATIVUM L.* | ASRGL1 | *EUCOMMIA ULMOIDES OLIV.* | VCAM1 |
| *PSORALEA CORYLIFOLIA L.* | ESR2 | *ALLIUM SATIVUM L.* | ASS1 | *EUCOMMIA ULMOIDES OLIV.* | ALB |
| *PSORALEA CORYLIFOLIA L.* | PRKAA1 | *ALLIUM SATIVUM L.* | BCHE | *EUCOMMIA ULMOIDES OLIV.* | ATM |
| *PSORALEA CORYLIFOLIA L.* | SLC25A4 | *ALLIUM SATIVUM L.* | CALCA | *EUCOMMIA ULMOIDES OLIV.* | CX3CR1 |
| *PSORALEA CORYLIFOLIA L.* | SOAT1 | *ALLIUM SATIVUM L.* | CAT | *EUCOMMIA ULMOIDES OLIV.* | CYP19A1 |
| *ALLIUM SATIVUM L.* | ACHE | *ALLIUM SATIVUM L.* | CBS | *EUCOMMIA ULMOIDES OLIV.* | CYP3A4 |
| *ALLIUM SATIVUM L.* | ADRA1A | *ALLIUM SATIVUM L.* | CBSL | *EUCOMMIA ULMOIDES OLIV.* | FOXL2 |
| *ALLIUM SATIVUM L.* | ADRA1D | *ALLIUM SATIVUM L.* | CPT1A | *JUGLANS REGIA L.* | AR |
| *ALLIUM SATIVUM L.* | ADRA2B | *ALLIUM SATIVUM L.* | CTH | *JUGLANS REGIA L.* | BGLAP |
| *ALLIUM SATIVUM L.* | ADRB2 | *ALLIUM SATIVUM L.* | CYP17A1 | *JUGLANS REGIA L.* | CSF2 |
| *ALLIUM SATIVUM L.* | PAH | *ALLIUM SATIVUM L.* | TPO | *JUGLANS REGIA L.* | CXCL8 |
| *ALLIUM SATIVUM L.* | PCCA | *ALLIUM SATIVUM L.* | TRPV1 | *JUGLANS REGIA L.* | ESR1 |
| *ALLIUM SATIVUM L.* | PCCB | *ALLIUM SATIVUM L.* | TYR | *JUGLANS REGIA L.* | F10 |
| *ALLIUM SATIVUM L.* | PHKG2 | *ALLIUM SATIVUM L.* | VDR | *JUGLANS REGIA L.* | FASN |
| *ALLIUM SATIVUM L.* | PRKAB1 | *ALLIUM SATIVUM L.* | WLS | *JUGLANS REGIA L.* | G6PD |
| *ALLIUM SATIVUM L.* | PRKACA | *ALLIUM SATIVUM L.* | DBH | *JUGLANS REGIA L.* | GGCX |
| *ALLIUM SATIVUM L.* | PTGER4 | *ALLIUM SATIVUM L.* | DRD2 | *JUGLANS REGIA L.* | GSR |
| *ALLIUM SATIVUM L.* | RARG | *ALLIUM SATIVUM L.* | F12 | *JUGLANS REGIA L.* | GSTM1 |
| *ALLIUM SATIVUM L.* | RXRA | *ALLIUM SATIVUM L.* | GC | *JUGLANS REGIA L.* | HSD17B1 |
| *ALLIUM SATIVUM L.* | SCT | *ALLIUM SATIVUM L.* | GPT | *JUGLANS REGIA L.* | HSP90AA1 |
| *ALLIUM SATIVUM L.* | SLC25A20 | *ALLIUM SATIVUM L.* | HAP1 | *JUGLANS REGIA L.* | IGF2 |
| *ALLIUM SATIVUM L.* | SLC6A4 | *EUCOMMIA ULMOIDES OLIV.* | ITGB2 | *JUGLANS REGIA L.* | MMP2 |
| *ALLIUM SATIVUM L.* | SLC7A1 | *EUCOMMIA ULMOIDES OLIV.* | KCNJ8 | *JUGLANS REGIA L.* | MMP9 |
| *ALLIUM SATIVUM L.* | SLC7A8 | *EUCOMMIA ULMOIDES OLIV.* | NR1I2 | *JUGLANS REGIA L.* | NFKBIA |
| *ALLIUM SATIVUM L.* | TLR4 | *EUCOMMIA ULMOIDES OLIV.* | P3H1 | *JUGLANS REGIA L.* | PGR |
| *ALLIUM SATIVUM L.* | TNF | *EUCOMMIA ULMOIDES OLIV.* | WNT4 | *JUGLANS REGIA L.* | PRKCB |
| *ALLIUM SATIVUM L.* | CYP27B1 | *EUCOMMIA ULMOIDES OLIV.* | PDE11A | *JUGLANS REGIA L.* | PTGS2 |
| *ALLIUM SATIVUM L.* | NR3C1 | *EUCOMMIA ULMOIDES OLIV.* | PDE4A | *JUGLANS REGIA L.* | RELA |
| *ALLIUM SATIVUM L.* | NTRK1 | *EUCOMMIA ULMOIDES OLIV.* | PDE5A | *JUGLANS REGIA L.* | SOD1 |
| *ALLIUM SATIVUM L.* | OTC | *EUCOMMIA ULMOIDES OLIV.* | PDE8B | *JUGLANS REGIA L.* | TP53 |
| *ALLIUM SATIVUM L.* | INS | *EUCOMMIA ULMOIDES OLIV.* | PIK3CA | *JUGLANS REGIA L.* | VKORC1 |
| *ALLIUM SATIVUM L.* | KCNJ11 | *EUCOMMIA ULMOIDES OLIV.* | PIK3CB |  |  |
| *ALLIUM SATIVUM L.* | LARS2 | *EUCOMMIA ULMOIDES OLIV.* | PIK3CD |  |  |
| *ALLIUM SATIVUM L.* | MAPK1 | *EUCOMMIA ULMOIDES OLIV.* | PPARG |  |  |
| *ALLIUM SATIVUM L.* | MPO | *EUCOMMIA ULMOIDES OLIV.* | PPIB |  |  |
| *ALLIUM SATIVUM L.* | NR0B1 | *EUCOMMIA ULMOIDES OLIV.* | HMGCR |  |  |
