# Supplementary figures and images for: Qing`e Pill Inhibits Osteoblast Ferroptosis via ATM Serine/Threonine Kinase (ATM) and the PI3K/AKT Pathway in Primary Osteoporosis
Source: Front Pharmacol. 2022 Jul 5;13:902102. doi: 10.3389/fphar.2022.902102 (PMC9294279; doi:10.3389/fphar.2022.902102)

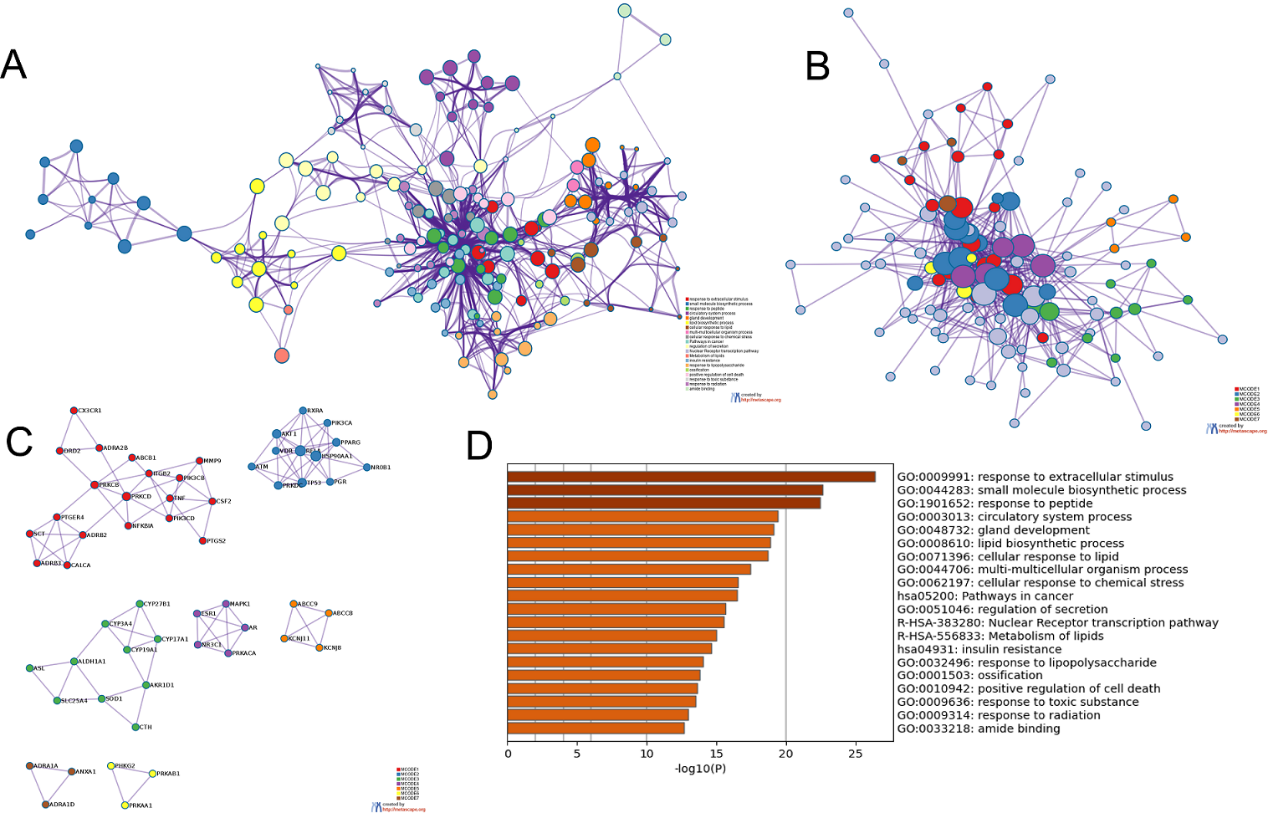

Supplement: Supplementary file 2 [file Image1.tif]
