# Supplementary material for: Qing`e Pill Inhibits Osteoblast Ferroptosis via ATM Serine/Threonine Kinase (ATM) and the PI3K/AKT Pathway in Primary Osteoporosis
Source: Front Pharmacol. 2022 Jul 5;13:902102. doi: 10.3389/fphar.2022.902102 (PMC9294279; doi:10.3389/fphar.2022.902102)
Supplement: Supplementary file 3 [file Table2.docx]

**Supplement Table 2** Targets of QEP and Osteoporosis

| Qing`e Pill | Osteoporosis |
| --- | --- |
| ABAT | DKK1 |
| ACADSB | GPNMB |
| ACSL3 | LRP6 |
| ACSL4 | JUN |
| ACY1 | MIR19B1 |
| ACY3 | PYY |
| ADA | DMD |
| ADAM8 | BEST1 |
| ADORA1 | ACE |
| ADORA2A | PDLIM4 |
| ADORA2B | MIR27A |
| ADRA1B | ATRNL1 |
| ADRA2A | MIR708 |
| ADRA2C | PTH |
| ADSS | MAPK14 |
| ADSSL1 | PLIN4 |
| ALDH1A2 | MIR30B |
| ALDH1A3 | CTSK |
| ALDH3A1 | TNFRSF11B |
| ALDH5A1 | WNK1 |
| AMPD3 | TNFSF11 |
| any | ATIC |
| APLP1 | SHBG |
| ASNS | HJV |
| ASPA | AGT |
| ASPH | AEBP1 |
| ATP1A1 | COL1A1 |
| BCAT1 | CCL20 |
| CACNA1C | ELAVL2 |
| CACNA1D | IDH2 |
| CACNA1S | CYP11A1 |
| CACNB1 | PDLIM3 |
| CACNG1 | MMP1 |
| CACNG2 | PTHLH |
| CAD | IL6 |
| CBR4 | CSF1R |
| CHRFAM7A | SMARCA1 |
| CHRNA1 | PHF20 |
| CHRNA10 | SLPI |
| CHRNA2 | BRCC3 |
| CHRNA3 | RBP4 |
| CHRNA4 | NPNT |
| CHRNA5 | BANF1 |
| CHRNA6 | NM |
| CHRNA7 | GLP1R |
| CHRNA9 | SOST |
| CHRNB1 | TGFB1 |
| CHRNB2 | OXCT1 |
| CHRNB3 | ADCY5 |
| CHRNB4 | SIRT1 |
| CHRND | ITGB1 |
| CHRNE | LRP5 |
| CHRNG | CA2 |
| COL27A1 | NOTCH2 |
| COLQ | CCL27 |
| compound | ST3GAL4 |
| CRYZ | FGF23 |
| CYP51A1 | PLS3 |
| DARS | NAMPT |
| DARS2 | XYLT2 |
| DCT | UBE2K |
| DHRS3 | UGT2B17 |
| DHRS4 | PON1 |
| DMTN | NGLY1 |
| doesn't | CTNND1 |
| DRD3 | PTK2B |
| ELOVL4 | DLX5 |
| EPRS | TRAP |
| ESRRG | BMP2 |
| FADS1 | GAS5 |
| FADS2 | APOE |
| FFAR1 | TACR3 |
| GABRA1 | MRGPRX4 |
| GABRA2 | MIR214 |
| GABRA3 | CYP11B1 |
| GABRA4 | CD80 |
| GABRA5 | ANXA2 |
| GABRA6 | DEPTOR |
| GABRB1 | HSD17B4 |
| GABRB2 | KAT2A |
| GABRB3 | POMC |
| GABRD | TTR |
| GABRE | GSTT1 |
| GABRG1 | GH1 |
| GABRG3 | MIR4516 |
| GABRP | LETM1 |
| GABRQ | RAPGEF5 |
| GLRA1 | ZBTB40-IT1 |
| GLRA2 | SQOR |
| GLRA3 | NOX4 |
| GOT1 | KL |
| GOT2 | NAT10 |
| GP9 | MBL3P |
| GRIA1 | GLO1 |
| GRIA2 | SMPD1 |
| GRIA3 | BTF3P11 |
| GRIA4 | ALOX15 |
| GRIN3A | MIR203A |
| have | PIK3CG |
| HBA1 | SPARC |
| HBA2 | IGF1 |
| HDAC2 | RMND1 |
| HDAC9 | GPX1 |
| HTR1A | FLT3 |
| HTR1B | LRRK1 |
| HTR1D | KAT2B |
| HTR2A | H4C14 |
| HTR2B | HSPB1 |
| HTR2C | AMD1 |
| HTR3A | TERC |
| HTR3B | TNFAIP8L2 |
| HTR3C | WNK4 |
| HTR3D | RNU1-4 |
| HTR3E | MIR21 |
| IDNK | JUNB |
| ITGAL | SBDS |
| ITPR1 | GTF2H1 |
| ITPR2 | ADIPOQ |
| ITPR3 | MIR29A |
| KCNH2 | GJA4 |
| KCNJ1 | WNT1 |
| KCNJ10 | MIR590 |
| KCNJ12 | H4C1 |
| KCNJ14 | PROK2 |
| KCNJ15 | PTN |
| KCNJ3 | EDNRA |
| KCNJ5 | WRN |
| KCNJ6 | ZNF384 |
| KCNJ9 | ATP7A |
| KCNK1 | BHLHE40 |
| KCNK4 | SERPINA1 |
| KCNK6 | MIR23B |
| KCNMA1 | PRMT1 |
| KCNN4 | MMP13 |
| L3HYPDH | SLC12A3 |
| LRAT | BCL2 |
| LYZ | ALPP |
| MLNR | FDPS |
| NOS1AP | IL6R |
| NPPA | GORAB |
| NQO1 | CCR2 |
| NR3C2 | ACP5 |
| NT5E | UBE2Z |
| OGDH | GALNT3 |
| OPRD1 | MALAT1 |
| OPRK1 | DCAF6 |
| OPRM1 | ABI1 |
| P3H2 | CRTAP |
| P3H3 | FSHR |
| P4HA1 | HBM |
| P4HA2 | ANGPTL7 |
| PAICS | PRL |
| PARS2 | RUNX2 |
| PDE10A | SPP1 |
| PDE1A | ACTB |
| PDE1B | DCAF13 |
| PDE1C | MIR221 |
| PDE2A | MIR23A |
| PDE3A | CDX2 |
| PDE3B | HSPG2 |
| PDE4B | VPS35 |
| PDE4C | LTA |
| PDE4D | WWOX |
| PDE6A | TNFRSF11A |
| PDE6B | COPD |
| PDE6C | NLRP3 |
| PDE7A | HMOX1 |
| PDE7B | CALM1 |
| PDE8A | PMM2 |
| PDE9A | MIR485 |
| PGD | SEM1 |
| PIK3R1 | HLA-B |
| POLA2 | NOD2 |
| PPARD | BMP7 |
| PPIA | ACKR3 |
| PPIC | MIR10B |
| PPIF | SAA1 |
| PPIG | ATP6V1G1 |
| PPIH | IL1RN |
| PRKCA | GOLM1 |
| PROSC | LEP |
| PTGER1 | SYN2 |
| PTGER2 | SPI1 |
| PTGER3 | REN |
| PTGFR | MYOG |
| PTGIR | GRAP2 |
| PTGS1 | CTDP1 |
| PYCR2 | NFATC1 |
| PYCRL | PTCH1 |
| RARA | TPM4 |
| RARB | GDF2 |
| RBP1 | BSCL2 |
| RBP3 | COL1A2 |
| RDH11 | STK24 |
| RDH12 | CALCR |
| RDH13 | BMP4 |
| RDH14 | THSD4 |
| RDH5 | TUG1 |
| RDH8 | MIR543 |
| RETSAT | MAB21L2 |
| RHO | MIR148A |
| RINT1 | ADCY10 |
| RIPK1 | MRGPRX1 |
| RLBP1 | CHAD |
| RNASE1 | WWP1 |
| RS1 | MIR373 |
| RXRB | HPSE |
| RXRG | FKBP10 |
| SCN10A | PIR |
| SCN11A | CASR |
| SCN1A | PTPN11 |
| SCN1B | NCF1 |
| SCN2A | TRPS1 |
| SCN2B | IGFBP5 |
| SCN3A | B3GAT3 |
| SCN3B | PLEKHA1 |
| SCN4A | TIMP1 |
| SCN4B | CA10 |
| SCN5A | IFNG |
| SCN7A | H4C13 |
| SCN8A | TOB1 |
| SCN9A | IL17B |
| SLC1A1 | SOD2 |
| SLC25A12 | AHSA1 |
| SLC25A13 | SIGLEC15 |
| SLC28A3 | ZNF266 |
| SLC29A1 | TCF4 |
| SLC29A2 | ATP4A |
| SLC47A1 | MIR142 |
| SLC6A14 | F2R |
| SLC6A7 | SSTR4 |
| SLC8A1 | GNAS |
| SLCO1B1 | BMP15 |
| SLCO1B3 | IL27 |
| SNW1 | MAP3K1 |
| SRD5A1 | TNFRSF1B |
| SRD5A2 | MRPL49 |
| TACR2 | MAPK3 |
| TARS | CLIP2 |
| TARS2 | SEMA3A |
| This | STOM |
| THNSL1 | ZMPSTE24 |
| TOP2A | LEPR |
| TOP2B | HSD11B2 |
| TRPA1 | NOP10 |
| TRPM8 | SETD2 |
| TRPV3 | LRPPRC |
| TYRP1 | ALOX12 |
| VARS | IRS1 |
| VKORC1L1 | RUNX1T1 |
| YWHAE | EZH2 |
| AARS | CLC |
| AARS2 | GPR151 |
| ACOT8 | P2RX7 |
| ADH1A | VEGFA |
| ADH1B | TRAF4 |
| ADH1C | RTEL1 |
| ADRB1 | GABPA |
| AGXT | PPARA |
| AGXT2 | FABP3 |
| ALAS1 | CYP1A1 |
| ALAS2 | ANGPTL2 |
| AMT | MIR140 |
| ARG1 | MBL2 |
| ARG2 | GPRC6A |
| ARRB2 | SLC34A1 |
| ARRDC3 | ARSA |
| ASNSD1 | MAP2K7 |
| ASPG | CYP27A1 |
| AZIN2 | DRAM1 |
| BAAT | HSD3B7 |
| BACE1 | IL15 |
| BCAT2 | CNR2 |
| CACNA1A | FOXO3 |
| CACNA1B | ANGPT1 |
| CACNA1F | TNFSF15 |
| CACNA1G | CDH23 |
| CACNA1I | CCR5 |
| CACNB2 | OSTM1 |
| CACNB3 | GHR |
| CACNB4 | MIR155 |
| CALCOCO2 | MEPE |
| CARS | HS6ST1 |
| CARS2 | MIR187 |
| CCBL1 | VN1R17P |
| CDO1 | NELL1 |
| CEND1 | COMMD3-BMI1 |
| CES1 | GDF11 |
| CHRM1 | IGF1R |
| CHRM2 | HP |
| CHRM3 | ANKH |
| CHRM4 | MIR34B |
| CHRM5 | IRS2 |
| CPS1 | MIR1297 |
| CPT2 | TRAF6 |
| CRAT | GSTP1 |
| CROT | RCBTB1 |
| CSAD | MIR503 |
| CTPS1 | LOX |
| CTPS2 | RRM2B |
| DLG4 | MCM6 |
| DRD1 | MYDGF |
| DRD4 | MIR8084 |
| DRD5 | GREM2 |
| EPHX2 | ZGLP1 |
| FXN | RXFP2 |
| GAMT | GLS |
| GARS | DPP4 |
| GATM | GNRH1 |
| GCAT | IFITM5 |
| GCLC | IL32 |
| GCLM | GLB1 |
| GCSH | SLC2A1 |
| GLDC | JAK2 |
| GLRB | MIR16-2 |
| GLUL | APEX1 |
| GLYAT | CRX |
| GLYATL1 | POF1B |
| GLYATL2 | SETD7 |
| GNMT | KIT |
| GPR18 | AGPAT2 |
| GPRC5A | MAS1 |
| GPT2 | FTCDNL1 |
| GRIN1 | MIR338 |
| GRIN2A | SMURF1 |
| GRIN2B | CTSB |
| GRIN2C | GCG |
| GRIN2D | STARD3 |
| GRIN3B | DBP |
| GSS | COASY |
| GUCY1B3 | ADIPOR1 |
| HCN3 | CYBB |
| HIF1AN | SMAD6 |
| HLCS | COL7A1 |
| HRH1 | HIF1A |
| HRH2 | IFNB1 |
| HRH4 | WT1 |
| HTR1F | TAC1 |
| HTR6 | FOXA1 |
| HTR7 | TAT |
| IARS | GSN |
| IARS2 | MTHFR |
| ICMT | PTRH1 |
| ISCU | GPR39 |
| IYD | ATXN1 |
| KARS | MMP3 |
| KCNA1 | CST3 |
| KCNA2 | SMAD1 |
| KCNA3 | MIR34A |
| KCNA4 | MIR342 |
| KCNA5 | LAMB3 |
| KCNA6 | RFC2 |
| KCNA7 | SELENOP |
| KCNB1 | TGFB3 |
| KCNB2 | QPCT |
| KCNC1 | LGR6 |
| KCNC2 | FOS |
| KCNC3 | H4C4 |
| KCND1 | CCDC170 |
| KCND2 | TERF2IP |
| KCND3 | COL4A4 |
| KCNQ1 | EIF2AK3 |
| KCNQ2 | MIR154 |
| KCNQ3 | LOC110013312 |
| KYNU | POLDIP2 |
| LARS | SMAD3 |
| LCMT1 | SLC11A2 |
| LCMT2 | IL17A |
| LGSN | ROR2 |
| MGMT | TRIO |
| NARS | DMRT3 |
| NARS2 | BAZ1B |
| NFS1 | ANGPTL4 |
| NTRK2 | HGF |
| OAT | VPS53 |
| OAZ1 | RETN |
| OAZ2 | POLD1 |
| OAZ3 | CDH1 |
| PCK1 | FOXO1 |
| PHYKPL | MIR1270 |
| PIK3R2 | MAP9 |
| PIK3R3 | RPL11 |
| PIPOX | TRPV4 |
| PISD | NOG |
| PPAT | MST1R |
| RAB7A | FGA |
| RARRES1 | JAG1 |
| SHMT1 | CA8 |
| SHMT2 | MIRLET7C |
| SIGMAR1 | KLF10 |
| SLC18A1 | SLC37A4 |
| SLC19A3 | CD40 |
| SLC1A2 | ESRRA |
| SLC1A3 | ATG7 |
| SLC1A4 | SLC41A1 |
| SLC1A5 | AGER |
| SLC22A1 | CAMKK2 |
| SLC22A4 | PLEK |
| SLC22A5 | PERCC1 |
| SLC25A15 | GAPDH |
| SLC25A2 | NFKB1 |
| SLC25A29 | NFE2L2 |
| SLC26A6 | BGN |
| SLC32A1 | EHMT1 |
| SLC36A1 | CPAT1 |
| SLC38A3 | SUFU |
| SLC38A7 | S1PR2 |
| SLC6A2 | CCN6 |
| SLC6A3 | S100A12 |
| SLC6A5 | MIR1292 |
| SLC6A9 | PLIN2 |
| SLC7A2 | STAT1 |
| SLC7A3 | IL1B |
| SLC7A4 | ZBTB40 |
| SORT1 | MAPK8 |
| SQLE | RPS16P8 |
| TDO2 | HLA-DQB1 |
| TFAP2B | FANCC |
| TNNC1 | LPAR2 |
| TNNT2 | NDUFAF1 |
| TYMS | POSTN |
| TYW5 | KDM7A |
| VARS2 | GPC6 |
| VDAC1 | LRP4 |
| VDAC2 | JAK1 |
| VDAC3 | TNFRSF1A |
| WARS | PIEZO1 |
| WARS2 | MSC-AS1 |
| XDH | NR4A1 |
| TNK2 | POLG |
| SOAT2 | KCNH6 |
| NT5C2 | PPARGC1A |
| NPC1L1 | TBC1D8 |
| NAE1 | SGMS2 |
| MTTP | FBLN5 |
| KCNA10 | TM7SF2 |
| CDK15 | CIT |
| ASNA1 | ETFA |
| ARAF | HLA-A |
| APAF1 | KDM1A |
| AMHR2 | SP1 |
| ALK | GPR42 |
| AFG3L2 | INPP4B |
| ADRBK2 | CAV3 |
| ADRBK1 | CSF1 |
| ADCY1 | GPR166P |
| ACVRL1 | SVIL |
| ACVR1B | CIITA |
| ACVR1 | NELFA |
| ACSS2 | DCDC2 |
| ACSS1 | FES |
| ACSL1 | NIPA2 |
| ABL2 | H4C8 |
| ABL1 | SNCA |
| ABCG1 | PKN3 |
| ABCA1 | IFIH1 |
| PROS1 | WIF1 |
| PROZ | LIMK1 |
| NQO2 | DOK6 |
| F7 | LRP1 |
| F9 | BMI1 |
| PROC | MIR25 |
| CDK2 | MIR579 |
| CDKN1A | TPH1 |
| GSTM2 | SLCO6A1 |
| GSTA2 | S100P |
| MAOB | MIR618 |
| NCOA2 | GPATCH1 |
| PRSS1 | IFIT2 |
| DUOX2 | ATHS |
| IL5 | ARTN |
| MCAT | CLCN7 |
| ABCB1 | ACAD8 |
| ACHE | SIRT3 |
| ADRA1A | MIR320A |
| ADRA1D | CXXC1 |
| ADRA2B | MRGPRX3 |
| ADRB2 | CHD7 |
| AKR1D1 | SFRP1 |
| ALB | HSPB3 |
| ALDH1A1 | GPER1 |
| ANXA1 | ANTXR2 |
| AR | LAMA3 |
| ASRGL1 | HDAC4 |
| ASS1 | DDR2 |
| ATM | ADM |
| BCHE | WNT16 |
| CALCA | GPR35 |
| CX3CR1 | KAT8 |
| CYP17A1 | AHR |
| CYP19A1 | H4C2 |
| CYP27B1 | ACTG1 |
| CYP3A4 | NKRF |
| DRD2 | IL34 |
| ESR1 | CDK9 |
| ESR2 | DPEP1 |
| F12 | TUBA1B |
| FOXL2 | LPAR3 |
| GC | ACE2 |
| HAP1 | ITGA1 |
| HMGCR | WNT5A |
| INS | MIRLET7G |
| ITGB2 | RSPO3 |
| KCNJ11 | BMP2K |
| KCNJ8 | SEMA6A |
| NR1I2 | PDIA2 |
| NR3C1 | GBP1 |
| P3H1 | PTH1R |
| PCCB | FGF2 |
| PDE11A | PEX12 |
| PDE4A | IFNGR1 |
| PDE5A | SALL4 |
| PDE8B | HMGA2 |
| PGR | SLC12A1 |
| PHKG2 | WDR19 |
| PIK3CA | IDUA |
| PIK3CB | AKR1C4 |
| PIK3CD | MEG3 |
| PPARG | OXER1 |
| PPIB | GSTK1 |
| PRKCD | MUC1 |
| PRKDC | LINC01672 |
| PRLR | HSD11B1 |
| PRODH | IL10 |
| PTGER4 | IL12A |
| PTGS2 | RAP1A |
| PYCR1 | STAT3 |
| RARG | SOX4 |
| RXRA | H4C12 |
| RYR1 | SIRT6 |
| TNF | SALL1 |
| TRPV1 | GORASP1 |
| TYR | RPL31 |
| VCAM1 | FGFR1 |
| VDR | NSD2 |
| VKORC1 | TMEM64 |
| WLS | METTL21C |
| WNT4 | CHST3 |
| ADRB3 | HDAC5 |
| ALDH2 | IGSF23 |
| ASL | HNF1A |
| CAT | IFIT1 |
| CBS | MGP |
| CBSL | HSD17B2 |
| CPT1A | THSD7A |
| CTH | S100A4 |
| DBH | PLEKHO1 |
| GPT | ZNF143 |
| LARS2 | OSCAR |
| MAPK1 | IL1A |
| MPO | AKR1A1 |
| NR0B1 | DAAM2 |
| NTRK1 | RARRES2 |
| OTC | ASIC2 |
| PAH | TMCO1 |
| PCCA | ARMC5 |
| PRKAB1 | DANCR |
| PRKACA | MIR874 |
| SCT | HFE |
| SLC25A20 | RUNX3 |
| SLC6A4 | SMAD2 |
| SLC7A1 | CAV1 |
| SLC7A8 | HAMP |
| TLR4 | H4C15 |
| TPO | MIR98 |
| SOAT1 | PLOD1 |
| SLC25A4 | IRF2 |
| PRKAA1 | PAEP |
| HSD17B1 | MECOM |
| ANPEP | MIR889 |
| AKT1 | FGF17 |
| ABCC9 | FNDC1 |
| ABCC8 | SQSTM1 |
| ABCC2 | SH3PXD2B |
| ABCB11 | CILK1 |
| GGCX | CTNNB1 |
| F10 | FABP4 |
| BGLAP | CRK |
| HSP90AA1 | LTF |
| RELA | TRAF3 |
| MMP2 | ID4 |
| MMP9 | H19 |
| NFKBIA | B3GALT6 |
| CXCL8 | FZD4 |
| PRKCB | CLCN5 |
| IGF2 | LAMC2 |
| GSTM1 | MGLL |
| TP53 | MTNR1B |
| FASN | WDR1 |
| SOD1 | GATA4 |
| G6PD | CLCF1 |
| CSF2 | TGFBR1 |
| GSR | SPRY4 |
|  | BRD2 |
|  | ATP6V1H |
|  | TCF7L2 |
|  | IL6ST |
|  | RPL29 |
|  | CPE |
|  | NOS3 |
|  | MIR451A |
|  | LIFR |
|  | XXYLT1 |
|  | WNT3A |
|  | DDOST |
|  | SLC7A7 |
|  | PRDX3 |
|  | SAA3P |
|  | HSPD1 |
|  | CYP1B1 |
|  | PLK3 |
|  | CXXC5 |
|  | BRS3 |
|  | WDR11 |
|  | TFRC |
|  | CBFB |
|  | SULT2A1 |
|  | MIR885 |
|  | SLC25A19 |
|  | GTF2IRD1 |
|  | CCL11 |
|  | CCK |
|  | MACF1 |
|  | LCT |
|  | CCT2 |
|  | OR2AG1 |
|  | FLII |
|  | PARN |
|  | SIRT2 |
|  | ADAMTS2 |
|  | SMS |
|  | MST1 |
|  | HIVEP3 |
|  | ZFPM2 |
|  | VCL |
|  | CYP2B6 |
|  | CTSZ |
|  | ANOS1 |
|  | MIR100 |
|  | MIR449B |
|  | AHSG |
|  | NR5A1 |
|  | GPLD1 |
|  | EPHB2 |
|  | MIR96 |
|  | IFT122 |
|  | NSD1 |
|  | MOK |
|  | MIR30A |
|  | SLC9A3R1 |
|  | TWIST1 |
|  | RSU1 |
|  | DELEC1 |
|  | GFI1 |
|  | PSAT1 |
|  | MIR26B |
|  | HIRA |
|  | PARK7 |
|  | MIR146A |
|  | CANT1 |
|  | BAX |
|  | CDR1 |
|  | NTS |
|  | SRC |
|  | SET |
|  | GALT |
|  | SRM |
|  | CER1 |
|  | MYC |
|  | GSTM3 |
|  | KDM4B |
|  | TBL2 |
|  | GTF2I |
|  | PRDM2 |
|  | PLG |
|  | SERPINE1 |
|  | MIR545 |
|  | ALPL |
|  | KISS1R |
|  | TMEM135 |
|  | HPGDS |
|  | TRIP11 |
|  | MARCHF11 |
|  | RPS5 |
|  | IL20 |
|  | BCAR1 |
|  | SYT1 |
|  | PUM1 |
|  | PLOD2 |
|  | FGB |
|  | PHGDH |
|  | TLN1 |
|  | ASAH1 |
|  | SOX9 |
|  | ERCC6 |
|  | H4C6 |
|  | PFN1 |
|  | DUSP6 |
|  | MIR339 |
|  | CTHRC1 |
|  | SEC14L2 |
|  | USB1 |
|  | MIR200A |
|  | CDC73 |
|  | SRY |
|  | ITGAV |
|  | GTF2B |
|  | HNRNPL |
|  | LGMN |
|  | TGM2 |
|  | HLA-DQA1 |
|  | BMP3 |
|  | EFEMP2 |
|  | MTOR |
|  | GOLGA6A |
|  | AKAP11 |
|  | SAMD4A |
|  | CCL2 |
|  | MT1G |
|  | MSTN |
|  | EGFR |
|  | STRA6 |
|  | HSPB2 |
|  | IL37 |
|  | RAB3GAP1 |
|  | CRP |
|  | LMNA |
|  | CAP1 |
|  | GGPS1 |
|  | WRAP53 |
|  | C1QTNF4 |
|  | RNU4ATAC |
|  | BMPR1B |
|  | C1QTNF3 |
|  | PRKAR1A |
|  | DSPP |
|  | ENO1 |
|  | MIR139 |
|  | VANGL1 |
|  | ARHGEF3 |
|  | TAZ |
|  | ZDHHC13 |
|  | EPHA2 |
|  | WNT5B |
|  | KISS1 |
|  | DNASE1L3 |
|  | MUC16 |
|  | PTEN |
|  | CRY2 |
|  | DECR1 |
|  | PROP1 |
|  | FASLG |
|  | COMP |
|  | PRKAA2 |
|  | DNER |
|  | DKC1 |
|  | DCTN4 |
|  | MIR149 |
|  | CXCR6 |
|  | MIR375 |
|  | FGF13 |
|  | OXA1L |
|  | ZBTB20 |
|  | PSMA2 |
|  | MEN1 |
|  | MLXIPL |
|  | CASP1 |
|  | IL33 |
|  | SFRP4 |
|  | IL12RB1 |
|  | BACH1 |
|  | TMX2-CTNND1 |
|  | PPBP |
|  | IFT43 |
|  | EIF3K |
|  | MIR488 |
|  | FGF21 |
|  | CLOCK |
|  | FOSB |
|  | MIR137 |
|  | CAVIN1 |
|  | MMP14 |
|  | DNM3 |
|  | CREB1 |
|  | MIR455 |
|  | KRT18P57 |
|  | PRDM5 |
|  | MARK3 |
|  | CTC1 |
|  | IRAK3 |
|  | CST5 |
|  | SLC22A11 |
|  | GPBAR1 |
|  | TONSL |
|  | UCA1 |
|  | AMFR |
|  | IL17D |
|  | HDAC1 |
|  | H4C3 |
|  | OSBPL1A |
|  | KDM4A |
|  | ATP7B |
|  | OGN |
|  | MIR122 |
|  | PCNA |
|  | CXCL12 |
|  | PNP |
|  | AKR1B1 |
|  | PTPRF |
|  | TPI1 |
|  | TNPO3 |
|  | BRCA1 |
|  | DGCR2 |
|  | NRIP1 |
|  | RECQL4 |
|  | HOTAIR |
|  | MAPK7 |
|  | WWTR1 |
|  | NPY |
|  | PSMA5 |
|  | SLIT3 |
|  | MIR106B |
|  | SMURF2 |
|  | AMD1P2 |
|  | H4-16 |
|  | MMP17 |
|  | HSD3B2 |
|  | MIR498 |
|  | MIR223 |
|  | TCF7 |
|  | TERT |
|  | LINC00311 |
|  | VPS13B |
|  | TRIM16 |
|  | SPTBN1 |
|  | HPGD |
|  | GPD2 |
|  | GPR55 |
|  | SATB2 |
|  | JUND |
|  | PGLS |
|  | PKM |
|  | WDR35 |
|  | INSL3 |
|  | XIST |
|  | MIR182 |
|  | SNX10 |
|  | TSPAN5 |
|  | MGME1 |
|  | FLCN |
|  | MIR328 |
|  | LGR4 |
|  | AGBL2 |
|  | MSC |
|  | CRHR1 |
|  | EXT1 |
|  | KREMEN2 |
|  | CD38 |
|  | H4C11 |
|  | GALNS |
|  | DDIT3 |
|  | CLEC11A |
|  | ICAM1 |
|  | FYN |
|  | NOX1 |
|  | MCF2L |
|  | CASP3 |
|  | TMEM165 |
|  | COL11A1 |
|  | USP8 |
|  | AMH |
|  | CLU |
|  | CYP24A1 |
|  | BMP1 |
|  | IRF5 |
|  | P4HB |
|  | ADCY6 |
|  | POR |
|  | MITF |
|  | ZNF410 |
|  | CXCR4 |
|  | TET2 |
|  | PROKR2 |
|  | KHDRBS1 |
|  | ZNF469 |
|  | MIR152 |
|  | H4C9 |
|  | TUBB3 |
|  | TINF2 |
|  | AGRP |
|  | RAN |
|  | FRZB |
|  | ABCC6 |
|  | USE1 |
|  | SPRY1 |
|  | SOX6 |
|  | TESMIN |
|  | DOCK5 |
|  | POU2AF1 |
|  | FGF8 |
|  | MECP2 |
|  | LBP |
|  | MIR144 |
|  | RORA |
|  | MAFB |
|  | ATP6 |
|  | PIP5K1B |
|  | IL3 |
|  | SLCO2A1 |
|  | GRN |
|  | MIR539 |
|  | PADI1 |
|  | MMEL1 |
|  | CYB5A |
|  | ABO |
|  | PRDM6 |
|  | TAC3 |
|  | IREB2 |
|  | MIR2861 |
|  | ENPP1 |
|  | RNU1-1 |
|  | NUP62 |
|  | RAB31 |
|  | TTK |
|  | SMAD4 |
|  | PCSK7 |
|  | METTL3 |
|  | AIP |
|  | ACR |
|  | TNFSF13B |
|  | ATP12A |
|  | RNF19A |
|  | LTB4R |
|  | CX3CL1 |
|  | NNAT |
|  | MIR185 |
|  | IFT52 |
|  | HPRT1 |
|  | GPAA1 |
|  | STING1 |
|  | H4C5 |
|  | RABGEF1 |
|  | GNRHR |
|  | ALDH7A1 |
|  | TIMP2 |
|  | FTO |
|  | HBB |
|  | CSE1L |
|  | SMAD9 |
|  | THY1 |
|  | CSF3 |
|  | BDNF |
|  | G6PC |
|  | PTMA |
|  | NDUFAB1 |
|  | DICER1 |
|  | GK |
|  | KDM6B |
|  | CEACAM1 |
|  | RICTOR |
|  | VAMP7 |
|  | RPLP0 |
|  | PLXNA3 |
|  | CD44 |
|  | NHP2 |
|  | MIER1 |
|  | TXNRD1 |
|  | MIR126 |
|  | COL9A3 |
|  | RPGR |
|  | ELN |
|  | BICD2 |
|  | RAB7B |
|  | POLG2 |
|  | DUSP14 |
|  | NSMF |
|  | MALT1 |
|  | CASC11 |
|  | PTX3 |
|  | TSC22D3 |
|  | HDAC3 |
|  | MLPH |
|  | SMAD5 |
|  | COMT |
|  | RSPO1 |
|  | NFKB2 |
|  | SMUG1 |
|  | SLC51A |
|  | CDK1 |
|  | KDM5A |
|  | IFRD1 |
|  | SMAD7 |
|  | U2AF1 |
|  | APLN |
|  | AIMP2 |
|  | PLXNA2 |
|  | SLC26A2 |
|  | PIGT |
|  | ATG5 |
|  | MIR542 |
|  | TWNK |
|  | DOT1L |
|  | ASB16-AS1 |
|  | SPIB |
|  | TET1 |
|  | CRBN |
|  | DLL3 |
|  | PDIK1L |
|  | ZNF423 |
|  | SEPTIN4 |
|  | BMND2 |
|  | BMND8 |
|  | BMND3 |
|  | BMND4 |
|  | BMND5 |
|  | BMND11 |
|  | BMND13 |
|  | BMND14 |
|  | BMND10 |
|  | BMND9 |
|  | BMND6 |
|  | BMND7 |
|  | SP7 |
|  | CD36 |
|  | COL2A1 |
|  | IGFBP3 |
|  | NDP |
|  | PAX5 |
|  | TCIRG1 |
|  | BMP6 |
|  | TYROBP |
|  | LPCAT2 |
|  | LEPQTL1 |
|  | NPPB |
|  | IBSP |
|  | IL2 |
|  | IGFBP1 |
|  | IGFBP4 |
|  | UBXN11 |
|  | IL1R1 |
|  | KREMEN1 |
|  | IL11 |
|  | CD40LG |
|  | IL4 |
|  | GHRL |
|  | OXT |
|  | CNR1 |
|  | IGFBP2 |
|  | CALB1 |
|  | ERCC1 |
|  | AXIN2 |
|  | TGFB2 |
|  | EDN1 |
|  | IAPP |
|  | MEF2C |
|  | MIR133A1 |
|  | CFTR |
|  | FOSL1 |
|  | EGF |
|  | IL7 |
|  | RMND5B |
|  | APOB |
|  | GCM2 |
|  | CSK |
|  | SERPINC1 |
|  | IL18 |
|  | TNFSF10 |
|  | B2M |
|  | IL1RAPL2 |
|  | LIF |
|  | F3 |
|  | RHOA |
|  | DMP1 |
|  | MIR93 |
|  | SGO2 |
|  | MMP8 |
|  | CEBPA |
|  | WNT3 |
|  | MIR196A1 |
|  | MIR532 |
|  | MIR4713HG |
|  | PIRC66 |
|  | MIR18A |
|  | ATP6V0A2 |
|  | RAD54L |
|  | LOC109611589 |
|  | TGM1 |
|  | RTEL1-TNFRSF6B |
|  | TGFBR2 |
|  | GNAI2 |
|  | NBN |
|  | LOC110806263 |
|  | LDLR |
|  | SOX3 |
|  | G6PC1 |
|  | CDKN1C |
|  | HESX1 |
|  | STAG3 |
|  | LOC110806306 |
|  | GNPTAB |
|  | LOC101928371 |
|  | GBA |
|  | CYP2U1-AS1 |
|  | VLDLR |
|  | LRP8 |
|  | TP63 |
|  | FBN1 |
|  | CHEK2 |
|  | BRDT |
|  | PEX6 |
|  | INSL6 |
|  | TENT5A |
|  | BNC1 |
|  | SERPINH1 |
|  | MRPS22 |
|  | PSMC3IP |
|  | MIR550A1 |
|  | MAGEL2 |
|  | SUPT3H |
|  | WNT10B |
|  | NPM1 |
|  | DHX37 |
|  | MIR433 |
|  | SNRPN |
|  | NDN |
|  | GAA |
|  | OCA2 |
|  | MIR188 |
|  | PSC |
|  | ACD |
|  | EIF2B2 |
|  | PREPL |
|  | CHPT1 |
|  | MSH4 |
|  | CELIAC2 |
|  | CELIAC10 |
|  | CELIAC11 |
|  | CELIAC12 |
|  | CELIAC13 |
|  | CELIAC5 |
|  | CELIAC6 |
|  | CELIAC7 |
|  | CELIAC8 |
|  | CELIAC9 |
|  | COL3A1 |
|  | ASXL1 |
|  | SRSF2 |
|  | GLI2 |
|  | NUP107 |
|  | SLC34A3 |
|  | POU1F1 |
|  | AXIN1 |
|  | MTX2 |
|  | SERPINF1 |
|  | FMR1 |
|  | FLRT3 |
|  | IL17RD |
|  | PFAS |
|  | MESD |
|  | B4GALT7 |
|  | LOC107133510 |
|  | RUNX1 |
|  | RIN2 |
|  | OTX2 |
|  | FOXA2 |
|  | LHX4 |
|  | POLR3H |
|  | SPIDR |
|  | LOC106099062 |
|  | LOC110006319 |
|  | ANAPC1 |
|  | TONSL-AS1 |
|  | MTRR |
|  | ELANE |
|  | SLC40A1 |
|  | GNPTG |
|  | PHKA2 |
|  | BCKDHB |
|  | KDELR2 |
|  | ASXL2 |
|  | TRMT10A |
|  | CREB3L1 |
|  | COL4A5 |
|  | FGFR2 |
|  | MKRN3 |
|  | NF1 |
|  | NPAP1 |
|  | SIM1 |
|  | GJB2 |
|  | HEXA |
|  | SMN1 |
|  | SMN2 |
|  | SFMBT1 |
|  | IL2RA |
|  | FAT4 |
|  | THPO |
|  | STX1A |
|  | GATA1 |
|  | UROD |
|  | UROS |
|  | WFS1 |
|  | AAAS |
|  | SNORD116@ |
|  | FLNA |
|  | HERC2 |
|  | PWRN1 |
|  | IPW |
|  | SNORD115-1 |
|  | PWAR1 |
|  | SNORD116-1 |
|  | MKRN3-AS1 |
|  | TREM2 |
|  | PYGL |
|  | STN1 |
|  | CGA |
|  | TBCK |
|  | HLA-DRB1 |
|  | DMRT1 |
|  | MCM9 |
|  | CBL |
|  | LMX1B |
|  | MACROD2 |
|  | SEPSECS |
|  | MBTPS2 |
|  | PEX2 |
|  | MPL |
|  | APBB1 |
|  | CTBP1 |
|  | RPL10 |
|  | CPLX1 |
|  | NFIX |
|  | PIGG |
|  | SC5D |
|  | SCG2 |
|  | STAT5B |
|  | GALK1 |
|  | THRB |
|  | TMEM38B |
|  | CALR |
|  | NPC1 |
|  | CD79A |
|  | HLA-C |
|  | HSPA5 |
|  | TRPV6 |
|  | PRKACB |
|  | DHH |
|  | ZNF408 |
|  | DDX58 |
|  | HBB-LCR |
|  | SYK |
|  | ERCC8 |
|  | NR5A2 |
|  | SPP2 |
|  | POLE |
|  | TSPAN12 |
|  | IL17F |
|  | ARSB |
|  | PURA |
|  | IFT140 |
|  | PGBD3 |
|  | LARS2-AS1 |
|  | HFE-AS1 |
|  | MLX |
|  | TFR2 |
|  | FRAXA |
|  | NOTCH1 |
|  | UBE2Q2 |
|  | WDR37 |
|  | SYPL2 |
|  | TMEM60 |
|  | MINDY4 |
|  | FLNB |
|  | AMER1 |
|  | CYP21A2 |
|  | PEX1 |
|  | MMACHC |
|  | GMPPA |
|  | KITLG |
|  | F5 |
|  | NR1H4 |
|  | CP |
|  | FAS |
|  | POLR3B |
|  | PRKACG |
|  | PHEX |
|  | NOTCH3 |
|  | PRSS23 |
|  | ERCC2 |
|  | MIR483 |
|  | CSF3R |
|  | PEPD |
|  | COL5A2 |
|  | CLDN14 |
|  | OPA1 |
|  | LRRC4C |
|  | F2 |
|  | ZFPM2-AS1 |
|  | IFNA2 |
|  | FSHB |
|  | THRA |
|  | TJP2 |
|  | LAT2 |
|  | DEL17Q11.2 |
|  | TF |
|  | IFNA1 |
|  | COL9A1 |
|  | H2AC18 |
|  | SST |
|  | GNB1 |
|  | AGA |
|  | IRF6 |
|  | MYO5B |
|  | PEX14 |
|  | CBX2 |
|  | PEX3 |
|  | PEX5 |
|  | STX3 |
|  | PEX10 |
|  | PEX19 |
|  | INPP5E |
|  | NIPBL |
|  | PEX13 |
|  | PEX11B |
|  | PEX26 |
|  | PLCH2 |
|  | GATAD1 |
|  | SCUBE3 |
|  | SRP19 |
|  | PEX16 |
|  | PGAP3 |
|  | SETD5 |
|  | FCHO1 |
|  | JAGN1 |
|  | TRAPPC11 |
|  | SRPRA |
|  | NDP-AS1 |
|  | ASIC4-AS1 |
|  | LOC107032825 |
|  | EVR3 |
|  | GRD1 |
|  | GRD2 |
|  | HDC |
|  | DLX3 |
|  | JAG2 |
|  | AIRE |
|  | GGT1 |
|  | NR1H2 |
|  | IL13 |
|  | DNAH8 |
|  | CRH |
|  | LFNG |
|  | RBPJ |
|  | DLL1 |
|  | HEY1 |
|  | HEY2 |
|  | HEYL |
|  | HES7 |
|  | MESP2 |
|  | MAGEF1 |
|  | PRSS46P |
|  | IGF2R |
|  | OCRL |
|  | TNFRSF25 |
|  | TRPC3 |
|  | SELE |
|  | BRCA2 |
|  | TBC1D20 |
|  | RAD51 |
|  | NR1I3 |
|  | TG |
|  | SIL1 |
|  | BCAR4 |
|  | OPA1-AS1 |
|  | ICOSLG |
|  | PRSS3 |
|  | FANCA |
|  | VCP |
|  | PDGFRB |
|  | HELLS |
|  | PLCB3 |
|  | PNLIP |
|  | ERCC5 |
|  | UQCRFS1 |
|  | GALE |
|  | NEU1 |
|  | LTBP2 |
|  | XYLT1 |
|  | CLDN3 |
|  | CLDN4 |
|  | CLASP1 |
|  | EIF4H |
|  | VPS11 |
|  | RAB18 |
|  | TSEN2 |
|  | FKBP6 |
|  | TSEN15 |
|  | RAB3GAP2 |
|  | TSEN34 |
|  | TSEN54 |
|  | ABHD11 |
|  | BCL7B |
|  | KARS1 |
|  | AFF3 |
|  | TRAPPC2L |
|  | DNAJC30 |
|  | GTF2IRD2 |
|  | BUD23 |
|  | BBIP1 |
|  | VPS37D |
|  | TMPPE |
|  | METTL27 |
|  | TMEM270 |
|  | PHKA2-AS1 |
|  | GK-AS1 |
|  | MIF4GD-DT |
|  | LOC102723692 |
|  | PRS |
|  | LOC109504725 |
|  | LOC113939944 |
|  | LOC113687175 |
|  | DELXP21 |
|  | FWS |
|  | PBC5 |
|  | PBC2 |
|  | PBC3 |
|  | PBC4 |
|  | AGTR1 |
|  | COL10A1 |
|  | ALDH18A1 |
|  | ADAMTSL1 |
|  | HNF4A |
|  | MC4R |
|  | GLIS3 |
|  | CD4 |
|  | MC2R |
|  | TERF1 |
|  | NAGLU |
|  | MSX2 |
|  | ADAMTS4 |
|  | COLEC10 |
|  | LEF1 |
|  | CTLA4 |
|  | BLM |
|  | IL16 |
|  | PLAU |
|  | AFP |
|  | EXT2 |
|  | SERPINA7 |
|  | ITGA2 |
|  | MYB |
|  | CYP7A1 |
|  | CCR3 |
|  | IL31 |
|  | HSD3B1 |
|  | ITGA2B |
|  | DNAH9 |
|  | SLC19A1 |
|  | TCN2 |
|  | IER3IP1 |
|  | TCOF1 |
|  | IGFL3 |
|  | SHH |
|  | ADAMTS6 |
|  | UGT1A1 |
|  | ACP1 |
|  | TTF2 |
|  | LINC00339 |
|  | PDGFRA |
|  | LOC110386951 |
|  | IL21R |
|  | FBXO33 |
|  | CCR6 |
|  | MTR |
|  | LPL |
|  | LHCGR |
|  | ADAMTS19 |
|  | PTPN22 |
|  | ERN1 |
|  | FEN1 |
|  | EXO1 |
|  | M6PR |
|  | RAD52 |
|  | RECQL |
|  | TERF2 |
|  | RECQL5 |
|  | NUDT10 |
|  | CCND1 |
|  | SLC17A5 |
|  | FBN2 |
|  | AFF2 |
|  | VNN1 |
|  | TPD52 |
|  | AAGAB |
|  | FBXO5 |
|  | ZNF239 |
|  | PLEKHG1 |
|  | LOC110806262 |
|  | NRAS |
|  | GAST |
|  | MT-CO1 |
|  | CYP2R1 |
|  | FGFR3 |
|  | GAD2 |
|  | IGFBP6 |
|  | PSAP |
|  | STAR |
|  | INSR |
|  | ENTPD1 |
|  | PDCD1 |
|  | TBX19 |
|  | TNFRSF6B |
|  | ACAN |
|  | CD8A |
|  | CD34 |
|  | APC |
|  | BRSK1 |
|  | MCM8 |
|  | ANTXR1 |
|  | FGF18 |
|  | SLC10A2 |
|  | FBXW7 |
|  | CUL1 |
|  | RBX1 |
|  | SKP1 |
|  | LINC01094 |
|  | UVRAG-DT |
|  | LGALSL-DT |
|  | LINC02596 |
|  | LINC02597 |
|  | CYP1A2 |
|  | VIP |
|  | MIRLET7B |
|  | DNMT3A |
|  | CCL4 |
|  | ATP5PO |
|  | SLC30A8 |
|  | ITGA6 |
|  | ZP2 |
|  | PARP1 |
|  | SYNE3 |
|  | SLC19A2 |
|  | TNFRSF8 |
|  | EPO |
|  | IL9 |
|  | MIR497 |
|  | KDR |
|  | ADAMTS1 |
|  | NOBOX |
|  | SCG5 |
|  | MIR212 |
|  | EIF2AK1 |
|  | STAT5A |
|  | SLC12A6 |
|  | LRP2 |
|  | FUT2 |
|  | SSTR1 |
|  | CCR9 |
|  | ACKR2 |
|  | DCN |
|  | HLA-E |
|  | IL18R1 |
|  | CELA3B |
|  | PTPRD |
|  | GHRH |
|  | NR0B2 |
|  | LPA |
|  | FGF19 |
|  | CDKN2A |
|  | BMNCR |
|  | SCYL1 |
|  | CDKN1B |
|  | POLR1D |
|  | C11orf58 |
|  | CYP46A1 |
|  | RPL21 |
|  | KRT6B |
|  | PCAT2 |
|  | USP9X |
|  | DNAJC21 |
|  | RAD51C |
|  | PRKCQ |
|  | PSMC6 |
|  | MIF |
|  | TNFAIP3 |
|  | CLEC16A |
|  | TMEM150B |
|  | MLH1 |
|  | TOP1 |
|  | POLB |
|  | RPA1 |
|  | XRCC6 |
|  | XRCC5 |
|  | DIAPH2 |
|  | TAGLN |
|  | MRE11 |
|  | DHX9 |
|  | GDF9 |
|  | SYCE1 |
|  | WRNIP1 |
|  | PURG |
|  | SOHLH1 |
|  | SOHLH2 |
|  | STK11 |
|  | APH1A |
|  | PRPF3 |
|  | SETDB1 |
|  | SOX10 |
|  | SLC10A1 |
|  | TRH |
|  | TDGF1 |
|  | TCF7L1 |
|  | MIR181C |
|  | NOS2 |
|  | RAB6A |
|  | MPP7 |
|  | IHH |
|  | EIF2AK4 |
|  | MSX1 |
|  | TRPV5 |
|  | AVPR1B |
|  | EIF2S1 |
|  | CXCR3 |
|  | FIG4 |
|  | MKI67 |
|  | PRTN3 |
|  | KRT10 |
|  | NAGS |
|  | FDX1 |
|  | LTBP4 |
|  | NUP210 |
|  | HPX |
|  | COG7 |
|  | EIF1AD |
|  | LOC110806264 |
|  | LOC113839511 |
|  | ZYX |
|  | CD320 |
|  | NCAM1 |
|  | CRYAA |
|  | SSTR5 |
|  | SCARNA23 |
|  | ASCC1 |
|  | UPF1 |
|  | PTENP1 |
|  | ATF6 |
|  | GUSB |
|  | ATF4 |
|  | SLC7A6 |
|  | GORAB-AS1 |
|  | ABCB1 |
|  | ACHE |
|  | ADRA1A |
|  | ADRA1D |
|  | ADRA2B |
|  | ADRB2 |
|  | AKR1D1 |
|  | ALB |
|  | ALDH1A1 |
|  | ANXA1 |
|  | AR |
|  | ASRGL1 |
|  | ASS1 |
|  | ATM |
|  | BCHE |
|  | CALCA |
|  | CX3CR1 |
|  | CYP17A1 |
|  | CYP19A1 |
|  | CYP27B1 |
|  | CYP3A4 |
|  | DRD2 |
|  | ESR1 |
|  | ESR2 |
|  | F12 |
|  | FOXL2 |
|  | GC |
|  | HAP1 |
|  | HMGCR |
|  | INS |
|  | ITGB2 |
|  | KCNJ11 |
|  | KCNJ8 |
|  | NR1I2 |
|  | NR3C1 |
|  | P3H1 |
|  | PCCB |
|  | PDE11A |
|  | PDE4A |
|  | PDE5A |
|  | PDE8B |
|  | PGR |
|  | PHKG2 |
|  | PIK3CA |
|  | PIK3CB |
|  | PIK3CD |
|  | PPARG |
|  | PPIB |
|  | PRKCD |
|  | PRKDC |
|  | PRLR |
|  | PRODH |
|  | PTGER4 |
|  | PTGS2 |
|  | PYCR1 |
|  | RARG |
|  | RXRA |
|  | RYR1 |
|  | TNF |
|  | TRPV1 |
|  | TYR |
|  | VCAM1 |
|  | VDR |
|  | VKORC1 |
|  | WLS |
|  | WNT4 |
|  | ADRB3 |
|  | ALDH2 |
|  | ASL |
|  | CAT |
|  | CBS |
|  | CBSL |
|  | CPT1A |
|  | CTH |
|  | DBH |
|  | GPT |
|  | LARS2 |
|  | MAPK1 |
|  | MPO |
|  | NR0B1 |
|  | NTRK1 |
|  | OTC |
|  | PAH |
|  | PCCA |
|  | PRKAB1 |
|  | PRKACA |
|  | SCT |
|  | SLC25A20 |
|  | SLC6A4 |
|  | SLC7A1 |
|  | SLC7A8 |
|  | TLR4 |
|  | TPO |
|  | SOAT1 |
|  | SLC25A4 |
|  | PRKAA1 |
|  | HSD17B1 |
|  | ANPEP |
|  | AKT1 |
|  | ABCC9 |
|  | ABCC8 |
|  | ABCC2 |
|  | ABCB11 |
|  | GGCX |
|  | F10 |
|  | BGLAP |
|  | HSP90AA1 |
|  | RELA |
|  | MMP2 |
|  | MMP9 |
|  | NFKBIA |
|  | CXCL8 |
|  | PRKCB |
|  | IGF2 |
|  | GSTM1 |
|  | TP53 |
|  | FASN |
|  | SOD1 |
|  | G6PD |
|  | CSF2 |
|  | GSR |
