# Supplementary material for: Qing`e Pill Inhibits Osteoblast Ferroptosis via ATM Serine/Threonine Kinase (ATM) and the PI3K/AKT Pathway in Primary Osteoporosis
Source: Front Pharmacol. 2022 Jul 5;13:902102. doi: 10.3389/fphar.2022.902102 (PMC9294279; doi:10.3389/fphar.2022.902102)
Supplement: Supplementary file 4 [file Table5.docx]

**Supplement table 5 Top 30 genes with Maximal Clique Centrality**

| Rank | Name | Score |
| --- | --- | --- |
| 1 | AKT1 | 1.96E+11 |
| 2 | INS | 1.96E+11 |
| 3 | TP53 | 1.96E+11 |
| 4 | TNF | 1.96E+11 |
| 5 | ALB | 1.96E+11 |
| 6 | PTGS2 | 1.96E+11 |
| 7 | MMP9 | 1.95E+11 |
| 8 | NFKBIA | 1.95E+11 |
| 9 | CXCL8 | 1.95E+11 |
| 10 | PPARG | 1.94E+11 |
| 11 | TLR4 | 1.94E+11 |
| 12 | MMP2 | 1.81E+11 |
| 13 | MPO | 1.81E+11 |
| 14 | VCAM1 | 1.81E+11 |
| 15 | CAT | 1.00E+11 |
| 16 | CSF2 | 9.34E+10 |
| 17 | HSP90AA1 | 9.28E+09 |
| 18 | RELA | 8.61E+09 |
| 19 | GPT | 6.23E+09 |
| 20 | ESR1 | 1.97E+09 |
| 21 | MAPK1 | 1.34E+09 |
| 22 | AR | 1.27E+09 |
| 23 | NR3C1 | 1.17E+09 |
| 24 | PGR | 1.13E+09 |
| 25 | ATM | 6.24E+07 |
| 26 | IGF2 | 5.28E+07 |
| 27 | PIK3CA | 4.96E+07 |
| 28 | CYP19A1 | 8486010 |
| 29 | NTRK1 | 8067648 |
| 30 | BGLAP | 7746676 |
