# Supplementary material for: Qing`e Pill Inhibits Osteoblast Ferroptosis via ATM Serine/Threonine Kinase (ATM) and the PI3K/AKT Pathway in Primary Osteoporosis
Source: Front Pharmacol. 2022 Jul 5;13:902102. doi: 10.3389/fphar.2022.902102 (PMC9294279; doi:10.3389/fphar.2022.902102)
Supplement: Supplementary file 5 [file Table3.docx]

**Supplement table 3** Intersections of QEP and Osteoporosis

| Intersection |
| --- |
| ABCB1 |
| ACHE |
| ADRA1A |
| ADRA1D |
| ADRA2B |
| ADRB2 |
| AKR1D1 |
| ALB |
| ALDH1A1 |
| ANXA1 |
| AR |
| ASRGL1 |
| ASS1 |
| ATM |
| BCHE |
| CALCA |
| CX3CR1 |
| CYP17A1 |
| CYP19A1 |
| CYP27B1 |
| CYP3A4 |
| DRD2 |
| ESR1 |
| ESR2 |
| F12 |
| FOXL2 |
| GC |
| HAP1 |
| HMGCR |
| INS |
| ITGB2 |
| KCNJ11 |
| KCNJ8 |
| NR1I2 |
| NR3C1 |
| P3H1 |
| PCCB |
| PDE11A |
| PDE4A |
| PDE5A |
| PDE8B |
| PGR |
| PHKG2 |
| PIK3CA |
| PIK3CB |
| PIK3CD |
| PPARG |
| PPIB |
| PRKCD |
| PRKDC |
| PRLR |
| PRODH |
| PTGER4 |
| PTGS2 |
| PYCR1 |
| RARG |
| RXRA |
| RYR1 |
| TNF |
| TRPV1 |
| TYR |
| VCAM1 |
| VDR |
| VKORC1 |
| WLS |
| WNT4 |
| ADRB3 |
| ALDH2 |
| ASL |
| CAT |
| CBS |
| CBSL |
| CPT1A |
| CTH |
| DBH |
| GPT |
| LARS2 |
| MAPK1 |
| MPO |
| NR0B1 |
| NTRK1 |
| OTC |
| PAH |
| PCCA |
| PRKAB1 |
| PRKACA |
| SCT |
| SLC25A20 |
| SLC6A4 |
| SLC7A1 |
| SLC7A8 |
| TLR4 |
| TPO |
| SOAT1 |
| SLC25A4 |
| PRKAA1 |
| HSD17B1 |
| ANPEP |
| AKT1 |
| ABCC9 |
| ABCC8 |
| ABCC2 |
| ABCB11 |
| GGCX |
| F10 |
| BGLAP |
| HSP90AA1 |
| RELA |
| MMP2 |
| MMP9 |
| NFKBIA |
| CXCL8 |
| PRKCB |
| IGF2 |
| GSTM1 |
| TP53 |
| FASN |
| SOD1 |
| G6PD |
| CSF2 |
| GSR |
