# Supplementary material for: Qing`e Pill Inhibits Osteoblast Ferroptosis via ATM Serine/Threonine Kinase (ATM) and the PI3K/AKT Pathway in Primary Osteoporosis
Source: Front Pharmacol. 2022 Jul 5;13:902102. doi: 10.3389/fphar.2022.902102 (PMC9294279; doi:10.3389/fphar.2022.902102)
Supplement: Supplementary file 6 [file Table4.docx]

**Supplement table 4** GO/KEGG Enrichment Analysis

| ONTOLOGY | ID | p-value | Count |
| --- | --- | --- | --- |
| BP | GO:0031667 | 9.7887E-19 | 28 |
| BP | GO:0048732 | 4.0703E-18 | 26 |
| BP | GO:0008202 | 1.6879E-17 | 23 |
| BP | GO:0044706 | 4.892E-15 | 18 |
| BP | GO:0007565 | 6.8369E-15 | 17 |
| BP | GO:0006694 | 9.6499E-15 | 17 |
| BP | GO:0007584 | 6.0825E-14 | 17 |
| BP | GO:0034599 | 8.2763E-14 | 19 |
| BP | GO:0006979 | 1.3692E-13 | 22 |
| BP | GO:0042136 | 1.7655E-13 | 13 |
| BP | GO:0048608 | 5.3504E-13 | 21 |
| BP | GO:0061458 | 6.1145E-13 | 21 |
| BP | GO:0001101 | 8.0365E-13 | 19 |
| BP | GO:0042133 | 1.2023E-12 | 14 |
| BP | GO:1901617 | 1.9548E-12 | 17 |
| BP | GO:0007568 | 2.7625E-12 | 18 |
| BP | GO:0072593 | 4.1434E-12 | 17 |
| BP | GO:0097237 | 5.6344E-12 | 16 |
| BP | GO:0043434 | 6.0964E-12 | 20 |
| BP | GO:0048545 | 6.1159E-12 | 19 |
| BP | GO:0019932 | 6.9043E-12 | 20 |
| BP | GO:0060135 | 1.0109E-11 | 10 |
| BP | GO:1903409 | 2.2449E-11 | 12 |
| BP | GO:0035690 | 2.8052E-11 | 18 |
| BP | GO:2000377 | 3.2755E-11 | 14 |
| BP | GO:0032147 | 5.1381E-11 | 17 |
| BP | GO:1901653 | 5.6312E-11 | 18 |
| BP | GO:2000379 | 5.7876E-11 | 11 |
| BP | GO:0071496 | 6.7919E-11 | 17 |
| BP | GO:0001505 | 1.332E-10 | 17 |
| BP | GO:0046394 | 1.4036E-10 | 19 |
| BP | GO:0016053 | 1.4559E-10 | 19 |
| BP | GO:0051091 | 1.4676E-10 | 15 |
| BP | GO:0006066 | 2.0498E-10 | 17 |
| BP | GO:0045429 | 2.9548E-10 | 8 |
| BP | GO:0048871 | 3.1846E-10 | 19 |
| BP | GO:1904407 | 3.592E-10 | 8 |
| BP | GO:0051090 | 3.646E-10 | 18 |
| BP | GO:0046677 | 3.698E-10 | 16 |
| BP | GO:0030522 | 3.9109E-10 | 15 |
| BP | GO:0045428 | 4.0511E-10 | 9 |
| BP | GO:0032496 | 4.2261E-10 | 16 |
| BP | GO:0033273 | 4.6072E-10 | 10 |
| BP | GO:0046890 | 5.2085E-10 | 13 |
| BP | GO:0009266 | 6.062E-10 | 14 |
| BP | GO:0002237 | 7.4185E-10 | 16 |
| BP | GO:0071383 | 8.7822E-10 | 14 |
| BP | GO:0034614 | 9.5146E-10 | 12 |
| BP | GO:1903426 | 1.0491E-09 | 10 |
| BP | GO:0019216 | 1.2656E-09 | 17 |
| BP | GO:0001659 | 1.3324E-09 | 12 |
| BP | GO:0006809 | 1.6636E-09 | 9 |
| BP | GO:0031668 | 2.1634E-09 | 14 |
| BP | GO:0035296 | 2.2458E-09 | 11 |
| BP | GO:0050880 | 2.2458E-09 | 11 |
| BP | GO:0097746 | 2.2458E-09 | 11 |
| BP | GO:0035150 | 2.4184E-09 | 11 |
| BP | GO:0045834 | 2.7996E-09 | 11 |
| BP | GO:0046209 | 2.9413E-09 | 9 |
| BP | GO:1903428 | 3.1222E-09 | 8 |
| BP | GO:0008652 | 3.2812E-09 | 9 |
| BP | GO:0006367 | 3.4392E-09 | 12 |
| BP | GO:0014074 | 3.4726E-09 | 11 |
| BP | GO:0051092 | 3.4726E-09 | 11 |
| BP | GO:0000302 | 3.6107E-09 | 13 |
| BP | GO:0042445 | 3.6107E-09 | 13 |
| BP | GO:2001057 | 4.0659E-09 | 9 |
| BP | GO:0042446 | 4.5166E-09 | 9 |
| BP | GO:0062013 | 4.5939E-09 | 11 |
| BP | GO:1902652 | 5.2678E-09 | 11 |
| BP | GO:0001503 | 6.2678E-09 | 16 |
| BP | GO:0009410 | 6.4987E-09 | 14 |
| BP | GO:0062012 | 6.8819E-09 | 17 |
| BP | GO:1901607 | 1.0339E-08 | 8 |
| BP | GO:0098754 | 1.3384E-08 | 10 |
| BP | GO:0010038 | 1.3923E-08 | 15 |
| BP | GO:0006520 | 1.4977E-08 | 15 |
| BP | GO:0003018 | 1.6627E-08 | 11 |
| BP | GO:0046883 | 1.8581E-08 | 13 |
| BP | GO:0046165 | 1.8736E-08 | 11 |
| BP | GO:0071375 | 2.1531E-08 | 14 |
| BP | GO:0008209 | 2.1928E-08 | 6 |
| BP | GO:1901605 | 2.2233E-08 | 12 |
| BP | GO:0009914 | 2.2389E-08 | 14 |
| BP | GO:0050804 | 2.2582E-08 | 16 |
| BP | GO:0099177 | 2.3315E-08 | 16 |
| BP | GO:0032868 | 2.4199E-08 | 13 |
| BP | GO:0042737 | 2.5352E-08 | 10 |
| BP | GO:0035094 | 2.764E-08 | 7 |
| BP | GO:0044282 | 3.0005E-08 | 16 |
| BP | GO:0030879 | 3.1052E-08 | 10 |
| BP | GO:0043401 | 3.1476E-08 | 11 |
| BP | GO:0007566 | 3.6856E-08 | 7 |
| BP | GO:0019933 | 3.7182E-08 | 11 |
| BP | GO:0035264 | 3.7856E-08 | 10 |
| BP | GO:0097305 | 3.7969E-08 | 12 |
| BP | GO:0009755 | 4.172E-08 | 12 |
| BP | GO:0071880 | 4.2181E-08 | 6 |
| BP | GO:0071216 | 4.3717E-08 | 12 |
| BP | GO:0031669 | 4.58E-08 | 12 |
| BP | GO:1990748 | 4.6882E-08 | 9 |
| BP | GO:0010876 | 4.8455E-08 | 15 |
| BP | GO:0023061 | 5.0395E-08 | 16 |
| BP | GO:1901654 | 5.1427E-08 | 11 |
| BP | GO:1902930 | 5.34E-08 | 8 |
| BP | GO:0050994 | 5.5481E-08 | 7 |
| BP | GO:0001890 | 5.5518E-08 | 10 |
| BP | GO:2001233 | 5.8884E-08 | 15 |
| BP | GO:1990845 | 6.284E-08 | 10 |
| BP | GO:0051896 | 6.3046E-08 | 12 |
| BP | GO:0046889 | 7.1218E-08 | 8 |
| BP | GO:0097756 | 7.1218E-08 | 8 |
| BP | GO:0071875 | 7.5836E-08 | 6 |
| BP | GO:0006352 | 7.8709E-08 | 12 |
| BP | GO:0019318 | 7.8709E-08 | 12 |
| BP | GO:0070542 | 8.5752E-08 | 8 |
| BP | GO:0035265 | 9.0571E-08 | 11 |
| BP | GO:0001893 | 9.1044E-08 | 6 |
| BP | GO:0071222 | 9.5188E-08 | 11 |
| BP | GO:0050727 | 9.8103E-08 | 16 |
| BP | GO:0006869 | 1.0617E-07 | 14 |
| BP | GO:0006006 | 1.1582E-07 | 11 |
| BP | GO:0071229 | 1.1582E-07 | 11 |
| BP | GO:0046879 | 1.2042E-07 | 13 |
| BP | GO:0009612 | 1.2156E-07 | 11 |
| BP | GO:0045471 | 1.216E-07 | 9 |
| BP | GO:0016125 | 1.2747E-07 | 10 |
| BP | GO:0071219 | 1.338E-07 | 11 |
| BP | GO:0050806 | 1.4261E-07 | 10 |
| BP | GO:0019935 | 1.5422E-07 | 11 |
| BP | GO:1901655 | 1.585E-07 | 8 |
| BP | GO:0032869 | 1.6162E-07 | 11 |
| BP | GO:0043491 | 1.8212E-07 | 12 |
| BP | GO:0050810 | 1.8714E-07 | 8 |
| BP | GO:0009314 | 2.1065E-07 | 15 |
| BP | GO:0051897 | 2.2021E-07 | 10 |
| BP | GO:0006766 | 2.2097E-07 | 9 |
| BP | GO:0016054 | 2.3098E-07 | 12 |
| BP | GO:0046395 | 2.3098E-07 | 12 |
| BP | GO:0042359 | 2.5369E-07 | 5 |
| BP | GO:0016042 | 2.5472E-07 | 13 |
| BP | GO:0008217 | 3.0068E-07 | 10 |
| BP | GO:2001234 | 3.0371E-07 | 11 |
| BP | GO:0098869 | 3.2514E-07 | 8 |
| BP | GO:0006775 | 3.278E-07 | 6 |
| BP | GO:0061180 | 4.1914E-07 | 7 |
| BP | GO:0005996 | 4.3887E-07 | 12 |
| BP | GO:0048511 | 4.8928E-07 | 12 |
| BP | GO:0060444 | 5.0394E-07 | 5 |
| BP | GO:0031670 | 5.0659E-07 | 7 |
| BP | GO:1900076 | 5.0659E-07 | 7 |
| BP | GO:0014065 | 5.1447E-07 | 9 |
| BP | GO:1903522 | 5.2569E-07 | 12 |
| BP | GO:0015711 | 5.3412E-07 | 15 |
| BP | GO:0051222 | 5.493E-07 | 14 |
| BP | GO:0018105 | 5.6449E-07 | 12 |
| BP | GO:0008203 | 5.7623E-07 | 9 |
| BP | GO:0030850 | 6.4263E-07 | 6 |
| BP | GO:0042368 | 7.9819E-07 | 4 |
| BP | GO:0050900 | 8.2568E-07 | 15 |
| BP | GO:0002791 | 8.4663E-07 | 15 |
| BP | GO:0034612 | 8.8493E-07 | 12 |
| BP | GO:0007623 | 1.0249E-06 | 10 |
| BP | GO:0015718 | 1.0996E-06 | 9 |
| BP | GO:0022612 | 1.1308E-06 | 8 |
| BP | GO:0018209 | 1.2322E-06 | 12 |
| BP | GO:0014066 | 1.4499E-06 | 8 |
| BP | GO:0019218 | 1.4499E-06 | 8 |
| BP | GO:0007548 | 1.4791E-06 | 11 |
| BP | GO:0008406 | 1.5047E-06 | 10 |
| BP | GO:0014068 | 1.5345E-06 | 7 |
| BP | GO:1904951 | 1.5399E-06 | 14 |
| BP | GO:0036092 | 1.598E-06 | 4 |
| BP | GO:0015849 | 1.7491E-06 | 12 |
| BP | GO:0046942 | 1.7491E-06 | 12 |
| BP | GO:0071902 | 1.8044E-06 | 12 |
| BP | GO:0071398 | 1.8122E-06 | 6 |
| BP | GO:0042110 | 1.8876E-06 | 14 |
| BP | GO:0007596 | 1.9198E-06 | 12 |
| BP | GO:0045137 | 1.9243E-06 | 10 |
| BP | GO:0034754 | 1.9542E-06 | 8 |
| BP | GO:0060047 | 2.1057E-06 | 11 |
| BP | GO:0060603 | 2.1597E-06 | 5 |
| BP | GO:0042362 | 2.1682E-06 | 4 |
| BP | GO:0009408 | 2.1892E-06 | 9 |
| BP | GO:0007599 | 2.2373E-06 | 12 |
| BP | GO:0045833 | 2.24E-06 | 7 |
| BP | GO:0055074 | 2.2479E-06 | 14 |
| BP | GO:0050708 | 2.304E-06 | 14 |
| BP | GO:0050817 | 2.3061E-06 | 12 |
| BP | GO:0036473 | 2.4094E-06 | 7 |
| BP | GO:0046683 | 2.6005E-06 | 8 |
| BP | GO:0048638 | 2.679E-06 | 12 |
| BP | GO:0045123 | 2.7149E-06 | 6 |
| BP | GO:0048015 | 2.7579E-06 | 9 |
| BP | GO:0097193 | 2.8579E-06 | 11 |
| BP | GO:0071380 | 2.8764E-06 | 4 |
| BP | GO:0003015 | 2.9545E-06 | 11 |
| BP | GO:0008585 | 2.9833E-06 | 7 |
| BP | GO:0071356 | 3.0539E-06 | 11 |
| BP | GO:0048017 | 3.1568E-06 | 9 |
| BP | GO:0015908 | 3.1982E-06 | 7 |
| BP | GO:0007189 | 3.4202E-06 | 8 |
| BP | GO:0033002 | 3.5775E-06 | 10 |
| BP | GO:0050890 | 3.5965E-06 | 11 |
| BP | GO:0032768 | 3.6083E-06 | 6 |
| BP | GO:0031649 | 3.7427E-06 | 4 |
| BP | GO:0008286 | 3.8045E-06 | 8 |
| BP | GO:0033280 | 3.8854E-06 | 5 |
| BP | GO:0006936 | 3.9087E-06 | 12 |
| BP | GO:0051047 | 4.191E-06 | 13 |
| BP | GO:0046545 | 4.1915E-06 | 7 |
| BP | GO:0106106 | 4.2249E-06 | 8 |
| BP | GO:0120161 | 4.2249E-06 | 8 |
| BP | GO:0040014 | 4.3269E-06 | 6 |
| BP | GO:0046626 | 4.3269E-06 | 6 |
| BP | GO:0009084 | 4.7879E-06 | 4 |
| BP | GO:0070875 | 4.7879E-06 | 4 |
| BP | GO:0051384 | 4.9289E-06 | 8 |
| BP | GO:0042180 | 4.9656E-06 | 10 |
| BP | GO:0030072 | 5.3308E-06 | 10 |
| BP | GO:0015909 | 5.6192E-06 | 6 |
| BP | GO:0003085 | 6.0344E-06 | 4 |
| BP | GO:1901214 | 6.1244E-06 | 11 |
| BP | GO:0006631 | 7.3354E-06 | 12 |
| BP | GO:0006939 | 7.3912E-06 | 7 |
| BP | GO:0043627 | 7.813E-06 | 6 |
| BP | GO:0015850 | 8.0507E-06 | 10 |
| BP | GO:0008631 | 8.3179E-06 | 5 |
| BP | GO:0043279 | 8.3253E-06 | 7 |
| BP | GO:0009064 | 8.4577E-06 | 6 |
| BP | GO:1903829 | 8.4876E-06 | 11 |
| BP | GO:0090276 | 8.5549E-06 | 9 |
| BP | GO:0006874 | 8.6964E-06 | 13 |
| BP | GO:0150076 | 9.1448E-06 | 6 |
| BP | GO:0061756 | 9.3359E-06 | 5 |
| BP | GO:0070482 | 9.7538E-06 | 12 |
| BP | GO:0002683 | 9.7648E-06 | 13 |
| BP | GO:0042310 | 9.8764E-06 | 6 |
| BP | GO:0046660 | 9.9087E-06 | 7 |
| BP | GO:0051235 | 1.0085E-05 | 11 |
| BP | GO:0007249 | 1.0136E-05 | 10 |
| BP | GO:0003012 | 1.0224E-05 | 13 |
| BP | GO:0048538 | 1.0449E-05 | 5 |
| BP | GO:0031960 | 1.0597E-05 | 8 |
| BP | GO:1903532 | 1.1067E-05 | 12 |
| BP | GO:0071379 | 1.1218E-05 | 4 |
| BP | GO:0045776 | 1.1662E-05 | 5 |
| BP | GO:0060443 | 1.1662E-05 | 5 |
| BP | GO:0022407 | 1.2227E-05 | 12 |
| BP | GO:0007159 | 1.2274E-05 | 11 |
| BP | GO:0043405 | 1.2274E-05 | 11 |
| BP | GO:0006953 | 1.2984E-05 | 5 |
| BP | GO:0007530 | 1.3512E-05 | 4 |
| BP | GO:0034695 | 1.3512E-05 | 4 |
| BP | GO:0050995 | 1.3512E-05 | 4 |
| BP | GO:0048660 | 1.4422E-05 | 8 |
| BP | GO:0048659 | 1.5707E-05 | 8 |
| BP | GO:0032409 | 1.5737E-05 | 10 |
| BP | GO:0001649 | 1.6041E-05 | 9 |
| BP | GO:0009110 | 1.6134E-05 | 4 |
| BP | GO:0046697 | 1.6134E-05 | 4 |
| BP | GO:0051262 | 1.6385E-05 | 8 |
| BP | GO:0070997 | 1.6549E-05 | 11 |
| BP | GO:0030099 | 1.6763E-05 | 12 |
| BP | GO:0009063 | 1.7097E-05 | 7 |
| BP | GO:0009409 | 1.7658E-05 | 5 |
| BP | GO:0072503 | 1.8962E-05 | 13 |
| BP | GO:0009896 | 1.9769E-05 | 12 |
| BP | GO:0006914 | 2.0206E-05 | 13 |
| BP | GO:0061919 | 2.0206E-05 | 13 |
| BP | GO:0048771 | 2.1855E-05 | 8 |
| BP | GO:0001666 | 2.2063E-05 | 11 |
| BP | GO:0048568 | 2.2194E-05 | 12 |
| BP | GO:0060740 | 2.2473E-05 | 4 |
| BP | GO:0071466 | 2.2749E-05 | 8 |
| BP | GO:1900407 | 2.2983E-05 | 6 |
| BP | GO:0031331 | 2.322E-05 | 11 |
| BP | GO:0043122 | 2.4198E-05 | 9 |
| BP | GO:0030258 | 2.5014E-05 | 9 |
| BP | GO:0051048 | 2.5014E-05 | 9 |
| BP | GO:0043123 | 2.5617E-05 | 8 |
| BP | GO:0051353 | 2.5817E-05 | 5 |
| BP | GO:1904645 | 2.5817E-05 | 5 |
| BP | GO:0006706 | 2.6249E-05 | 4 |
| BP | GO:0045932 | 2.6249E-05 | 4 |
| BP | GO:0046885 | 2.6249E-05 | 4 |
| BP | GO:0032355 | 2.6824E-05 | 7 |
| BP | GO:0042593 | 2.7602E-05 | 9 |
| BP | GO:0006909 | 2.8391E-05 | 11 |
| BP | GO:0033500 | 2.8514E-05 | 9 |
| BP | GO:1903037 | 2.9058E-05 | 10 |
| BP | GO:0036293 | 2.9102E-05 | 11 |
| BP | GO:0060512 | 3.0471E-05 | 4 |
| BP | GO:2000108 | 3.0471E-05 | 4 |
| BP | GO:0006790 | 3.0571E-05 | 11 |
| BP | GO:0001660 | 3.0825E-05 | 3 |
| BP | GO:0003073 | 3.1486E-05 | 6 |
| BP | GO:0051289 | 3.3457E-05 | 6 |
| BP | GO:0044550 | 3.517E-05 | 4 |
| BP | GO:0042116 | 3.5525E-05 | 6 |
| BP | GO:0019229 | 3.6641E-05 | 5 |
| BP | GO:0031663 | 3.6641E-05 | 5 |
| BP | GO:0009411 | 3.7206E-05 | 7 |
| BP | GO:0050709 | 3.7206E-05 | 7 |
| BP | GO:0019217 | 3.7694E-05 | 6 |
| BP | GO:0009416 | 3.8228E-05 | 10 |
| BP | GO:0120162 | 3.9969E-05 | 6 |
| BP | GO:1902882 | 3.9969E-05 | 6 |
| BP | GO:0001763 | 4.18E-05 | 8 |
| BP | GO:0000050 | 4.2183E-05 | 3 |
| BP | GO:0060736 | 4.2183E-05 | 3 |
| BP | GO:0007204 | 4.3675E-05 | 10 |
| BP | GO:0007187 | 4.4225E-05 | 9 |
| BP | GO:0007611 | 4.4225E-05 | 9 |
| BP | GO:0033198 | 4.6128E-05 | 4 |
| BP | GO:0034694 | 4.6128E-05 | 4 |
| BP | GO:0042542 | 4.6481E-05 | 7 |
| BP | GO:0055123 | 4.6481E-05 | 7 |
| BP | GO:0043406 | 4.6977E-05 | 9 |
| BP | GO:0048661 | 5.0193E-05 | 6 |
| BP | GO:0002792 | 5.0685E-05 | 7 |
| BP | GO:0061041 | 5.0685E-05 | 7 |
| BP | GO:0046824 | 5.0688E-05 | 5 |
| BP | GO:0006575 | 5.1742E-05 | 8 |
| BP | GO:0043254 | 5.1805E-05 | 12 |
| BP | GO:0045907 | 5.2455E-05 | 4 |
| BP | GO:0055094 | 5.2455E-05 | 4 |
| BP | GO:0071295 | 5.2455E-05 | 4 |
| BP | GO:0030278 | 5.3576E-05 | 8 |
| BP | GO:0010506 | 5.3766E-05 | 10 |
| BP | GO:0046686 | 5.4775E-05 | 5 |
| BP | GO:0002573 | 5.5464E-05 | 8 |
| BP | GO:0002024 | 5.5978E-05 | 3 |
| BP | GO:0018958 | 5.6036E-05 | 6 |
| BP | GO:0016999 | 5.7571E-05 | 7 |
| BP | GO:0001655 | 5.8032E-05 | 10 |
| BP | GO:0000187 | 6.003E-05 | 7 |
| BP | GO:0002064 | 6.1465E-05 | 8 |
| BP | GO:0030073 | 6.1465E-05 | 8 |
| BP | GO:0030168 | 6.2574E-05 | 7 |
| BP | GO:0090316 | 6.2574E-05 | 7 |
| BP | GO:0006940 | 6.3707E-05 | 5 |
| BP | GO:1901606 | 6.5808E-05 | 6 |
| BP | GO:0071402 | 6.6975E-05 | 4 |
| BP | GO:0043523 | 6.7996E-05 | 8 |
| BP | GO:0097529 | 6.7996E-05 | 8 |
| BP | GO:0051341 | 6.9352E-05 | 6 |
| BP | GO:0062014 | 6.9352E-05 | 6 |
| BP | GO:1903531 | 7.0297E-05 | 8 |
| BP | GO:0046651 | 7.0599E-05 | 9 |
| BP | GO:0002679 | 7.2427E-05 | 3 |
| BP | GO:0019627 | 7.2427E-05 | 3 |
| BP | GO:0030656 | 7.2427E-05 | 3 |
| BP | GO:0070431 | 7.2427E-05 | 3 |
| BP | GO:0019748 | 7.3723E-05 | 5 |
| BP | GO:0032943 | 7.4675E-05 | 9 |
| BP | GO:0070873 | 7.524E-05 | 4 |
| BP | GO:0043467 | 7.6655E-05 | 7 |
| BP | GO:0043270 | 7.6786E-05 | 9 |
| BP | GO:0072330 | 8.0017E-05 | 10 |
| BP | GO:0072511 | 8.0385E-05 | 12 |
| BP | GO:0010742 | 8.4223E-05 | 4 |
| BP | GO:0046627 | 8.4223E-05 | 4 |
| BP | GO:0090077 | 8.4223E-05 | 4 |
| BP | GO:0090322 | 8.4223E-05 | 4 |
| BP | GO:0060348 | 8.5508E-05 | 8 |
| BP | GO:1903039 | 8.829E-05 | 8 |
| BP | GO:0009214 | 9.1745E-05 | 3 |
| BP | GO:0050667 | 9.1745E-05 | 3 |
| BP | GO:0071941 | 9.1745E-05 | 3 |
| BP | GO:0034405 | 9.3961E-05 | 4 |
| BP | GO:0045923 | 9.3961E-05 | 4 |
| BP | GO:0071276 | 9.3961E-05 | 4 |
| BP | GO:0002526 | 9.4081E-05 | 8 |
| BP | GO:0032386 | 9.6997E-05 | 11 |
| BP | GO:0007188 | 9.7092E-05 | 8 |
| BP | GO:0042752 | 9.8645E-05 | 6 |
| BP | GO:0030098 | 0.00010142 | 10 |
| BP | GO:0050805 | 0.00010409 | 5 |
| BP | GO:0016572 | 0.00010449 | 4 |
| BP | GO:0032350 | 0.00010449 | 4 |
| BP | GO:1900077 | 0.00010449 | 4 |
| BP | GO:0007569 | 0.0001086 | 6 |
| BP | GO:0006801 | 0.00011117 | 5 |
| BP | GO:0051480 | 0.00011124 | 10 |
| BP | GO:0042063 | 0.00011501 | 9 |
| BP | GO:1900182 | 0.00011861 | 5 |
| BP | GO:0006816 | 0.0001216 | 11 |
| BP | GO:0006937 | 0.00012571 | 7 |
| BP | GO:0016241 | 0.00012571 | 7 |
| BP | GO:0006695 | 0.00012643 | 5 |
| BP | GO:1903201 | 0.00012643 | 5 |
| BP | GO:0009743 | 0.00012797 | 8 |
| BP | GO:0060249 | 0.00012917 | 11 |
| BP | GO:0032102 | 0.00013333 | 10 |
| BP | GO:1902653 | 0.00013464 | 5 |
| BP | GO:0001780 | 0.00013981 | 3 |
| BP | GO:0032225 | 0.00013981 | 3 |
| BP | GO:0045986 | 0.00013981 | 3 |
| BP | GO:0060965 | 0.00013981 | 3 |
| BP | GO:0090153 | 0.00013981 | 3 |
| BP | GO:1902001 | 0.00013981 | 3 |
| BP | GO:1905038 | 0.00013981 | 3 |
| BP | GO:2000303 | 0.00013981 | 3 |
| BP | GO:0070661 | 0.0001412 | 9 |
| BP | GO:0090068 | 0.0001412 | 9 |
| BP | GO:0150077 | 0.00014122 | 4 |
| BP | GO:0050796 | 0.00015031 | 7 |
| BP | GO:0010907 | 0.00015531 | 4 |
| BP | GO:0046717 | 0.00015672 | 6 |
| BP | GO:0060326 | 0.00016396 | 9 |
| BP | GO:0051402 | 0.00016655 | 8 |
| BP | GO:1903034 | 0.00016686 | 7 |
| BP | GO:0000097 | 0.00016897 | 3 |
| BP | GO:0009070 | 0.00016897 | 3 |
| BP | GO:0031000 | 0.00016897 | 3 |
| BP | GO:0036270 | 0.00016897 | 3 |
| BP | GO:0045725 | 0.00016897 | 3 |
| BP | GO:1904996 | 0.00016897 | 3 |
| BP | GO:0032881 | 0.00017039 | 4 |
| BP | GO:0045088 | 0.00017333 | 11 |
| BP | GO:0035270 | 0.00017859 | 6 |
| BP | GO:0010565 | 0.00017868 | 7 |
| BP | GO:0009895 | 0.00018078 | 9 |
| BP | GO:0016126 | 0.00018197 | 5 |
| BP | GO:0061138 | 0.00018485 | 7 |
| BP | GO:0070266 | 0.00018649 | 4 |
| BP | GO:0042113 | 0.00018972 | 9 |
| BP | GO:0007252 | 0.00020181 | 3 |
| BP | GO:0071318 | 0.00020181 | 3 |
| BP | GO:0071827 | 0.00020367 | 4 |
| BP | GO:0045913 | 0.0002041 | 5 |
| BP | GO:0001819 | 0.00021735 | 11 |
| BP | GO:1905475 | 0.00021829 | 7 |
| BP | GO:0030518 | 0.00022033 | 6 |
| BP | GO:0072329 | 0.00022033 | 6 |
| BP | GO:0005978 | 0.00022195 | 4 |
| BP | GO:0009250 | 0.00022195 | 4 |
| BP | GO:0014075 | 0.00022195 | 4 |
| BP | GO:0048599 | 0.00022195 | 4 |
| BP | GO:0045787 | 0.00022308 | 10 |
| BP | GO:0008016 | 0.00023243 | 8 |
| BP | GO:0002544 | 0.00023852 | 3 |
| BP | GO:0006534 | 0.00023852 | 3 |
| BP | GO:0010888 | 0.00023852 | 3 |
| BP | GO:0032930 | 0.00023852 | 3 |
| BP | GO:0060149 | 0.00023852 | 3 |
| BP | GO:0060967 | 0.00023852 | 3 |
| BP | GO:1900409 | 0.00023852 | 3 |
| BP | GO:0071248 | 0.00024059 | 7 |
| BP | GO:0061614 | 0.00024139 | 4 |
| BP | GO:0001889 | 0.00024884 | 6 |
| BP | GO:0006112 | 0.00025445 | 5 |
| BP | GO:0022409 | 0.00025864 | 8 |
| BP | GO:0007292 | 0.00025897 | 6 |
| BP | GO:0001774 | 0.00026203 | 4 |
| BP | GO:0002269 | 0.00026203 | 4 |
| BP | GO:0051972 | 0.00026203 | 4 |
| BP | GO:0051224 | 0.0002647 | 7 |
| BP | GO:0034605 | 0.00026941 | 6 |
| BP | GO:0006525 | 0.00027928 | 3 |
| BP | GO:0061008 | 0.00028018 | 6 |
| BP | GO:0071825 | 0.0002839 | 4 |
| BP | GO:0097300 | 0.0002839 | 4 |
| BP | GO:0090257 | 0.00028722 | 8 |
| BP | GO:0002685 | 0.00029071 | 7 |
| BP | GO:0033135 | 0.00029129 | 6 |
| BP | GO:0045785 | 0.00029572 | 10 |
| BP | GO:1904950 | 0.00029983 | 7 |
| BP | GO:0070838 | 0.00030633 | 11 |
| BP | GO:0045540 | 0.00030705 | 4 |
| BP | GO:0106118 | 0.00030705 | 4 |
| BP | GO:0016049 | 0.00031175 | 11 |
| BP | GO:0017038 | 0.00031874 | 7 |
| BP | GO:0034341 | 0.00031874 | 7 |
| BP | GO:0010893 | 0.00032429 | 3 |
| BP | GO:1902884 | 0.00032429 | 3 |
| BP | GO:0038127 | 0.0003267 | 6 |
| BP | GO:0009994 | 0.00033152 | 4 |
| BP | GO:0050999 | 0.00033152 | 4 |
| BP | GO:0006606 | 0.00033923 | 6 |
| BP | GO:0071322 | 0.00035213 | 6 |
| BP | GO:0022898 | 0.00036105 | 8 |
| BP | GO:1901216 | 0.00036463 | 5 |
| BP | GO:0034764 | 0.00037025 | 7 |
| BP | GO:0006925 | 0.00037373 | 3 |
| BP | GO:0032928 | 0.00037373 | 3 |
| BP | GO:0051000 | 0.00037373 | 3 |
| BP | GO:0060065 | 0.00037373 | 3 |
| BP | GO:0097164 | 0.00038131 | 7 |
| BP | GO:1903076 | 0.00038291 | 5 |
| BP | GO:0010043 | 0.00038461 | 4 |
| BP | GO:0010212 | 0.00039314 | 6 |
| BP | GO:0071236 | 0.00039314 | 6 |
| BP | GO:0034284 | 0.00040423 | 7 |
| BP | GO:0006584 | 0.00041332 | 4 |
| BP | GO:0009712 | 0.00041332 | 4 |
| BP | GO:0071305 | 0.00042776 | 3 |
| BP | GO:0048754 | 0.0004378 | 6 |
| BP | GO:0001541 | 0.00044352 | 4 |
| BP | GO:0070301 | 0.00046306 | 5 |
| BP | GO:0051260 | 0.0004717 | 9 |
| BP | GO:0006749 | 0.00047526 | 4 |
| BP | GO:0010332 | 0.00047526 | 4 |
| BP | GO:0060688 | 0.00047526 | 4 |
| BP | GO:0007200 | 0.00048493 | 5 |
| BP | GO:0050729 | 0.00048632 | 6 |
| BP | GO:0050996 | 0.00048656 | 3 |
| BP | GO:0032388 | 0.00050715 | 7 |
| BP | GO:0042743 | 0.00050859 | 4 |
| BP | GO:0051055 | 0.00050859 | 4 |
| BP | GO:2001236 | 0.00052093 | 6 |
| BP | GO:0050673 | 0.00052897 | 10 |
| BP | GO:0071241 | 0.00053589 | 7 |
| BP | GO:0015980 | 0.0005426 | 8 |
| BP | GO:0017001 | 0.00054355 | 4 |
| BP | GO:0055081 | 0.00054355 | 4 |
| BP | GO:0061900 | 0.00054355 | 4 |
| BP | GO:0071385 | 0.00054355 | 4 |
| BP | GO:0009404 | 0.00055031 | 3 |
| BP | GO:0019430 | 0.00055031 | 3 |
| BP | GO:0050901 | 0.00055031 | 3 |
| BP | GO:0010001 | 0.00055074 | 7 |
| BP | GO:0019722 | 0.00055074 | 7 |
| BP | GO:1905952 | 0.00055742 | 6 |
| BP | GO:0060402 | 0.0005764 | 6 |
| BP | GO:0097755 | 0.00058018 | 4 |
| BP | GO:0046822 | 0.00058029 | 5 |
| BP | GO:0071887 | 0.00058029 | 5 |
| BP | GO:2001237 | 0.00058029 | 5 |
| BP | GO:0002793 | 0.00058122 | 8 |
| BP | GO:0018108 | 0.00060048 | 9 |
| BP | GO:0033138 | 0.00060619 | 5 |
| BP | GO:0032800 | 0.00061916 | 3 |
| BP | GO:0018212 | 0.00063681 | 9 |
| BP | GO:0071384 | 0.00065866 | 4 |
| BP | GO:1905953 | 0.00065866 | 4 |
| BP | GO:0033157 | 0.00066411 | 7 |
| BP | GO:0051170 | 0.00067895 | 6 |
| BP | GO:0032526 | 0.00068909 | 5 |
| BP | GO:2000278 | 0.00068909 | 5 |
| BP | GO:0002021 | 0.00069327 | 3 |
| BP | GO:0015721 | 0.00069327 | 3 |
| BP | GO:0071450 | 0.00069327 | 3 |
| BP | GO:0071451 | 0.00069327 | 3 |
| BP | GO:1902175 | 0.00069327 | 3 |
| BP | GO:0010676 | 0.00070058 | 4 |
| BP | GO:0070265 | 0.00070058 | 4 |
| BP | GO:0006633 | 0.00070105 | 6 |
| BP | GO:2001242 | 0.00072371 | 6 |
| BP | GO:0042982 | 0.00074437 | 4 |
| BP | GO:0090181 | 0.00074437 | 4 |
| BP | GO:0098739 | 0.00074888 | 5 |
| BP | GO:1904018 | 0.00075586 | 7 |
| BP | GO:0010894 | 0.0007728 | 3 |
| BP | GO:0032770 | 0.0007728 | 3 |
| BP | GO:1902932 | 0.0007728 | 3 |
| BP | GO:1904994 | 0.0007728 | 3 |
| BP | GO:1990776 | 0.0007728 | 3 |
| BP | GO:0045600 | 0.00079005 | 4 |
| BP | GO:0032411 | 0.00081247 | 5 |
| BP | GO:0050728 | 0.00082006 | 6 |
| BP | GO:0014823 | 0.00083768 | 4 |
| BP | GO:0046620 | 0.00084574 | 5 |
| BP | GO:0000303 | 0.00085792 | 3 |
| BP | GO:0005979 | 0.00085792 | 3 |
| BP | GO:0010962 | 0.00085792 | 3 |
| BP | GO:0033032 | 0.00085792 | 3 |
| BP | GO:0045822 | 0.00085792 | 3 |
| BP | GO:0060401 | 0.00087181 | 6 |
| BP | GO:0030282 | 0.00088001 | 5 |
| BP | GO:0046888 | 0.0008873 | 4 |
| BP | GO:0032963 | 0.0009153 | 5 |
| BP | GO:1904375 | 0.0009153 | 5 |
| BP | GO:0031348 | 0.00094612 | 7 |
| BP | GO:0000305 | 0.00094875 | 3 |
| BP | GO:0010743 | 0.00094875 | 3 |
| BP | GO:0045939 | 0.00094875 | 3 |
| BP | GO:0045940 | 0.00094875 | 3 |
| BP | GO:0046320 | 0.00094875 | 3 |
| BP | GO:0048265 | 0.00094875 | 3 |
| BP | GO:0071549 | 0.00094875 | 3 |
| BP | GO:1900180 | 0.00095164 | 5 |
| BP | GO:0030217 | 0.00096939 | 7 |
| BP | GO:0007613 | 0.00098904 | 5 |
| BP | GO:0010906 | 0.00098904 | 5 |
| BP | GO:0019915 | 0.00099269 | 4 |
| BP | GO:0035924 | 0.00099269 | 4 |
| BP | GO:0001963 | 0.00104546 | 3 |
| BP | GO:0002675 | 0.00104546 | 3 |
| BP | GO:0009154 | 0.00104546 | 3 |
| BP | GO:0010165 | 0.00104546 | 3 |
| BP | GO:0040018 | 0.00104546 | 3 |
| BP | GO:0045742 | 0.00104546 | 3 |
| BP | GO:1902895 | 0.00104546 | 3 |
| BP | GO:0071300 | 0.00104856 | 4 |
| BP | GO:0006898 | 0.00106043 | 8 |
| BP | GO:2001257 | 0.00107301 | 6 |
| BP | GO:0070371 | 0.00108208 | 8 |
| BP | GO:0033077 | 0.00110659 | 4 |
| BP | GO:0051966 | 0.00110659 | 4 |
| BP | GO:0002218 | 0.00112646 | 8 |
| BP | GO:0071346 | 0.00113656 | 6 |
| BP | GO:0001975 | 0.00114819 | 3 |
| BP | GO:0008210 | 0.00114819 | 3 |
| BP | GO:0009261 | 0.00114819 | 3 |
| BP | GO:0032228 | 0.00114819 | 3 |
| BP | GO:0042311 | 0.00114819 | 3 |
| BP | GO:0042744 | 0.00114819 | 3 |
| BP | GO:0045987 | 0.00114819 | 3 |
| BP | GO:0001704 | 0.00114965 | 5 |
| BP | GO:0033692 | 0.00116685 | 4 |
| BP | GO:0046434 | 0.00117228 | 7 |
| BP | GO:1905477 | 0.00119265 | 5 |
| BP | GO:0034765 | 0.00119572 | 10 |
| BP | GO:0060562 | 0.00119574 | 8 |
| BP | GO:0045926 | 0.00119982 | 7 |
| BP | GO:0009166 | 0.00120297 | 6 |
| BP | GO:0032103 | 0.00121957 | 8 |
| BP | GO:0051881 | 0.00122936 | 4 |
| BP | GO:0006732 | 0.00125304 | 9 |
| BP | GO:0045637 | 0.00125643 | 7 |
| BP | GO:0010737 | 0.00125707 | 3 |
| BP | GO:0016242 | 0.00125707 | 3 |
| BP | GO:0033028 | 0.00125707 | 3 |
| BP | GO:1901186 | 0.00125707 | 3 |
| BP | GO:1902692 | 0.00125707 | 3 |
| BP | GO:0042098 | 0.00127231 | 6 |
| BP | GO:0048639 | 0.00127231 | 6 |
| BP | GO:0051209 | 0.00128222 | 5 |
| BP | GO:1903524 | 0.00129418 | 4 |
| BP | GO:1903779 | 0.00129418 | 4 |
| BP | GO:0007369 | 0.0013081 | 6 |
| BP | GO:0005977 | 0.00136136 | 4 |
| BP | GO:0006081 | 0.00136136 | 4 |
| BP | GO:1901616 | 0.00136136 | 4 |
| BP | GO:0032148 | 0.00137224 | 3 |
| BP | GO:0048566 | 0.00137224 | 3 |
| BP | GO:0018107 | 0.00137667 | 5 |
| BP | GO:0051283 | 0.00137667 | 5 |
| BP | GO:0048167 | 0.001382 | 6 |
| BP | GO:0050730 | 0.00140712 | 7 |
| BP | GO:0007517 | 0.00141125 | 9 |
| BP | GO:1901292 | 0.00142013 | 6 |
| BP | GO:0032368 | 0.00142577 | 5 |
| BP | GO:0006073 | 0.00143093 | 4 |
| BP | GO:0021536 | 0.00143093 | 4 |
| BP | GO:0044042 | 0.00143093 | 4 |
| BP | GO:0034763 | 0.00147615 | 5 |
| BP | GO:0051282 | 0.00147615 | 5 |
| BP | GO:0034142 | 0.00149383 | 3 |
| BP | GO:0070423 | 0.00149383 | 3 |
| BP | GO:0030500 | 0.00150295 | 4 |
| BP | GO:0061045 | 0.00150295 | 4 |
| BP | GO:0032412 | 0.00153756 | 7 |
| BP | GO:0042594 | 0.00153934 | 6 |
| BP | GO:0043112 | 0.00158072 | 6 |
| BP | GO:0034504 | 0.00160622 | 7 |
| BP | GO:0030224 | 0.00162198 | 3 |
| BP | GO:0033762 | 0.00162198 | 3 |
| BP | GO:0035872 | 0.00162198 | 3 |
| BP | GO:0042554 | 0.00162198 | 3 |
| BP | GO:0097242 | 0.00162198 | 3 |
| BP | GO:1901099 | 0.00162198 | 3 |
| BP | GO:1903131 | 0.00162198 | 3 |
| BP | GO:2001240 | 0.00162198 | 3 |
| BP | GO:0030183 | 0.00163517 | 5 |
| BP | GO:0046887 | 0.00163517 | 5 |
| BP | GO:0051208 | 0.00163517 | 5 |
| BP | GO:0000271 | 0.00165449 | 4 |
| BP | GO:0010827 | 0.00165449 | 4 |
| BP | GO:0031016 | 0.00165449 | 4 |
| BP | GO:0045598 | 0.00169088 | 5 |
| BP | GO:0043062 | 0.00171971 | 9 |
| BP | GO:1901342 | 0.00171971 | 9 |
| BP | GO:0030193 | 0.00173411 | 4 |
| BP | GO:0071260 | 0.00173411 | 4 |
| BP | GO:0071333 | 0.00174796 | 5 |
| BP | GO:0014037 | 0.0017568 | 3 |
| BP | GO:0032885 | 0.0017568 | 3 |
| BP | GO:0042307 | 0.0017568 | 3 |
| BP | GO:0045730 | 0.0017568 | 3 |
| BP | GO:0018210 | 0.00180644 | 5 |
| BP | GO:0048565 | 0.00180644 | 5 |
| BP | GO:0006560 | 0.00181194 | 2 |
| BP | GO:0006570 | 0.00181194 | 2 |
| BP | GO:0010749 | 0.00181194 | 2 |
| BP | GO:0032070 | 0.00181194 | 2 |
| BP | GO:0032353 | 0.00181194 | 2 |
| BP | GO:0043471 | 0.00181194 | 2 |
| BP | GO:0048149 | 0.00181194 | 2 |
| BP | GO:0060456 | 0.00181194 | 2 |
| BP | GO:0070391 | 0.00181194 | 2 |
| BP | GO:0071223 | 0.00181194 | 2 |
| BP | GO:0090154 | 0.00181194 | 2 |
| BP | GO:0090336 | 0.00181194 | 2 |
| BP | GO:0140052 | 0.00181194 | 2 |
| BP | GO:1900222 | 0.00181194 | 2 |
| BP | GO:1903799 | 0.00181194 | 2 |
| BP | GO:2000304 | 0.00181194 | 2 |
| BP | GO:1900046 | 0.00181634 | 4 |
| BP | GO:0031334 | 0.00182664 | 7 |
| BP | GO:0050714 | 0.00182664 | 7 |
| BP | GO:0071331 | 0.00186635 | 5 |
| BP | GO:1903825 | 0.00186635 | 5 |
| BP | GO:1905039 | 0.00186635 | 5 |
| BP | GO:0032094 | 0.00189843 | 3 |
| BP | GO:0046676 | 0.00189843 | 3 |
| BP | GO:0060416 | 0.00189843 | 3 |
| BP | GO:1903523 | 0.00189843 | 3 |
| BP | GO:0045927 | 0.00190511 | 7 |
| BP | GO:0051052 | 0.00192326 | 9 |
| BP | GO:0071326 | 0.0019277 | 5 |
| BP | GO:0060048 | 0.00199052 | 5 |
| BP | GO:0009746 | 0.00204272 | 6 |
| BP | GO:0008207 | 0.00204698 | 3 |
| BP | GO:0009072 | 0.00204698 | 3 |
| BP | GO:0010661 | 0.00204698 | 3 |
| BP | GO:0033574 | 0.00204698 | 3 |
| BP | GO:0042417 | 0.00204698 | 3 |
| BP | GO:0045684 | 0.00204698 | 3 |
| BP | GO:0060969 | 0.00204698 | 3 |
| BP | GO:1904591 | 0.00204698 | 3 |
| BP | GO:0008584 | 0.00205482 | 5 |
| BP | GO:0009308 | 0.00205482 | 5 |
| BP | GO:0006970 | 0.00207922 | 4 |
| BP | GO:0048145 | 0.00207922 | 4 |
| BP | GO:0098659 | 0.00207922 | 4 |
| BP | GO:0099587 | 0.00207922 | 4 |
| BP | GO:2000106 | 0.00207922 | 4 |
| BP | GO:0042391 | 0.00208014 | 9 |
| BP | GO:0002433 | 0.00212062 | 5 |
| BP | GO:0007586 | 0.00212062 | 5 |
| BP | GO:0038096 | 0.00212062 | 5 |
| BP | GO:0046546 | 0.00212062 | 5 |
| BP | GO:0050715 | 0.00212062 | 5 |
| BP | GO:0045766 | 0.00214623 | 6 |
| BP | GO:0010507 | 0.00217239 | 4 |
| BP | GO:0048144 | 0.00217239 | 4 |
| BP | GO:0050818 | 0.00217239 | 4 |
| BP | GO:0097553 | 0.00218795 | 5 |
| BP | GO:0009187 | 0.00220257 | 3 |
| BP | GO:0071548 | 0.00220257 | 3 |
| BP | GO:0001976 | 0.00220522 | 2 |
| BP | GO:0006558 | 0.00220522 | 2 |
| BP | GO:0006559 | 0.00220522 | 2 |
| BP | GO:0006702 | 0.00220522 | 2 |
| BP | GO:0006853 | 0.00220522 | 2 |
| BP | GO:0031652 | 0.00220522 | 2 |
| BP | GO:0032351 | 0.00220522 | 2 |
| BP | GO:0033327 | 0.00220522 | 2 |
| BP | GO:0045899 | 0.00220522 | 2 |
| BP | GO:0046886 | 0.00220522 | 2 |
| BP | GO:0051974 | 0.00220522 | 2 |
| BP | GO:0060525 | 0.00220522 | 2 |
| BP | GO:0071803 | 0.00220522 | 2 |
| BP | GO:1902221 | 0.00220522 | 2 |
| BP | GO:1902222 | 0.00220522 | 2 |
| BP | GO:0006109 | 0.00225364 | 6 |
| BP | GO:0097530 | 0.00225682 | 5 |
| BP | GO:0034308 | 0.0022684 | 4 |
| BP | GO:1904705 | 0.0022684 | 4 |
| BP | GO:1990874 | 0.0022684 | 4 |
| BP | GO:0006733 | 0.00230885 | 6 |
| BP | GO:0010675 | 0.00232726 | 5 |
| BP | GO:0038094 | 0.00232726 | 5 |
| BP | GO:0050670 | 0.00236507 | 6 |
| BP | GO:0050873 | 0.0023653 | 3 |
| BP | GO:1902893 | 0.0023653 | 3 |
| BP | GO:0001776 | 0.00236729 | 4 |
| BP | GO:0034637 | 0.00236729 | 4 |
| BP | GO:0060291 | 0.00236729 | 4 |
| BP | GO:0032944 | 0.00242232 | 6 |
| BP | GO:0001892 | 0.00246912 | 4 |
| BP | GO:0048477 | 0.00246912 | 4 |
| BP | GO:0050707 | 0.0024806 | 6 |
| BP | GO:0021700 | 0.00253027 | 7 |
| BP | GO:0031647 | 0.00253027 | 7 |
| BP | GO:0021983 | 0.0025353 | 3 |
| BP | GO:0002431 | 0.0025482 | 5 |
| BP | GO:0007612 | 0.0025482 | 5 |
| BP | GO:0034103 | 0.00257391 | 4 |
| BP | GO:0043470 | 0.00257391 | 4 |
| BP | GO:2000177 | 0.00257391 | 4 |
| BP | GO:2000241 | 0.00262512 | 5 |
| BP | GO:0002674 | 0.00263506 | 2 |
| BP | GO:0006069 | 0.00263506 | 2 |
| BP | GO:0006563 | 0.00263506 | 2 |
| BP | GO:0010623 | 0.00263506 | 2 |
| BP | GO:0031392 | 0.00263506 | 2 |
| BP | GO:0032230 | 0.00263506 | 2 |
| BP | GO:0033197 | 0.00263506 | 2 |
| BP | GO:0045416 | 0.00263506 | 2 |
| BP | GO:0060442 | 0.00263506 | 2 |
| BP | GO:0060453 | 0.00263506 | 2 |
| BP | GO:0060742 | 0.00263506 | 2 |
| BP | GO:0061053 | 0.00268172 | 4 |
| BP | GO:0097306 | 0.00268172 | 4 |
| BP | GO:0002262 | 0.00270371 | 5 |
| BP | GO:0000096 | 0.00271267 | 3 |
| BP | GO:0032459 | 0.00271267 | 3 |
| BP | GO:1904646 | 0.00271267 | 3 |
| BP | GO:1990573 | 0.00271267 | 3 |
| BP | GO:0043281 | 0.00278801 | 6 |
| BP | GO:0070374 | 0.00278801 | 6 |
| BP | GO:1903035 | 0.00279259 | 4 |
| BP | GO:0009267 | 0.00286599 | 5 |
| BP | GO:0006195 | 0.00289751 | 3 |
| BP | GO:0046189 | 0.00289751 | 3 |
| BP | GO:0060333 | 0.00290657 | 4 |
| BP | GO:0017157 | 0.00291865 | 6 |
| BP | GO:0050777 | 0.00294972 | 5 |
| BP | GO:0001701 | 0.00298896 | 8 |
| BP | GO:0035987 | 0.00308993 | 3 |
| BP | GO:0051932 | 0.00308993 | 3 |
| BP | GO:0090278 | 0.00308993 | 3 |
| BP | GO:1900271 | 0.00308993 | 3 |
| BP | GO:0006703 | 0.003101 | 2 |
| BP | GO:0007171 | 0.003101 | 2 |
| BP | GO:0007494 | 0.003101 | 2 |
| BP | GO:0010459 | 0.003101 | 2 |
| BP | GO:0010745 | 0.003101 | 2 |
| BP | GO:0021984 | 0.003101 | 2 |
| BP | GO:0031650 | 0.003101 | 2 |
| BP | GO:0047484 | 0.003101 | 2 |
| BP | GO:0090399 | 0.003101 | 2 |
| BP | GO:0001678 | 0.00312247 | 5 |
| BP | GO:0016236 | 0.00312385 | 7 |
| BP | GO:0035249 | 0.00314398 | 4 |
| BP | GO:0070663 | 0.00326531 | 6 |
| BP | GO:0070167 | 0.00326751 | 4 |
| BP | GO:0002067 | 0.00329002 | 3 |
| BP | GO:0010883 | 0.00329002 | 3 |
| BP | GO:0048512 | 0.00329002 | 3 |
| BP | GO:0002758 | 0.00330286 | 7 |
| BP | GO:0045444 | 0.00333817 | 6 |
| BP | GO:0045089 | 0.00339965 | 8 |
| BP | GO:0030595 | 0.00341225 | 6 |
| BP | GO:0097191 | 0.00341225 | 6 |
| BP | GO:0034767 | 0.0034897 | 5 |
| BP | GO:0007622 | 0.00349788 | 3 |
| BP | GO:0048806 | 0.00349788 | 3 |
| BP | GO:2001239 | 0.00349788 | 3 |
| BP | GO:0048010 | 0.00352441 | 4 |
| BP | GO:0097327 | 0.00352441 | 4 |
| BP | GO:0034404 | 0.00356406 | 6 |
| BP | GO:0014854 | 0.00360254 | 2 |
| BP | GO:0048266 | 0.00360254 | 2 |
| BP | GO:2001279 | 0.00360254 | 2 |
| BP | GO:0042692 | 0.00362079 | 8 |
| BP | GO:0001894 | 0.00364183 | 6 |
| BP | GO:0002429 | 0.00368593 | 9 |
| BP | GO:0008542 | 0.00371361 | 3 |
| BP | GO:0009069 | 0.00371361 | 3 |
| BP | GO:0010107 | 0.00371361 | 3 |
| BP | GO:0035272 | 0.00371361 | 3 |
| BP | GO:0045933 | 0.00371361 | 3 |
| BP | GO:0060324 | 0.00371361 | 3 |
| BP | GO:0090199 | 0.00371361 | 3 |
| BP | GO:0051054 | 0.00372085 | 6 |
| BP | GO:0050769 | 0.00373718 | 9 |
| BP | GO:0002673 | 0.0037848 | 5 |
| BP | GO:1903706 | 0.00378898 | 9 |
| BP | GO:2001243 | 0.00379471 | 4 |
| BP | GO:0046661 | 0.00388702 | 5 |
| BP | GO:0014706 | 0.00391276 | 8 |
| BP | GO:0019395 | 0.00393499 | 4 |
| BP | GO:0002686 | 0.00393728 | 3 |
| BP | GO:0003044 | 0.00393728 | 3 |
| BP | GO:0071715 | 0.00393728 | 3 |
| BP | GO:1901571 | 0.00393728 | 3 |
| BP | GO:1904707 | 0.00393728 | 3 |
| BP | GO:1990090 | 0.00393728 | 3 |
| BP | GO:0022600 | 0.00407874 | 4 |
| BP | GO:0060079 | 0.00407874 | 4 |
| BP | GO:0000098 | 0.00413921 | 2 |
| BP | GO:0030238 | 0.00413921 | 2 |
| BP | GO:0030730 | 0.00413921 | 2 |
| BP | GO:0032352 | 0.00413921 | 2 |
| BP | GO:0033151 | 0.00413921 | 2 |
| BP | GO:0033604 | 0.00413921 | 2 |
| BP | GO:0034349 | 0.00413921 | 2 |
| BP | GO:0045898 | 0.00413921 | 2 |
| BP | GO:0046321 | 0.00413921 | 2 |
| BP | GO:0050961 | 0.00413921 | 2 |
| BP | GO:0050965 | 0.00413921 | 2 |
| BP | GO:0055089 | 0.00413921 | 2 |
| BP | GO:0071801 | 0.00413921 | 2 |
| BP | GO:1903729 | 0.00413921 | 2 |
| BP | GO:0001706 | 0.004169 | 3 |
| BP | GO:0002931 | 0.004169 | 3 |
| BP | GO:0046854 | 0.004169 | 3 |
| BP | GO:0031214 | 0.00420556 | 5 |
| BP | GO:0002027 | 0.00422601 | 4 |
| BP | GO:0034440 | 0.00422601 | 4 |
| BP | GO:0097006 | 0.00422601 | 4 |
| BP | GO:0051249 | 0.0043387 | 9 |
| BP | GO:0050863 | 0.00439366 | 7 |
| BP | GO:0043949 | 0.00440884 | 3 |
| BP | GO:0072523 | 0.00440884 | 3 |
| BP | GO:0097366 | 0.00440884 | 3 |
| BP | GO:0071695 | 0.00442802 | 5 |
| BP | GO:0007050 | 0.00449058 | 6 |
| BP | GO:0044264 | 0.00453123 | 4 |
| BP | GO:1990089 | 0.00465689 | 3 |
| BP | GO:0035051 | 0.00465874 | 5 |
| BP | GO:2000116 | 0.00467649 | 6 |
| BP | GO:0008630 | 0.00468927 | 4 |
| BP | GO:0019233 | 0.00468927 | 4 |
| BP | GO:0032414 | 0.00468927 | 4 |
| BP | GO:0042447 | 0.00471056 | 2 |
| BP | GO:0043217 | 0.00471056 | 2 |
| BP | GO:0045618 | 0.00471056 | 2 |
| BP | GO:0090335 | 0.00471056 | 2 |
| BP | GO:0050663 | 0.00477155 | 6 |
| BP | GO:0007009 | 0.00485098 | 4 |
| BP | GO:0055017 | 0.00485098 | 4 |
| BP | GO:0038093 | 0.00486802 | 6 |
| BP | GO:0060538 | 0.00489791 | 5 |
| BP | GO:0007632 | 0.00491323 | 3 |
| BP | GO:0045744 | 0.00491323 | 3 |
| BP | GO:0060537 | 0.00511732 | 8 |
| BP | GO:0042119 | 0.00514454 | 9 |
| BP | GO:0007635 | 0.0053161 | 2 |
| BP | GO:0008340 | 0.0053161 | 2 |
| BP | GO:0030540 | 0.0053161 | 2 |
| BP | GO:0032460 | 0.0053161 | 2 |
| BP | GO:0042953 | 0.0053161 | 2 |
| BP | GO:0044872 | 0.0053161 | 2 |
| BP | GO:0045187 | 0.0053161 | 2 |
| BP | GO:0048521 | 0.0053161 | 2 |
| BP | GO:1902931 | 0.0053161 | 2 |
| BP | GO:1903209 | 0.0053161 | 2 |
| BP | GO:0009062 | 0.00535849 | 4 |
| BP | GO:0015696 | 0.00535849 | 4 |
| BP | GO:0099565 | 0.00535849 | 4 |
| BP | GO:1904659 | 0.00535849 | 4 |
| BP | GO:0048872 | 0.00537206 | 6 |
| BP | GO:0016233 | 0.00545107 | 3 |
| BP | GO:0042306 | 0.00545107 | 3 |
| BP | GO:0006941 | 0.0055338 | 5 |
| BP | GO:0021761 | 0.00553525 | 4 |
| BP | GO:0032615 | 0.00573272 | 3 |
| BP | GO:1903078 | 0.00573272 | 3 |
| BP | GO:1903202 | 0.00573272 | 3 |
| BP | GO:0071214 | 0.00583104 | 7 |
| BP | GO:0104004 | 0.00583104 | 7 |
| BP | GO:0031623 | 0.00590038 | 4 |
| BP | GO:0006704 | 0.00595539 | 2 |
| BP | GO:0008090 | 0.00595539 | 2 |
| BP | GO:0010878 | 0.00595539 | 2 |
| BP | GO:0031998 | 0.00595539 | 2 |
| BP | GO:0035994 | 0.00595539 | 2 |
| BP | GO:0046459 | 0.00595539 | 2 |
| BP | GO:0060749 | 0.00595539 | 2 |
| BP | GO:0061377 | 0.00595539 | 2 |
| BP | GO:1900221 | 0.00595539 | 2 |
| BP | GO:0043525 | 0.00602295 | 3 |
| BP | GO:0072431 | 0.00602295 | 3 |
| BP | GO:1900408 | 0.00602295 | 3 |
| BP | GO:1902400 | 0.00602295 | 3 |
| BP | GO:0050821 | 0.00608291 | 5 |
| BP | GO:0002065 | 0.00608882 | 4 |
| BP | GO:0008645 | 0.00608882 | 4 |
| BP | GO:0060419 | 0.00608882 | 4 |
| BP | GO:0030336 | 0.00611744 | 7 |
| BP | GO:2000027 | 0.00614069 | 6 |
| BP | GO:0043200 | 0.00628123 | 4 |
| BP | GO:0030520 | 0.00632183 | 3 |
| BP | GO:1904589 | 0.00632183 | 3 |
| BP | GO:1901796 | 0.00637139 | 5 |
| BP | GO:1905330 | 0.00637139 | 5 |
| BP | GO:0005976 | 0.00647763 | 4 |
| BP | GO:0015749 | 0.00647763 | 4 |
| BP | GO:0042035 | 0.00647763 | 4 |
| BP | GO:0022408 | 0.00651917 | 5 |
| BP | GO:0001696 | 0.00662796 | 2 |
| BP | GO:0002076 | 0.00662796 | 2 |
| BP | GO:0010663 | 0.00662796 | 2 |
| BP | GO:0010666 | 0.00662796 | 2 |
| BP | GO:0016048 | 0.00662796 | 2 |
| BP | GO:0033189 | 0.00662796 | 2 |
| BP | GO:0045414 | 0.00662796 | 2 |
| BP | GO:0060438 | 0.00662796 | 2 |
| BP | GO:0060716 | 0.00662796 | 2 |
| BP | GO:0071800 | 0.00662796 | 2 |
| BP | GO:1902176 | 0.00662796 | 2 |
| BP | GO:1903798 | 0.00662796 | 2 |
| BP | GO:2000010 | 0.00662796 | 2 |
| BP | GO:0001836 | 0.00662941 | 3 |
| BP | GO:0045824 | 0.00662941 | 3 |
| BP | GO:0072413 | 0.00662941 | 3 |
| BP | GO:1902402 | 0.00662941 | 3 |
| BP | GO:1902403 | 0.00662941 | 3 |
| BP | GO:1902883 | 0.00662941 | 3 |
| BP | GO:0038061 | 0.00682193 | 5 |
| BP | GO:0006661 | 0.00688259 | 4 |
| BP | GO:0021782 | 0.00688259 | 4 |
| BP | GO:0034219 | 0.00688259 | 4 |
| BP | GO:1904062 | 0.00693265 | 7 |
| BP | GO:0046324 | 0.00694578 | 3 |
| BP | GO:2000045 | 0.00697695 | 5 |
| BP | GO:0006913 | 0.00703997 | 7 |
| BP | GO:1901222 | 0.00709122 | 4 |
| BP | GO:0007281 | 0.00724317 | 6 |
| BP | GO:0007405 | 0.00727097 | 3 |
| BP | GO:0032370 | 0.00727097 | 3 |
| BP | GO:0034113 | 0.00727097 | 3 |
| BP | GO:0046513 | 0.00727097 | 3 |
| BP | GO:0090303 | 0.00727097 | 3 |
| BP | GO:0030308 | 0.00729435 | 5 |
| BP | GO:0006677 | 0.00733337 | 2 |
| BP | GO:0010738 | 0.00733337 | 2 |
| BP | GO:0032495 | 0.00733337 | 2 |
| BP | GO:0042228 | 0.00733337 | 2 |
| BP | GO:0050802 | 0.00733337 | 2 |
| BP | GO:0060008 | 0.00733337 | 2 |
| BP | GO:0060261 | 0.00733337 | 2 |
| BP | GO:0051169 | 0.00736933 | 7 |
| BP | GO:0007173 | 0.00752091 | 4 |
| BP | GO:0002753 | 0.00760506 | 3 |
| BP | GO:0045453 | 0.00760506 | 3 |
| BP | GO:2000146 | 0.00771001 | 7 |
| BP | GO:0002698 | 0.00774205 | 4 |
| BP | GO:0003014 | 0.00774205 | 4 |
| BP | GO:0072089 | 0.00774205 | 4 |
| BP | GO:0019362 | 0.00778918 | 5 |
| BP | GO:0046496 | 0.00778918 | 5 |
| BP | GO:0003002 | 0.00794352 | 7 |
| BP | GO:0010631 | 0.00794352 | 7 |
| BP | GO:0007588 | 0.00794809 | 3 |
| BP | GO:0031571 | 0.00794809 | 3 |
| BP | GO:0044819 | 0.00794809 | 3 |
| BP | GO:1904377 | 0.00794809 | 3 |
| BP | GO:0002029 | 0.00807117 | 2 |
| BP | GO:0006067 | 0.00807117 | 2 |
| BP | GO:0009713 | 0.00807117 | 2 |
| BP | GO:0010829 | 0.00807117 | 2 |
| BP | GO:0019682 | 0.00807117 | 2 |
| BP | GO:0022401 | 0.00807117 | 2 |
| BP | GO:0030728 | 0.00807117 | 2 |
| BP | GO:0035357 | 0.00807117 | 2 |
| BP | GO:0042423 | 0.00807117 | 2 |
| BP | GO:0042749 | 0.00807117 | 2 |
| BP | GO:0046827 | 0.00807117 | 2 |
| BP | GO:0050951 | 0.00807117 | 2 |
| BP | GO:0055093 | 0.00807117 | 2 |
| BP | GO:0070920 | 0.00807117 | 2 |
| BP | GO:0071498 | 0.00807117 | 2 |
| BP | GO:1900273 | 0.00807117 | 2 |
| BP | GO:2000269 | 0.00807117 | 2 |
| BP | GO:2000810 | 0.00807117 | 2 |
| BP | GO:0044783 | 0.00830013 | 3 |
| BP | GO:0045670 | 0.00830013 | 3 |
| BP | GO:0046834 | 0.00830013 | 3 |
| BP | GO:2000378 | 0.00830013 | 3 |
| BP | GO:0090132 | 0.00830354 | 7 |
| BP | GO:0044272 | 0.00830693 | 5 |
| BP | GO:0050731 | 0.00830693 | 5 |
| BP | GO:0042089 | 0.00843105 | 4 |
| BP | GO:0071621 | 0.00843105 | 4 |
| BP | GO:0051187 | 0.00866121 | 3 |
| BP | GO:0008637 | 0.00866934 | 4 |
| BP | GO:0014013 | 0.00866934 | 4 |
| BP | GO:0042107 | 0.00866934 | 4 |
| BP | GO:0043542 | 0.00877734 | 6 |
| BP | GO:0002052 | 0.00884091 | 2 |
| BP | GO:0005980 | 0.00884091 | 2 |
| BP | GO:0021854 | 0.00884091 | 2 |
| BP | GO:0023058 | 0.00884091 | 2 |
| BP | GO:0032069 | 0.00884091 | 2 |
| BP | GO:0035584 | 0.00884091 | 2 |
| BP | GO:0045723 | 0.00884091 | 2 |
| BP | GO:0046058 | 0.00884091 | 2 |
| BP | GO:0046628 | 0.00884091 | 2 |
| BP | GO:0071404 | 0.00884091 | 2 |
| BP | GO:1901522 | 0.00884091 | 2 |
| BP | GO:0072524 | 0.00884811 | 5 |
| BP | GO:0006805 | 0.008912 | 4 |
| BP | GO:0031929 | 0.008912 | 4 |
| BP | GO:0051785 | 0.0090314 | 3 |
| BP | GO:0071897 | 0.0090338 | 5 |
| BP | GO:0090130 | 0.00905956 | 7 |
| BP | GO:0002221 | 0.00922217 | 5 |
| BP | GO:0009749 | 0.00922217 | 5 |
| BP | GO:0042108 | 0.00941073 | 3 |
| BP | GO:2000573 | 0.00941073 | 3 |
| BP | GO:0060541 | 0.00941323 | 5 |
| BP | GO:0016052 | 0.00960701 | 5 |
| BP | GO:0009251 | 0.00964216 | 2 |
| BP | GO:0022410 | 0.00964216 | 2 |
| BP | GO:0045821 | 0.00964216 | 2 |
| BP | GO:0046323 | 0.00979926 | 3 |
| BP | GO:0071230 | 0.00979926 | 3 |
| BP | GO:0044106 | 0.00992694 | 4 |
| BP | GO:1903038 | 0.00992694 | 4 |
| BP | GO:0050671 | 0.01019189 | 4 |
| BP | GO:2000243 | 0.01019702 | 3 |
| BP | GO:0050852 | 0.01020482 | 5 |
| BP | GO:0050870 | 0.01020482 | 5 |
| BP | GO:1902806 | 0.01020482 | 5 |
| BP | GO:0032946 | 0.0104614 | 4 |
| BP | GO:0010288 | 0.01047449 | 2 |
| BP | GO:0042738 | 0.01047449 | 2 |
| BP | GO:0044346 | 0.01047449 | 2 |
| BP | GO:0045606 | 0.01047449 | 2 |
| BP | GO:0050857 | 0.01047449 | 2 |
| BP | GO:1900078 | 0.01047449 | 2 |
| BP | GO:2000178 | 0.01047449 | 2 |
| BP | GO:0001756 | 0.01060405 | 3 |
| BP | GO:0007004 | 0.01060405 | 3 |
| BP | GO:0010517 | 0.01060405 | 3 |
| BP | GO:0050766 | 0.01060405 | 3 |
| BP | GO:0072091 | 0.01060405 | 3 |
| BP | GO:2001259 | 0.01060405 | 3 |
| BP | GO:0035637 | 0.01061726 | 5 |
| BP | GO:0051098 | 0.01086964 | 7 |
| BP | GO:0051403 | 0.01087605 | 6 |
| BP | GO:0050795 | 0.0110204 | 3 |
| BP | GO:0098656 | 0.01122793 | 6 |
| BP | GO:0070555 | 0.01125713 | 5 |
| BP | GO:0008211 | 0.01133746 | 2 |
| BP | GO:0030325 | 0.01133746 | 2 |
| BP | GO:0030813 | 0.01133746 | 2 |
| BP | GO:0044247 | 0.01133746 | 2 |
| BP | GO:0051197 | 0.01133746 | 2 |
| BP | GO:1904385 | 0.01133746 | 2 |
| BP | GO:0038034 | 0.0114461 | 3 |
| BP | GO:0097192 | 0.0114461 | 3 |
| BP | GO:1901215 | 0.01147615 | 5 |
| BP | GO:0060359 | 0.01158544 | 4 |
| BP | GO:0050678 | 0.0116315 | 7 |
| BP | GO:0033555 | 0.01188118 | 3 |
| BP | GO:0072401 | 0.01188118 | 3 |
| BP | GO:0072422 | 0.01188118 | 3 |
| BP | GO:0002696 | 0.01194684 | 7 |
| BP | GO:0042176 | 0.01210681 | 7 |
| BP | GO:0051146 | 0.01214283 | 6 |
| BP | GO:0072655 | 0.01217547 | 4 |
| BP | GO:0002360 | 0.01223066 | 2 |
| BP | GO:0022011 | 0.01223066 | 2 |
| BP | GO:0030194 | 0.01223066 | 2 |
| BP | GO:0032292 | 0.01223066 | 2 |
| BP | GO:0042745 | 0.01223066 | 2 |
| BP | GO:0071377 | 0.01223066 | 2 |
| BP | GO:1900048 | 0.01223066 | 2 |
| BP | GO:1903959 | 0.01223066 | 2 |
| BP | GO:1904353 | 0.01223066 | 2 |
| BP | GO:0014015 | 0.01232568 | 3 |
| BP | GO:0032677 | 0.01232568 | 3 |
| BP | GO:0050710 | 0.01232568 | 3 |
| BP | GO:0072395 | 0.01232568 | 3 |
| BP | GO:1903036 | 0.01232568 | 3 |
| BP | GO:0045765 | 0.01243141 | 7 |
| BP | GO:0051271 | 0.01259605 | 7 |
| BP | GO:0007422 | 0.01277963 | 3 |
| BP | GO:0010611 | 0.01277963 | 3 |
| BP | GO:1902117 | 0.01277963 | 3 |
| BP | GO:0031333 | 0.01278446 | 4 |
| BP | GO:0051588 | 0.01278446 | 4 |
| BP | GO:0070665 | 0.01278446 | 4 |
| BP | GO:0015893 | 0.01285156 | 5 |
| BP | GO:0016051 | 0.01285156 | 5 |
| BP | GO:0060078 | 0.01309612 | 4 |
| BP | GO:0000272 | 0.01315365 | 2 |
| BP | GO:0009651 | 0.01315365 | 2 |
| BP | GO:0035902 | 0.01315365 | 2 |
| BP | GO:0048643 | 0.01315365 | 2 |
| BP | GO:0050820 | 0.01315365 | 2 |
| BP | GO:0051953 | 0.01315365 | 2 |
| BP | GO:0060384 | 0.01315365 | 2 |
| BP | GO:0071514 | 0.01315365 | 2 |
| BP | GO:0072378 | 0.01315365 | 2 |
| BP | GO:0098810 | 0.01315365 | 2 |
| BP | GO:2000144 | 0.01315365 | 2 |
| BP | GO:0007492 | 0.01324305 | 3 |
| BP | GO:0070585 | 0.01341259 | 4 |
| BP | GO:0070372 | 0.01351036 | 6 |
| BP | GO:0043393 | 0.01357942 | 5 |
| BP | GO:0044242 | 0.01357942 | 5 |
| BP | GO:0046474 | 0.01357942 | 5 |
| BP | GO:0043312 | 0.01371 | 8 |
| BP | GO:0006278 | 0.01371598 | 3 |
| BP | GO:0032481 | 0.01371598 | 3 |
| BP | GO:0048678 | 0.01371598 | 3 |
| BP | GO:1901224 | 0.01371598 | 3 |
| BP | GO:0009952 | 0.01407984 | 5 |
| BP | GO:0001516 | 0.01410602 | 2 |
| BP | GO:0009065 | 0.01410602 | 2 |
| BP | GO:0009074 | 0.01410602 | 2 |
| BP | GO:0033598 | 0.01410602 | 2 |
| BP | GO:0034368 | 0.01410602 | 2 |
| BP | GO:0034369 | 0.01410602 | 2 |
| BP | GO:0034377 | 0.01410602 | 2 |
| BP | GO:0036296 | 0.01410602 | 2 |
| BP | GO:0046457 | 0.01410602 | 2 |
| BP | GO:0051123 | 0.01410602 | 2 |
| BP | GO:0060260 | 0.01410602 | 2 |
| BP | GO:1902003 | 0.01410602 | 2 |
| BP | GO:0002283 | 0.01418149 | 8 |
| BP | GO:0008306 | 0.01419844 | 3 |
| BP | GO:0014743 | 0.01419844 | 3 |
| BP | GO:0033143 | 0.01419844 | 3 |
| BP | GO:0090398 | 0.01419844 | 3 |
| BP | GO:2000300 | 0.01419844 | 3 |
| BP | GO:0010959 | 0.01433012 | 7 |
| BP | GO:0050867 | 0.01433012 | 7 |
| BP | GO:1904064 | 0.01439115 | 4 |
| BP | GO:0006110 | 0.01469045 | 3 |
| BP | GO:0043536 | 0.01469045 | 3 |
| BP | GO:1900034 | 0.01469045 | 3 |
| BP | GO:0040013 | 0.01469648 | 7 |
| BP | GO:0002224 | 0.01506802 | 4 |
| BP | GO:0061337 | 0.01506802 | 4 |
| BP | GO:0007263 | 0.01508736 | 2 |
| BP | GO:0014044 | 0.01508736 | 2 |
| BP | GO:0018200 | 0.01508736 | 2 |
| BP | GO:0034367 | 0.01508736 | 2 |
| BP | GO:0019359 | 0.01541387 | 4 |
| BP | GO:0019363 | 0.01541387 | 4 |
| BP | GO:0043524 | 0.01541387 | 4 |
| BP | GO:0061351 | 0.01541387 | 4 |
| BP | GO:0030811 | 0.01570323 | 3 |
| BP | GO:0048708 | 0.01570323 | 3 |
| BP | GO:0110110 | 0.01570323 | 3 |
| BP | GO:0008643 | 0.0157647 | 4 |
| BP | GO:0002446 | 0.01601214 | 8 |
| BP | GO:0000002 | 0.01609726 | 2 |
| BP | GO:0042759 | 0.01609726 | 2 |
| BP | GO:0045948 | 0.01609726 | 2 |
| BP | GO:0048147 | 0.01609726 | 2 |
| BP | GO:0048384 | 0.01609726 | 2 |
| BP | GO:0071480 | 0.01609726 | 2 |
| BP | GO:0090200 | 0.01609726 | 2 |
| BP | GO:0010833 | 0.01622403 | 3 |
| BP | GO:0032637 | 0.01622403 | 3 |
| BP | GO:0055117 | 0.01622403 | 3 |
| BP | GO:0071158 | 0.01622403 | 3 |
| BP | GO:1905954 | 0.01622403 | 3 |
| BP | GO:0016331 | 0.01648137 | 4 |
| BP | GO:0072525 | 0.01648137 | 4 |
| BP | GO:0031098 | 0.01679766 | 6 |
| BP | GO:0070588 | 0.01679766 | 6 |
| BP | GO:0055088 | 0.01684725 | 4 |
| BP | GO:1903169 | 0.01684725 | 4 |
| BP | GO:0050851 | 0.01703477 | 6 |
| BP | GO:0010543 | 0.01713532 | 2 |
| BP | GO:0030431 | 0.01713532 | 2 |
| BP | GO:0050482 | 0.01713532 | 2 |
| BP | GO:0090075 | 0.01713532 | 2 |
| BP | GO:1903963 | 0.01713532 | 2 |
| BP | GO:0016202 | 0.01721819 | 4 |
| BP | GO:0030902 | 0.01721819 | 4 |
| BP | GO:0043154 | 0.01729453 | 3 |
| BP | GO:1902803 | 0.01729453 | 3 |
| BP | GO:0034250 | 0.01759421 | 4 |
| BP | GO:0045807 | 0.01759421 | 4 |
| BP | GO:0002028 | 0.01784427 | 3 |
| BP | GO:0045682 | 0.01784427 | 3 |
| BP | GO:0038128 | 0.01820113 | 2 |
| BP | GO:0045922 | 0.01820113 | 2 |
| BP | GO:0051968 | 0.01820113 | 2 |
| BP | GO:0060674 | 0.01820113 | 2 |
| BP | GO:0065005 | 0.01820113 | 2 |
| BP | GO:0048588 | 0.0182329 | 5 |
| BP | GO:0071453 | 0.0182329 | 5 |
| BP | GO:0046486 | 0.01830083 | 7 |
| BP | GO:1901861 | 0.01836156 | 4 |
| BP | GO:0042058 | 0.01840368 | 3 |
| BP | GO:0048013 | 0.01840368 | 3 |
| BP | GO:0046777 | 0.01853557 | 5 |
| BP | GO:0001558 | 0.01873653 | 7 |
| BP | GO:0030856 | 0.01875292 | 4 |
| BP | GO:0042129 | 0.01875292 | 4 |
| BP | GO:0043535 | 0.01875292 | 4 |
| BP | GO:0048634 | 0.01875292 | 4 |
| BP | GO:0045844 | 0.01897277 | 3 |
| BP | GO:0048636 | 0.01897277 | 3 |
| BP | GO:0051781 | 0.01897277 | 3 |
| BP | GO:0043271 | 0.01914944 | 4 |
| BP | GO:0032872 | 0.01915083 | 5 |
| BP | GO:0010039 | 0.01929429 | 2 |
| BP | GO:0014072 | 0.01929429 | 2 |
| BP | GO:0030851 | 0.01929429 | 2 |
| BP | GO:0043278 | 0.01929429 | 2 |
| BP | GO:0048011 | 0.01929429 | 2 |
| BP | GO:0051194 | 0.01929429 | 2 |
| BP | GO:2001024 | 0.01929429 | 2 |
| BP | GO:0051188 | 0.01953411 | 6 |
| BP | GO:0097194 | 0.01955155 | 3 |
| BP | GO:1901863 | 0.01955155 | 3 |
| BP | GO:1901888 | 0.01955155 | 3 |
| BP | GO:0070302 | 0.0197794 | 5 |
| BP | GO:0006813 | 0.02009872 | 5 |
| BP | GO:0046427 | 0.02014004 | 3 |
| BP | GO:1901019 | 0.02014004 | 3 |
| BP | GO:0007519 | 0.02037007 | 4 |
| BP | GO:1903305 | 0.02037007 | 4 |
| BP | GO:0031128 | 0.02041442 | 2 |
| BP | GO:0032212 | 0.02041442 | 2 |
| BP | GO:0034205 | 0.02041442 | 2 |
| BP | GO:0042533 | 0.02041442 | 2 |
| BP | GO:0042534 | 0.02041442 | 2 |
| BP | GO:0043276 | 0.02041442 | 2 |
| BP | GO:0043516 | 0.02041442 | 2 |
| BP | GO:0048048 | 0.02041442 | 2 |
| BP | GO:0071868 | 0.02041442 | 2 |
| BP | GO:0071870 | 0.02041442 | 2 |
| BP | GO:0097009 | 0.02041442 | 2 |
| BP | GO:0015844 | 0.02073822 | 3 |
| BP | GO:0046849 | 0.02073822 | 3 |
| BP | GO:0071674 | 0.02073822 | 3 |
| BP | GO:0000723 | 0.0212099 | 4 |
| BP | GO:0002701 | 0.02156112 | 2 |
| BP | GO:0010259 | 0.02156112 | 2 |
| BP | GO:0015695 | 0.02156112 | 2 |
| BP | GO:0032735 | 0.02156112 | 2 |
| BP | GO:0051354 | 0.02156112 | 2 |
| BP | GO:0071312 | 0.02156112 | 2 |
| BP | GO:1902991 | 0.02156112 | 2 |
| BP | GO:1905332 | 0.02156112 | 2 |
| BP | GO:0045931 | 0.02163768 | 4 |
| BP | GO:0051251 | 0.02170588 | 6 |
| BP | GO:0006672 | 0.02196373 | 3 |
| BP | GO:1904894 | 0.02196373 | 3 |
| BP | GO:2000117 | 0.02196373 | 3 |
| BP | GO:0050680 | 0.02250906 | 4 |
| BP | GO:0007589 | 0.02259106 | 3 |
| BP | GO:1901184 | 0.02259106 | 3 |
| BP | GO:0006699 | 0.02273401 | 2 |
| BP | GO:0014808 | 0.02273401 | 2 |
| BP | GO:0051973 | 0.02273401 | 2 |
| BP | GO:2001171 | 0.02273401 | 2 |
| BP | GO:0045017 | 0.02383617 | 5 |
| BP | GO:0042100 | 0.02387485 | 3 |
| BP | GO:0042102 | 0.02387485 | 3 |
| BP | GO:0044070 | 0.02387485 | 3 |
| BP | GO:0045069 | 0.02387485 | 3 |
| BP | GO:0051196 | 0.02387485 | 3 |
| BP | GO:0043243 | 0.02393271 | 2 |
| BP | GO:0045777 | 0.02393271 | 2 |
| BP | GO:0090050 | 0.02393271 | 2 |
| BP | GO:1903514 | 0.02393271 | 2 |
| BP | GO:1904358 | 0.02393271 | 2 |
| BP | GO:2000142 | 0.02393271 | 2 |
| BP | GO:2000273 | 0.02393271 | 2 |
| BP | GO:0038095 | 0.02431545 | 4 |
| BP | GO:0051100 | 0.02431545 | 4 |
| BP | GO:0010660 | 0.02453132 | 3 |
| BP | GO:0090277 | 0.02453132 | 3 |
| BP | GO:2001169 | 0.02453132 | 3 |
| BP | GO:0043433 | 0.02478039 | 4 |
| BP | GO:0051924 | 0.02492828 | 5 |
| BP | GO:0003298 | 0.02515684 | 2 |
| BP | GO:0003301 | 0.02515684 | 2 |
| BP | GO:0007190 | 0.02515684 | 2 |
| BP | GO:0046326 | 0.02515684 | 2 |
| BP | GO:0046825 | 0.02515684 | 2 |
| BP | GO:0048009 | 0.02515684 | 2 |
| BP | GO:0051590 | 0.02515684 | 2 |
| BP | GO:0061049 | 0.02515684 | 2 |
| BP | GO:0071867 | 0.02515684 | 2 |
| BP | GO:0071869 | 0.02515684 | 2 |
| BP | GO:2000279 | 0.02515684 | 2 |
| BP | GO:0006942 | 0.02519749 | 3 |
| BP | GO:0030316 | 0.02519749 | 3 |
| BP | GO:0043255 | 0.02519749 | 3 |
| BP | GO:0044773 | 0.02519749 | 3 |
| BP | GO:0051591 | 0.02519749 | 3 |
| BP | GO:2001022 | 0.02519749 | 3 |
| BP | GO:0010634 | 0.02525068 | 4 |
| BP | GO:1901990 | 0.02562413 | 7 |
| BP | GO:0006839 | 0.02567398 | 5 |
| BP | GO:0002042 | 0.02587337 | 3 |
| BP | GO:0050764 | 0.02587337 | 3 |
| BP | GO:0050848 | 0.02587337 | 3 |
| BP | GO:0060191 | 0.02587337 | 3 |
| BP | GO:0007389 | 0.02617451 | 7 |
| BP | GO:0010469 | 0.02620739 | 4 |
| BP | GO:0030501 | 0.02640602 | 2 |
| BP | GO:0033146 | 0.02640602 | 2 |
| BP | GO:0038179 | 0.02640602 | 2 |
| BP | GO:1905521 | 0.02640602 | 2 |
| BP | GO:2000648 | 0.02640602 | 2 |
| BP | GO:1901570 | 0.02655894 | 3 |
| BP | GO:0046488 | 0.02669382 | 4 |
| BP | GO:0032200 | 0.02718566 | 4 |
| BP | GO:0008654 | 0.02720804 | 5 |
| BP | GO:0072659 | 0.02720804 | 5 |
| BP | GO:0010657 | 0.02725421 | 3 |
| BP | GO:0030301 | 0.02725421 | 3 |
| BP | GO:0035282 | 0.02725421 | 3 |
| BP | GO:0009108 | 0.0276005 | 5 |
| BP | GO:0042026 | 0.02767988 | 2 |
| BP | GO:0044058 | 0.02767988 | 2 |
| BP | GO:0045124 | 0.02767988 | 2 |
| BP | GO:0046621 | 0.02767988 | 2 |
| BP | GO:2000008 | 0.02767988 | 2 |
| BP | GO:0007631 | 0.02795915 | 3 |
| BP | GO:0048469 | 0.02818559 | 4 |
| BP | GO:0030148 | 0.02867377 | 3 |
| BP | GO:0031330 | 0.02879949 | 5 |
| BP | GO:0022412 | 0.02884614 | 6 |
| BP | GO:0006308 | 0.02897807 | 2 |
| BP | GO:0040019 | 0.02897807 | 2 |
| BP | GO:0042572 | 0.02897807 | 2 |
| BP | GO:0045616 | 0.02897807 | 2 |
| BP | GO:0061028 | 0.02897807 | 2 |
| BP | GO:0071347 | 0.02920725 | 4 |
| BP | GO:2001235 | 0.02920725 | 4 |
| BP | GO:0032091 | 0.02939804 | 3 |
| BP | GO:0032652 | 0.02939804 | 3 |
| BP | GO:0034766 | 0.02939804 | 3 |
| BP | GO:0044839 | 0.02961693 | 5 |
| BP | GO:0043534 | 0.02972625 | 4 |
| BP | GO:0072331 | 0.0300311 | 5 |
| BP | GO:0030593 | 0.03013197 | 3 |
| BP | GO:0046928 | 0.03013197 | 3 |
| BP | GO:0002285 | 0.0302507 | 4 |
| BP | GO:0010613 | 0.03030021 | 2 |
| BP | GO:0031641 | 0.03030021 | 2 |
| BP | GO:0042149 | 0.03030021 | 2 |
| BP | GO:0042181 | 0.03030021 | 2 |
| BP | GO:1901031 | 0.03030021 | 2 |
| BP | GO:0001667 | 0.03056253 | 7 |
| BP | GO:0010821 | 0.03078062 | 4 |
| BP | GO:0006836 | 0.03087041 | 5 |
| BP | GO:0015748 | 0.03087552 | 3 |
| BP | GO:0032006 | 0.03087552 | 3 |
| BP | GO:0045165 | 0.03129555 | 5 |
| BP | GO:0002286 | 0.0316287 | 3 |
| BP | GO:0003300 | 0.0316287 | 3 |
| BP | GO:0044774 | 0.0316287 | 3 |
| BP | GO:0006692 | 0.03164594 | 2 |
| BP | GO:0006693 | 0.03164594 | 2 |
| BP | GO:0010863 | 0.03164594 | 2 |
| BP | GO:0014742 | 0.03164594 | 2 |
| BP | GO:0032365 | 0.03164594 | 2 |
| BP | GO:0042088 | 0.03164594 | 2 |
| BP | GO:0045687 | 0.03164594 | 2 |
| BP | GO:0045746 | 0.03164594 | 2 |
| BP | GO:0050850 | 0.03164594 | 2 |
| BP | GO:0070296 | 0.03164594 | 2 |
| BP | GO:0043409 | 0.03185688 | 4 |
| BP | GO:1902105 | 0.03215687 | 5 |
| BP | GO:0043502 | 0.03239149 | 3 |
| BP | GO:0007409 | 0.03277091 | 7 |
| BP | GO:0010828 | 0.03301491 | 2 |
| BP | GO:0014002 | 0.03301491 | 2 |
| BP | GO:0030225 | 0.03301491 | 2 |
| BP | GO:0032965 | 0.03301491 | 2 |
| BP | GO:0042987 | 0.03301491 | 2 |
| BP | GO:0070897 | 0.03301491 | 2 |
| BP | GO:0090311 | 0.03301491 | 2 |
| BP | GO:0071156 | 0.03316386 | 3 |
| BP | GO:0007411 | 0.0339238 | 5 |
| BP | GO:0001676 | 0.0339458 | 3 |
| BP | GO:0014897 | 0.0339458 | 3 |
| BP | GO:0051783 | 0.03407525 | 4 |
| BP | GO:0097485 | 0.03437481 | 5 |
| BP | GO:0031018 | 0.03440677 | 2 |
| BP | GO:0032309 | 0.03440677 | 2 |
| BP | GO:0044275 | 0.03440677 | 2 |
| BP | GO:0048483 | 0.03440677 | 2 |
| BP | GO:1900274 | 0.03440677 | 2 |
| BP | GO:0000082 | 0.035288 | 5 |
| BP | GO:0014896 | 0.03553832 | 3 |
| BP | GO:0001935 | 0.03579683 | 4 |
| BP | GO:0001504 | 0.03582117 | 2 |
| BP | GO:0006984 | 0.03582117 | 2 |
| BP | GO:0022602 | 0.03582117 | 2 |
| BP | GO:0030810 | 0.03582117 | 2 |
| BP | GO:0035196 | 0.03582117 | 2 |
| BP | GO:0043330 | 0.03582117 | 2 |
| BP | GO:0051646 | 0.03582117 | 2 |
| BP | GO:0060711 | 0.03582117 | 2 |
| BP | GO:0071354 | 0.03582117 | 2 |
| BP | GO:1900373 | 0.03582117 | 2 |
| BP | GO:0030100 | 0.03621612 | 5 |
| BP | GO:0015918 | 0.03634885 | 3 |
| BP | GO:0017158 | 0.03634885 | 3 |
| BP | GO:0042303 | 0.03634885 | 3 |
| BP | GO:0042633 | 0.03634885 | 3 |
| BP | GO:0098693 | 0.03634885 | 3 |
| BP | GO:1901136 | 0.03638172 | 4 |
| BP | GO:1901987 | 0.03680135 | 7 |
| BP | GO:0016579 | 0.03715923 | 5 |
| BP | GO:0002223 | 0.03716888 | 3 |
| BP | GO:0048640 | 0.03716888 | 3 |
| BP | GO:0060964 | 0.03716888 | 3 |
| BP | GO:0008206 | 0.03725776 | 2 |
| BP | GO:0030574 | 0.03725776 | 2 |
| BP | GO:0050435 | 0.03725776 | 2 |
| BP | GO:0070169 | 0.03725776 | 2 |
| BP | GO:0070231 | 0.03725776 | 2 |
| BP | GO:0070849 | 0.03725776 | 2 |
| BP | GO:0120163 | 0.03725776 | 2 |
| BP | GO:1903580 | 0.03725776 | 2 |
| BP | GO:1902115 | 0.03756807 | 4 |
| BP | GO:0034248 | 0.03785804 | 7 |
| BP | GO:0051348 | 0.03811737 | 5 |
| BP | GO:0044262 | 0.03860209 | 5 |
| BP | GO:0007595 | 0.0387162 | 2 |
| BP | GO:0008089 | 0.0387162 | 2 |
| BP | GO:0045912 | 0.0387162 | 2 |
| BP | GO:0046850 | 0.0387162 | 2 |
| BP | GO:0006096 | 0.03883731 | 3 |
| BP | GO:0032612 | 0.03883731 | 3 |
| BP | GO:0002220 | 0.03968567 | 3 |
| BP | GO:0006757 | 0.03968567 | 3 |
| BP | GO:0009165 | 0.03985897 | 6 |
| BP | GO:0007162 | 0.04007892 | 5 |
| BP | GO:0010712 | 0.04019616 | 2 |
| BP | GO:0032757 | 0.04019616 | 2 |
| BP | GO:0061912 | 0.04019616 | 2 |
| BP | GO:0002761 | 0.04054341 | 3 |
| BP | GO:0010508 | 0.04054341 | 3 |
| BP | GO:0010822 | 0.04054341 | 3 |
| BP | GO:0060147 | 0.04054341 | 3 |
| BP | GO:0060966 | 0.04054341 | 3 |
| BP | GO:1900371 | 0.04054341 | 3 |
| BP | GO:0035966 | 0.04063071 | 4 |
| BP | GO:0050866 | 0.04063071 | 4 |
| BP | GO:0010632 | 0.0410824 | 5 |
| BP | GO:0030808 | 0.04141053 | 3 |
| BP | GO:1990266 | 0.04141053 | 3 |
| BP | GO:1901293 | 0.04156162 | 6 |
| BP | GO:0060425 | 0.04169729 | 2 |
| BP | GO:0070741 | 0.04169729 | 2 |
| BP | GO:0097035 | 0.04169729 | 2 |
| BP | GO:1900087 | 0.04169729 | 2 |
| BP | GO:1903793 | 0.04169729 | 2 |
| BP | GO:0016079 | 0.04228699 | 3 |
| BP | GO:0042866 | 0.04228699 | 3 |
| BP | GO:0051193 | 0.04228699 | 3 |
| CC | GO:0042383 | 2.0892E-05 | 7 |
| CC | GO:0009897 | 3.0824E-05 | 11 |
| CC | GO:0043025 | 5.602E-05 | 12 |
| CC | GO:0005788 | 0.00012353 | 9 |
| CC | GO:0005759 | 0.00014925 | 11 |
| CC | GO:0034774 | 0.00016444 | 9 |
| CC | GO:0060205 | 0.00024128 | 9 |
| CC | GO:0031983 | 0.00024659 | 9 |
| CC | GO:0090575 | 0.00051126 | 6 |
| CC | GO:0044853 | 0.00056272 | 5 |
| CC | GO:0001891 | 0.00059215 | 3 |
| CC | GO:0005942 | 0.00059215 | 3 |
| CC | GO:0032839 | 0.00098193 | 3 |
| CC | GO:0061695 | 0.0011021 | 7 |
| CC | GO:0044798 | 0.00151604 | 6 |
| CC | GO:0120111 | 0.00178368 | 4 |
| CC | GO:0005667 | 0.00187249 | 8 |
| CC | GO:0034703 | 0.00238876 | 6 |
| CC | GO:0098691 | 0.0027873 | 2 |
| CC | GO:0034705 | 0.0029028 | 4 |
| CC | GO:0031588 | 0.00323884 | 2 |
| CC | GO:0045121 | 0.00332624 | 7 |
| CC | GO:0098857 | 0.00338465 | 7 |
| CC | GO:0005796 | 0.00361047 | 4 |
| CC | GO:0030315 | 0.00400234 | 3 |
| CC | GO:0042470 | 0.00414232 | 4 |
| CC | GO:0048770 | 0.00414232 | 4 |
| CC | GO:0098589 | 0.00414833 | 7 |
| CC | GO:0005775 | 0.00419966 | 5 |
| CC | GO:0032589 | 0.00518276 | 3 |
| CC | GO:0101002 | 0.00570193 | 5 |
| CC | GO:0045171 | 0.00570742 | 3 |
| CC | GO:0005769 | 0.00587978 | 7 |
| CC | GO:0071682 | 0.00660186 | 2 |
| CC | GO:1904813 | 0.00719193 | 4 |
| CC | GO:0042629 | 0.00796257 | 2 |
| CC | GO:0032838 | 0.00922754 | 5 |
| CC | GO:0043204 | 0.00939613 | 4 |
| CC | GO:0045177 | 0.00955128 | 7 |
| CC | GO:0099056 | 0.01026377 | 3 |
| CC | GO:0034702 | 0.01072053 | 6 |
| CC | GO:0005901 | 0.01314619 | 3 |
| CC | GO:0098889 | 0.01404599 | 3 |
| CC | GO:1902495 | 0.01495553 | 6 |
| CC | GO:1990351 | 0.01666828 | 6 |
| CC | GO:0031970 | 0.01695129 | 3 |
| CC | GO:0005790 | 0.01843505 | 2 |
| CC | GO:0031904 | 0.01843505 | 2 |
| CC | GO:0035578 | 0.01852053 | 3 |
| CC | GO:0031253 | 0.01973315 | 6 |
| MF | GO:0004879 | 1.3592E-14 | 11 |
| MF | GO:0098531 | 1.3592E-14 | 11 |
| MF | GO:0003707 | 1.1037E-13 | 11 |
| MF | GO:0050662 | 1.2737E-12 | 18 |
| MF | GO:0005496 | 4.6302E-11 | 11 |
| MF | GO:0004935 | 1.0493E-09 | 6 |
| MF | GO:0019842 | 2.6669E-09 | 11 |
| MF | GO:0033218 | 2.7075E-09 | 16 |
| MF | GO:0020037 | 2.9423E-08 | 10 |
| MF | GO:0046906 | 5.8162E-08 | 10 |
| MF | GO:0042277 | 1.1578E-07 | 13 |
| MF | GO:0016705 | 1.3884E-07 | 10 |
| MF | GO:0004497 | 3.8532E-07 | 8 |
| MF | GO:0016303 | 4.2301E-07 | 4 |
| MF | GO:0035004 | 6.6121E-07 | 4 |
| MF | GO:0050661 | 1.4201E-06 | 6 |
| MF | GO:0016209 | 2.0161E-06 | 7 |
| MF | GO:0008227 | 3.0227E-06 | 6 |
| MF | GO:0052742 | 3.5514E-06 | 4 |
| MF | GO:0019825 | 4.3754E-06 | 5 |
| MF | GO:0042562 | 6.3317E-06 | 7 |
| MF | GO:0031406 | 7.1496E-06 | 9 |
| MF | GO:1901338 | 9.2561E-06 | 4 |
| MF | GO:0035257 | 9.7613E-06 | 8 |
| MF | GO:0043177 | 1.162E-05 | 9 |
| MF | GO:0030170 | 3.3272E-05 | 5 |
| MF | GO:0070279 | 3.3272E-05 | 5 |
| MF | GO:0004114 | 3.7497E-05 | 4 |
| MF | GO:0051427 | 4.0327E-05 | 8 |
| MF | GO:0004112 | 4.3267E-05 | 4 |
| MF | GO:0043560 | 4.9428E-05 | 3 |
| MF | GO:0005507 | 5.1256E-05 | 5 |
| MF | GO:0033293 | 7.5965E-05 | 5 |
| MF | GO:0030374 | 9.4664E-05 | 5 |
| MF | GO:0016307 | 0.00016361 | 3 |
| MF | GO:0001540 | 0.00019492 | 5 |
| MF | GO:0046965 | 0.00019768 | 3 |
| MF | GO:0004674 | 0.00021312 | 11 |
| MF | GO:0016614 | 0.00024807 | 6 |
| MF | GO:0016829 | 0.00030121 | 7 |
| MF | GO:0001091 | 0.00032648 | 3 |
| MF | GO:0035258 | 0.00042083 | 5 |
| MF | GO:0004601 | 0.00043688 | 4 |
| MF | GO:0042165 | 0.00054177 | 4 |
| MF | GO:0016684 | 0.00058038 | 4 |
| MF | GO:0005506 | 0.00062086 | 6 |
| MF | GO:0042974 | 0.00072267 | 3 |
| MF | GO:0005518 | 0.00114323 | 4 |
| MF | GO:0042626 | 0.00115957 | 5 |
| MF | GO:0043492 | 0.00115957 | 5 |
| MF | GO:0051721 | 0.00133807 | 3 |
| MF | GO:0016616 | 0.00135037 | 5 |
| MF | GO:0015405 | 0.00156343 | 5 |
| MF | GO:0015399 | 0.00167877 | 5 |
| MF | GO:0019902 | 0.00171296 | 6 |
| MF | GO:0070405 | 0.00173847 | 4 |
| MF | GO:0030551 | 0.00188827 | 3 |
| MF | GO:0004690 | 0.00201289 | 2 |
| MF | GO:0004691 | 0.00201289 | 2 |
| MF | GO:0004955 | 0.00201289 | 2 |
| MF | GO:0008395 | 0.00220897 | 3 |
| MF | GO:0004954 | 0.00244922 | 2 |
| MF | GO:0015643 | 0.00244922 | 2 |
| MF | GO:0019903 | 0.00275267 | 5 |
| MF | GO:0004115 | 0.00292595 | 2 |
| MF | GO:0042301 | 0.00292595 | 2 |
| MF | GO:0030331 | 0.002947 | 3 |
| MF | GO:0004712 | 0.00315236 | 3 |
| MF | GO:0042910 | 0.00344252 | 2 |
| MF | GO:0015238 | 0.00365677 | 4 |
| MF | GO:0008081 | 0.00394953 | 4 |
| MF | GO:0004029 | 0.00459295 | 2 |
| MF | GO:0004953 | 0.00459295 | 2 |
| MF | GO:0030553 | 0.00459295 | 2 |
| MF | GO:0004697 | 0.00522571 | 2 |
| MF | GO:0005159 | 0.00522571 | 2 |
| MF | GO:0070402 | 0.00522571 | 2 |
| MF | GO:0099094 | 0.00584632 | 4 |
| MF | GO:0035173 | 0.00589612 | 2 |
| MF | GO:0003713 | 0.00635728 | 7 |
| MF | GO:0047555 | 0.00660363 | 2 |
| MF | GO:0016836 | 0.00663989 | 3 |
| MF | GO:0043621 | 0.00663989 | 3 |
| MF | GO:0047485 | 0.0066639 | 4 |
| MF | GO:0016765 | 0.0073185 | 3 |
| MF | GO:0005242 | 0.00812785 | 2 |
| MF | GO:0031418 | 0.00812785 | 2 |
| MF | GO:0015278 | 0.00894352 | 2 |
| MF | GO:0017171 | 0.00906194 | 5 |
| MF | GO:0005158 | 0.0097942 | 2 |
| MF | GO:0050321 | 0.0097942 | 2 |
| MF | GO:0005179 | 0.00983453 | 4 |
| MF | GO:0005267 | 0.01011255 | 4 |
| MF | GO:0044325 | 0.01039562 | 4 |
| MF | GO:0046873 | 0.01046484 | 8 |
| MF | GO:0001228 | 0.01059963 | 8 |
| MF | GO:0001223 | 0.0106794 | 2 |
| MF | GO:0002020 | 0.01157912 | 4 |
| MF | GO:0005516 | 0.01213291 | 5 |
| MF | GO:0022804 | 0.01223667 | 7 |
| MF | GO:0015101 | 0.0125513 | 2 |
| MF | GO:0001098 | 0.01320395 | 3 |
| MF | GO:0001099 | 0.01320395 | 3 |
| MF | GO:0042623 | 0.01341516 | 6 |
| MF | GO:0016628 | 0.01353702 | 2 |
| MF | GO:0099604 | 0.01455527 | 2 |
| MF | GO:0016835 | 0.01473119 | 3 |
| MF | GO:0048029 | 0.01473119 | 3 |
| MF | GO:0031625 | 0.0147355 | 6 |
| MF | GO:0015276 | 0.01490715 | 4 |
| MF | GO:0022834 | 0.01490715 | 4 |
| MF | GO:0008514 | 0.01499302 | 5 |
| MF | GO:0016645 | 0.01560555 | 2 |
| MF | GO:1901567 | 0.01560555 | 2 |
| MF | GO:0051059 | 0.0166874 | 2 |
| MF | GO:0016247 | 0.01716631 | 4 |
| MF | GO:0051117 | 0.01749012 | 3 |
| MF | GO:0016874 | 0.01756248 | 4 |
| MF | GO:0048018 | 0.01770722 | 8 |
| MF | GO:0008519 | 0.01780034 | 2 |
| MF | GO:0044389 | 0.01923551 | 6 |
| MF | GO:0016712 | 0.02011763 | 2 |
| MF | GO:0005342 | 0.02093782 | 4 |
| MF | GO:0046943 | 0.02093782 | 4 |
| MF | GO:0005217 | 0.02132105 | 2 |
| MF | GO:0097718 | 0.02132105 | 2 |
| MF | GO:0001085 | 0.02183962 | 4 |
| MF | GO:0005261 | 0.02240535 | 6 |
| MF | GO:0005504 | 0.02255372 | 2 |
| MF | GO:0072341 | 0.02313472 | 3 |
| MF | GO:0015079 | 0.02371381 | 4 |
| MF | GO:0016229 | 0.02381519 | 2 |
| MF | GO:0016620 | 0.02381519 | 2 |
| MF | GO:0004252 | 0.02419715 | 4 |
| MF | GO:0008509 | 0.02492092 | 6 |
| MF | GO:1901681 | 0.02867055 | 5 |
| MF | GO:0016709 | 0.02914021 | 2 |
| KEGG | hsa04931 | 2.3742E-09 | 13 |
| KEGG | hsa04917 | 2.4213E-09 | 11 |
| KEGG | hsa04211 | 2.7068E-09 | 12 |
| KEGG | hsa05418 | 5.905E-09 | 14 |
| KEGG | hsa05215 | 7.4112E-09 | 12 |
| KEGG | hsa01522 | 8.3483E-09 | 12 |
| KEGG | hsa04930 | 9.8456E-09 | 9 |
| KEGG | hsa04213 | 1.031E-08 | 10 |
| KEGG | hsa04933 | 1.0549E-08 | 12 |
| KEGG | hsa04668 | 3.8612E-08 | 12 |
| KEGG | hsa04915 | 4.8137E-08 | 13 |
| KEGG | hsa04923 | 7.0803E-08 | 9 |
| KEGG | hsa04152 | 8.4023E-08 | 12 |
| KEGG | hsa05205 | 1.2582E-07 | 15 |
| KEGG | hsa05146 | 1.3767E-07 | 11 |
| KEGG | hsa05161 | 3.2059E-07 | 13 |
| KEGG | hsa01524 | 6.3513E-07 | 9 |
| KEGG | hsa04919 | 7.9182E-07 | 11 |
| KEGG | hsa01230 | 8.0291E-07 | 9 |
| KEGG | hsa05142 | 1.2858E-06 | 10 |
| KEGG | hsa04620 | 1.5395E-06 | 10 |
| KEGG | hsa04625 | 1.5395E-06 | 10 |
| KEGG | hsa05166 | 1.7567E-06 | 14 |
| KEGG | hsa05163 | 2.4209E-06 | 14 |
| KEGG | hsa04929 | 2.4698E-06 | 8 |
| KEGG | hsa04210 | 2.5382E-06 | 11 |
| KEGG | hsa04910 | 2.7281E-06 | 11 |
| KEGG | hsa05221 | 3.5161E-06 | 8 |
| KEGG | hsa05164 | 3.8839E-06 | 12 |
| KEGG | hsa04920 | 4.405E-06 | 8 |
| KEGG | hsa05222 | 4.5641E-06 | 9 |
| KEGG | hsa05230 | 4.9166E-06 | 8 |
| KEGG | hsa04722 | 5.2758E-06 | 10 |
| KEGG | hsa04657 | 5.4606E-06 | 9 |
| KEGG | hsa05223 | 6.0919E-06 | 8 |
| KEGG | hsa04932 | 6.5985E-06 | 11 |
| KEGG | hsa05131 | 6.867E-06 | 14 |
| KEGG | hsa04750 | 9.1121E-06 | 9 |
| KEGG | hsa04914 | 9.1121E-06 | 9 |
| KEGG | hsa05220 | 9.166E-06 | 8 |
| KEGG | hsa05140 | 1.0112E-05 | 8 |
| KEGG | hsa04960 | 1.0187E-05 | 6 |
| KEGG | hsa04926 | 1.0862E-05 | 10 |
| KEGG | hsa04068 | 1.2448E-05 | 10 |
| KEGG | hsa04064 | 1.2567E-05 | 9 |
| KEGG | hsa04660 | 1.2567E-05 | 9 |
| KEGG | hsa04062 | 1.2778E-05 | 12 |
| KEGG | hsa04370 | 1.5385E-05 | 7 |
| KEGG | hsa04662 | 1.6172E-05 | 8 |
| KEGG | hsa04022 | 1.8356E-05 | 11 |
| KEGG | hsa04066 | 1.8408E-05 | 9 |
| KEGG | hsa05135 | 1.8463E-05 | 10 |
| KEGG | hsa05235 | 2.957E-05 | 8 |
| KEGG | hsa04976 | 3.2085E-05 | 8 |
| KEGG | hsa05224 | 3.4056E-05 | 10 |
| KEGG | hsa04071 | 3.7213E-05 | 9 |
| KEGG | hsa04664 | 3.9457E-05 | 7 |
| KEGG | hsa04150 | 5.362E-05 | 10 |
| KEGG | hsa04218 | 5.6634E-05 | 10 |
| KEGG | hsa05160 | 5.9791E-05 | 10 |
| KEGG | hsa04380 | 6.6112E-05 | 9 |
| KEGG | hsa04913 | 6.7405E-05 | 6 |
| KEGG | hsa05167 | 6.9872E-05 | 11 |
| KEGG | hsa05214 | 7.4596E-05 | 7 |
| KEGG | hsa05212 | 8.1233E-05 | 7 |
| KEGG | hsa04151 | 0.00010221 | 15 |
| KEGG | hsa04928 | 0.00010389 | 8 |
| KEGG | hsa05225 | 0.00010568 | 10 |
| KEGG | hsa04140 | 0.000112 | 9 |
| KEGG | hsa05134 | 0.00012696 | 6 |
| KEGG | hsa05213 | 0.00014002 | 6 |
| KEGG | hsa05216 | 0.00014351 | 5 |
| KEGG | hsa05170 | 0.00016173 | 11 |
| KEGG | hsa04725 | 0.00016269 | 8 |
| KEGG | hsa04670 | 0.00017298 | 8 |
| KEGG | hsa04024 | 0.0001906 | 11 |
| KEGG | hsa00220 | 0.00021147 | 4 |
| KEGG | hsa05226 | 0.00021229 | 9 |
| KEGG | hsa05219 | 0.00023607 | 5 |
| KEGG | hsa05202 | 0.00031514 | 10 |
| KEGG | hsa04014 | 0.00035392 | 11 |
| KEGG | hsa02010 | 0.00036829 | 5 |
| KEGG | hsa04666 | 0.00037596 | 7 |
| KEGG | hsa04650 | 0.00044751 | 8 |
| KEGG | hsa04973 | 0.00045233 | 5 |
| KEGG | hsa05218 | 0.00046191 | 6 |
| KEGG | hsa05144 | 0.0006045 | 5 |
| KEGG | hsa05133 | 0.000618 | 6 |
| KEGG | hsa05165 | 0.00063248 | 13 |
| KEGG | hsa05132 | 0.0006434 | 11 |
| KEGG | hsa05162 | 0.00066458 | 8 |
| KEGG | hsa01521 | 0.00075979 | 6 |
| KEGG | hsa04012 | 0.00111784 | 6 |
| KEGG | hsa05210 | 0.00118832 | 6 |
| KEGG | hsa04935 | 0.00127747 | 7 |
| KEGG | hsa00140 | 0.00150427 | 5 |
| KEGG | hsa00250 | 0.00162706 | 4 |
| KEGG | hsa04970 | 0.00178161 | 6 |
| KEGG | hsa04020 | 0.00184221 | 9 |
| KEGG | hsa05231 | 0.00232675 | 6 |
| KEGG | hsa04015 | 0.00248373 | 9 |
| KEGG | hsa05211 | 0.00260337 | 5 |
| KEGG | hsa04080 | 0.002613 | 12 |
| KEGG | hsa04270 | 0.00263433 | 7 |
| KEGG | hsa05152 | 0.00345086 | 8 |
| KEGG | hsa04621 | 0.00356955 | 8 |
| KEGG | hsa04922 | 0.00361349 | 6 |
| KEGG | hsa05017 | 0.00363376 | 7 |
| KEGG | hsa05206 | 0.00369939 | 11 |
| KEGG | hsa04918 | 0.00374434 | 5 |
| KEGG | hsa04072 | 0.00439097 | 7 |
| KEGG | hsa05020 | 0.00449081 | 10 |
| KEGG | hsa05145 | 0.00452454 | 6 |
| KEGG | hsa04921 | 0.00545098 | 7 |
| KEGG | hsa04961 | 0.00608882 | 4 |
| KEGG | hsa04911 | 0.00670154 | 5 |
| KEGG | hsa05169 | 0.00687701 | 8 |
| KEGG | hsa05203 | 0.00728392 | 8 |
| KEGG | hsa04611 | 0.00739093 | 6 |
| KEGG | hsa04010 | 0.00748925 | 10 |
| KEGG | hsa00480 | 0.00787407 | 4 |
| KEGG | hsa00630 | 0.00802822 | 3 |
| KEGG | hsa05032 | 0.00847508 | 5 |
| KEGG | hsa01523 | 0.00879986 | 3 |
| KEGG | hsa04912 | 0.00926778 | 5 |
| KEGG | hsa05323 | 0.00926778 | 5 |
| KEGG | hsa00130 | 0.0096617 | 2 |
| KEGG | hsa04371 | 0.01177769 | 6 |
| KEGG | hsa04927 | 0.01240601 | 4 |
| KEGG | hsa04916 | 0.01295073 | 5 |
| KEGG | hsa05010 | 0.01309692 | 11 |
| KEGG | hsa00350 | 0.01329689 | 3 |
| KEGG | hsa04550 | 0.01432153 | 6 |
| KEGG | hsa05143 | 0.01432658 | 3 |
| KEGG | hsa04924 | 0.01518954 | 4 |
| KEGG | hsa04622 | 0.01594205 | 4 |
| KEGG | hsa04659 | 0.01628858 | 5 |
| KEGG | hsa00260 | 0.01768091 | 3 |
| KEGG | hsa04261 | 0.01774753 | 6 |
| KEGG | hsa04934 | 0.02051525 | 6 |
| KEGG | hsa04726 | 0.02156912 | 5 |
| KEGG | hsa01200 | 0.02380636 | 5 |
| KEGG | hsa04630 | 0.02486397 | 6 |
| KEGG | hsa00280 | 0.02859067 | 3 |
| KEGG | hsa05030 | 0.03015555 | 3 |
| KEGG | hsa00100 | 0.03078075 | 2 |
| KEGG | hsa00270 | 0.03176486 | 3 |
| KEGG | hsa04114 | 0.03327123 | 5 |
| KEGG | hsa00330 | 0.03341846 | 3 |
| KEGG | hsa04540 | 0.03359742 | 4 |
| KEGG | hsa04714 | 0.041217 | 7 |
| KEGG | hsa04070 | 0.0454703 | 4 |
| KEGG | hsa04713 | 0.0454703 | 4 |
| KEGG | hsa04730 | 0.05025877 | 3 |
| KEGG | hsa05130 | 0.05573955 | 6 |
| KEGG | hsa04510 | 0.06029724 | 6 |
| KEGG | hsa05321 | 0.06108704 | 3 |
